# Post-Fabrication Deprotection of Semiconducting Polymers for High-Performance Organic Electrochemical Transistors

**DOI:** 10.1021/jacs.6c12759

**Published:** 2026-09-17

**Authors:** Junfu Tian, Andreas W. Erhardt, Silan Zhang, Mrinalni Lyer, Weidong Li, Xian’e Li, Elena Chulanova, Benjamin J. Willner, Yuyun Yao, Weimin Zhang, Adam Marks, Rachael Found, Jin Yang, Mats Fahlman, George G. Malliaras, Iain McCulloch

**Affiliations:** † Chemistry Research Laboratory, 26524 University of Oxford, 12 Mansfield Road, Oxford OX1 3TA, UK; ‡ Andlinger Center for Energy and the Environment and Department of Electrical and Computer Engineering, 6740 Princeton University, Princeton, New Jersey 08544, United States; § Electrical Engineering Division, Department of Engineering, 67365 University of Cambridge, Electrical Engineering Building, 9 JJ Thomson Avenue, Cambridge CB3 0FA, UK; ∥ Cavendish Laboratory, University of Cambridge, JJ Thomson Avenue, Cambridge CB3 0US, UK; ⊥ Department of Materials Science and Engineering, 6429 Stanford University, Stanford, California 94305, United States; # Laboratory of Organic Electronics, Department of Science and Technology (ITN), 4566 Linköping University, Norrköping SE-60174, Sweden; ∇ Advanced Light Source, Lawrence Berkeley National Laboratory, Berkeley, California 94720, United States

## Abstract

Conventionally, the development of high-performance organic electrochemical transistor (OECT) devices heavily depends on side-chain optimization of glycolated and alkylated organic mixed ionic-electronic conducting polymers (OMIECs), which typically leads to a compromise between solubility and performance. In this study, we present a versatile design strategy employing silyl-protected side chains which can be acid-cleaved in a post-fabrication process to reveal short chains with alcohol termini, which would otherwise be unprocessable by conventional coating methods due to their insolubility. This approach decouples solubility from final device functionality, enabling solution processability during fabrication while ultimately optimizing hydrophilicity and charge transport in devices. Using PgBTTT and P100 as model polymer backbones, we demonstrate that their alcohol-functionalized analogues exhibit high μC*, excellent solvent resistance, and good electrochemical stability compared to their glycolated counterparts. Spectroelectrochemical and GIWAXS analyses reveal enhanced doping efficiency and reduced onset potentials, arising from favorable morphological reorganization upon cleavage. Overall, this work establishes alcohol functionalization as an attractive alternative to glycolation, expanding the chemical space for OMIEC device optimization and providing a platform for next-generation high-performance OECT materials.

## Graphical abstract

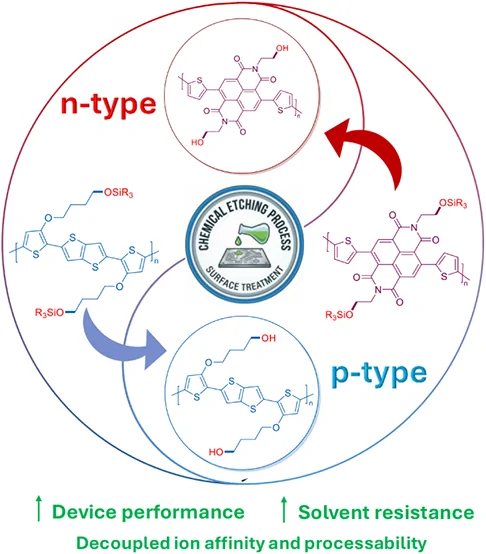

## Introduction

Organic electrochemical transistor (OECT) devices have emerged as a transformative application class, significantly advancing bioelectronics in the past decade.^1–3^ By employing organic mixed ionic-electronic conducting polymers as the channel material, OECTs efficiently transduce ionic signals from biological systems into measurable electronic output. The soft and pliable nature of OMIECs further facilitates seamless interfacing with the biological milieu. These synergistic features establish OECT as a versatile technology for next-generation bioelectronics, enabling diverse applications ranging from biosensors to wearable electronics and neuromorphic computing.^1,4^

In a conventional OECT, the semiconducting channel material is immersed in an electrolyte solution, which is sandwiched between the metal source and drain electrodes. The electrolyte is in contact with a gate electrode, applying an external bias to drive permeation of electrolyte ions into the polymer film to compensate for injected polarons in the semiconductor. Hydrated anions, such as chloride ions,^5^ diffuse more readily through hydrophilic materials. Consequently, conjugated polymers are commonly decorated with polar oligoethylene glycol side chains. Despite its generality, this strategy often requires a trial-and-error balance of side-chain architecture and length. For instance, the attachment of long glycol side chains often results in excessive swelling arising from water uptake,^6,7^ leading to disruption of electronic conductivity. As a result, this can compromise mechanical and electrochemical stability during device operation. To better control the swelling of the material, many effective strategies have been developed, including side-chain redistribution,^8^ branching,^9,10^ employing a hybrid of glycol and alkyl side chains,^11–13^ introducing an alkyl spacer,^14–17^ or using fewer hydrophilic glycol units,^18^ all of which have demonstrated an enhancement in device performance. Nonetheless, greater complexity in material architecture is associated with more cumbersome synthesis, leading to limited scalability. Furthermore, while extended side chains can improve polymer processability^19^ and sometimes ordering in film morphology,^20^ disruption of interconnectivity between charge percolating domains has been previously reported.^21^ Therefore, the design and synthesis of conjugated polymers bearing cleavable solubilizing side chains represents an attractive strategy to combine improved processability and morphology control during fabrication with enhanced hydrophilicity and conductivity after post-fabrication cleavage

Development of alternatively functionalized side chains for OECT applications has been previously reported.^22–31^ Some of these incorporate charged groups,^22,25,26,28,31^ but can electrostatically lead to stronger localization of polaronic charges^29^ and hinder charge transport. Further, ionic moieties can induce auto-doping^26^ requiring an opposite bias to restore the neutral state and can severely impact polymer solubility. Alcohol-terminated side chains^23,27,30,32,33^ have also been investigated, showing volumetric capacitance comparable to that of the tetraethylene glycol side chain^23^ while exhibiting reduced swellability^30^

Recent studies^32,33^ have demonstrated that hydroxyl functionalized conjugated polymers can exhibit attractive mixed ionic–electronic properties compared with conventional glycolated systems, yet these efforts also revealed important design trade-offs. For instance, direct synthesis of hydroxylated conjugated polymers often relied on moderately extended side chains to achieve solubility, which can compromise switching kinetics^34^ or volumetric capacitance.^24^ Moreover, most prior works have focused on impact of hydroxylation in p-type OMIECs.

Our study addresses these challenges through a polymer design featuring alcohol-terminated side chains masked by a trialkylsilyl protecting group (Figure 1a). These protecting groups provide sufficient solubility to enable solution processing, as well as good chemical stability prior to cleavage. A mild post-fabrication treatment with common, green solvents such as alcohols then unmask the alcohol termini. Importantly, this deprotection strategy is applicable to both p-type and n-type systems. These resulting hydroxylated polymers demonstrate improved performance in OECT devices, outperforming their glycolated counterparts and establishing a potentially general, versatile platform for OMIEC design. To address these challenges, we designed polymers with alcohol-terminated side chains masked by a trialkylsilyl protecting group, as illustrated in Figure 1a. These protecting groups provide sufficient solubility to enable solution processability, as well as good chemical stability prior to cleavage. A mild post-fabrication treatment can then be used to unmask alcohol termini using common green organic solvents such as alcohols. We further demonstrate that this strategy is applicable to both p-type and n-type conjugated polymers. Collectively, we present a potentially general, versatile platform for OMIEC design for OECT devices.

**Figure 1:**
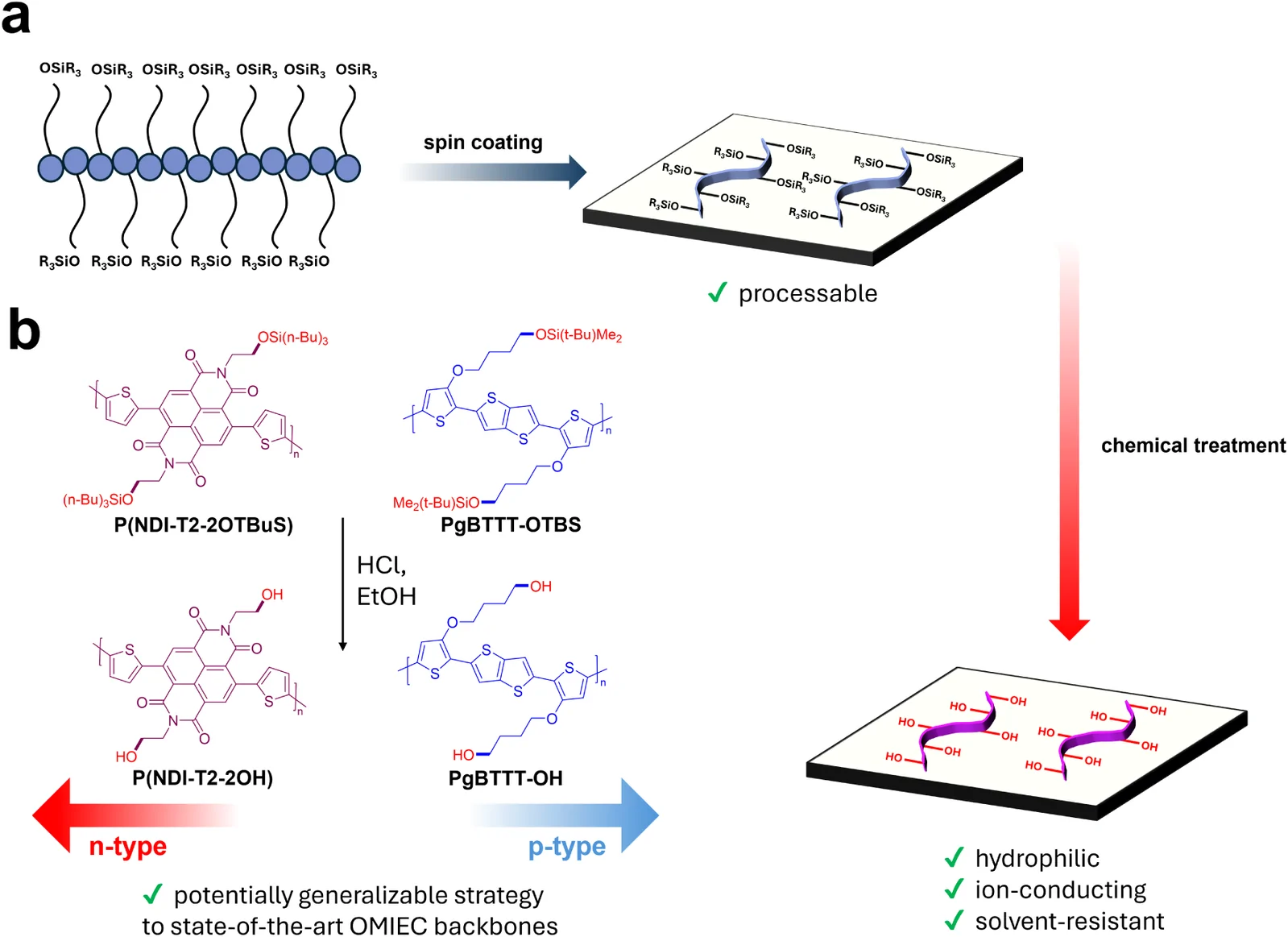
(a) Schematic representation of the chemical cleavage process used for film preparation. The polymer is depicted in a face-on orientation for illustrative purposes only and does not reflect final molecular orientation in the solid state. (b) Chemical structures of the target polymer backbones developed using this strategy for OMIEC applications.

## Result and Discussion

The polymers in this study were designed based on the state-of-the-art p-type and n-type OMIEC polymers, PgBTTT and P100. In place of conventional oligoethylene glycol side chains, silyl-protected alcohol functionalities were introduced (Figure 1b). These derivatives are subsequently referred to as PgBTTT-OTBS and P­(NDI-T2-OTBuS) respectively. In both cases, the protecting groups were designed to provide sufficient solubility to facilitate polymer synthesis and enable solution-processability, while having minimal length and being readily cleavable to furnish the desired alcohol-terminated side chains. For PgBTTT a four-carbon spacer between the deprotected alcohol and the polymer backbone was chosen as it provides both chemical stability and could be homo-coupled oxidatively,^35,36^ followed by protection with *tert*-butyldimethylsilyl (TBS) moiety. In contrast, the alkoxythiophene monomer with a two-carbon spacer exhibits poor chemical stability and appears to undergo autopolymerization spontaneously upon synthesis. Similarly, a silyl-protected 1,4,5,8-naphthalenediimide (NDI) monomer was prepared following a previous report.^37^ In this case, an ethylene spacer was used and a longer tributylsilyl group was incorporated to improve solubility of the more planar, bulky NDI core. Both monomers were then Stille-coupled with the respective comonomers to yield the target alternating polymers.

To develop an effective deprotection protocol for silyl-protected polymers, several key factors were considered. Reactions in polymer thin films are inherently heterogeneous and therefore proceed with slower kinetics than under homogeneous conditions. Consequently, side-chain cleavage must be highly efficient. In addition, the solvent must permeate the polymer thin film sufficiently to facilitate diffusion of the deprotecting reagent throughout the film. Conventional deprotection of silyl ethers typically employs a fluoride source such as tetrabutylammonium fluoride (TBAF). However, we found that residual trialkylsilyl silicon-containing byproduct is difficult to remove after treatment (Figure S37). In addition, TBAF had been previously reported as a chemical dopant for n-type polymers.^38,39^ motivating us to develop an alternative protocol. Interestingly, ethanolic hydrochloric acid, a mild and selective deprotecting agent for simple silyl groups such as *tert*-butyldimethylsilyl,^38^ triisopropylsilyl (TIPS), and diphenylmethylsilyl (DPMS),^39^ also efficiently cleaved PgBTTT-OTBS. Using ethanolic hydrochloric acid, complete deprotection was achieved within 1 h. Following deprotection, the resulting polymer thin film (subsequently referred to as PgBTTT–OH) became insoluble in chloroform, due to its reduced solubility after side-chain cleavage. Further evidence for complete deprotection was provided by X-ray photoelectron spectroscopy (XPS), where the Si 2p peak centered around 101 eV was fully absent (Figure 2a). We attribute this result to the efficient removal of the cleaved silyl-containing byproducts by the polar, lipophilic ethanol solvent. For P­(NDI-T2-2OTBuS) this mild deprotection also proved effective, despite the greater steric hindrance of the tributylsilyl substituent. Similar to PgBTTT-OTBS, the chemically treated polymer film (subsequently referred to as P­(NDI-T2-2OH) became insoluble in chloroform, while complete deprotection was further confirmed by the disappearance of the Si 2p signal in the XPS spectra, indicating the removal of silicon-containing species from the polymer film (Figure 2b). Additionally, the O 1s peak became broader following deprotection, suggesting changes in the oxygen chemical environment due to the formation of hydroxyl groups.^40^ This method could effectively permeate polymer films of at least 100 nm in thickness, as reflected from the thickness profile of the polymers (Table 1).

**Figure 2:**
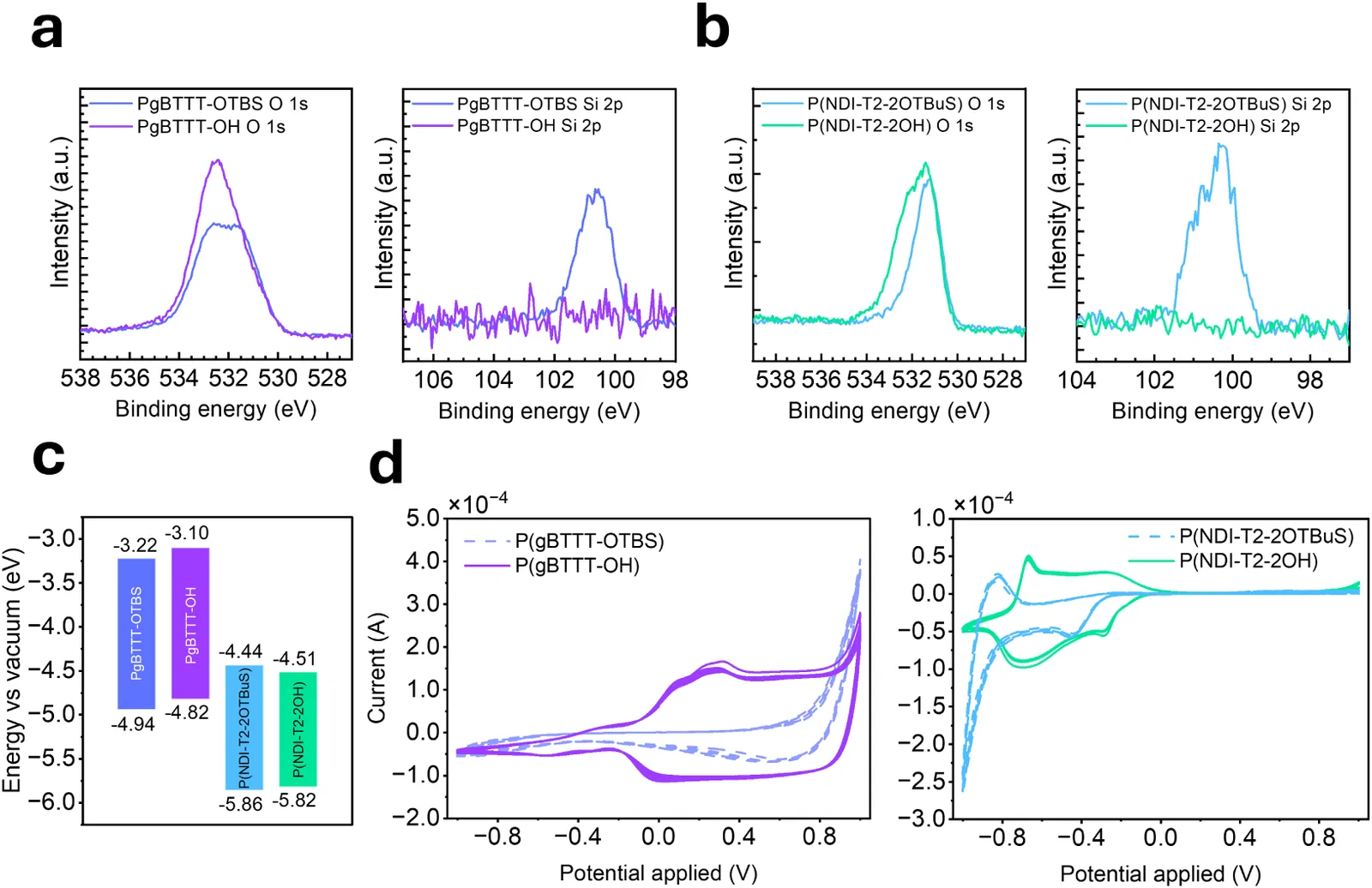
Comparison of the properties of PgBTTT-OTBS and P­(NDI-T2-2OTBuS) before and after cleavage: O 1s and Si 2p XPS spectra of (a) PgBTTT-OTBS and (b) P­(NDI-T2-2OTBuS). (c) Energy levels vs vacuum; (d) cyclic voltammograms of PgBTTT-OTBS, PgBTTT–OH, P­(NDI-T2-2OTBuS) and P­(NDI-T2-2OH) were measured in 0.1 M aqueous NaCl at a scan rate of 50 mV/s.

**Table 1:** OECT Performance Parameters and Figures-of-Merit for the Materials Investigated in This Study, Compared with Reported Values for PgBTTT^41^ and P100 ^13^

Material	Thickness (nm)	V_TH_ (V)	g_m_ ^a^ (S/cm)	μC^a^ (Fcm^–1^ V^–1^ s^–1^)	C* (Fcm^–3^)	μ (cm^2^V^–1^s^–1^)	ON-OFF ratio
PgBTTT–OH	20	–0.12 ± 0.01	641 ± 38	1127 ± 25	219	5.15 ± 0.11	1.2 × 10^4^
P(NDI-T2-2OH)	125	0.24 ± 0.02	0.29 ± 0.03	0.67 ± 0.03	361	1.86 ± 0.08 × 10^–3^	1.5 × 10^4^
PgBTTT	108	–0.24 ± 0.01	194 ± 7	502 ± 18	164	3.44 ± 0.11	-
P100	41	0.21	0.20	0.04	192	1.96 × 10^–4^	-

a The reported polymers have *M*_n_ values of 10 kDa and 7.2 kDa, corresponding to degrees of polymerization (DP) of 16 and 7, respectively. Since PgBTTT–OH and P­(NDI-T2-2OH) are not solution processable, the *M*_n_ values of their precursors, PgBTTT-OTBS and P­(NDI-T2-2OTBuS), are listed for comparison: 3.9 kDa and 10.4 kDa, corresponding to DP values of 6 and 11, respectively. The OECT parameters were measured from four devices with different geometries.

The electronic structures of both polymers were further investigated by photoemission spectroscopy in air (PESA, Figure S12) and UV-Vis absorption spectroscopy (Figure S13). A summary of the energy levels of the highest occupied molecular orbitals (HOMO) and lowest unoccupied molecular orbitals (LUMO) of the polymers is presented in Figure 2c. Both PgBTTT-OTBS and P­(NDI-T2-OTBuS) exhibit similar optical band gaps before and after deprotection, consistent with chemical modifications being confined to the pendant groups. In fact, the optical band gaps are comparable to those of their glycolated analogues.^13,41^ Because these polymer thin films operate within a 0.1 M aqueous NaCl electrolyte in OECT devices, we recorded cyclic voltammograms of PgBTTT-OTBS and P­(NDI-T2-2OTBuS), together with their deprotected counterparts, in the same electrolyte at a scan rate of 50 mV/s to evaluate the potential required for ion injection. Pristine PgBTTT-OTBS and P­(NDI-T2-2OTBuS) exhibited a large onset potential, which we attribute to their lipophilic side chains hindering effective penetration of hydrated ions within the operating window, consistent with the restricted, incomplete electrochemical doping reported for the alkylated analogue P­(a2T-TT), in contrast to the full and reversible doping observed for its glycolated counterpart p­(g2T-TT).^42^ Deprotection removes these hydrophobic pendant groups, increasing the polymers’ affinity for hydrated ions. This is reflected in the significant decrease in onset potential observed for both deprotected polymers (Figure 2d). To confirm that this shift reflects a barrier in ion-injection energetics rather than a scan-rate-dependent kinetic artifact, we performed additional CV measurements on PgBTTT-OTBS and its deprotected counterpart across scan rates from 1–1000 mV/s^–1^ (Figure S14). The onset potential changes minimally over this range for both films. As this side-chain-controlled ion-permeation mechanism is well established across structurally similar polymer backbones,^13^ we attribute the onset-potential decrease observed for the P­(NDI-T2-2OTBuS)/P­(NDI-T2-2OH) pair to the same origin.

The electrochemical behavior of the polymer films under gate bias was further investigated by spectroelectrochemistry (Figure 3a–f). Owing to the low ionization potential of PgBTTT-OTBS, a reverse bias of −0.6 V was applied to ensure that both the pristine and deprotected films were measured from their fully dedoped state. Upon increasing the applied bias, the intensity of the neutral polymers’ absorption bands gradually decreased, accompanied by the simultaneous emergence of a polaronic peak centered at 963 nm. In both cases (Figure 3a and 3b), an isosbestic point at 714 nm was observed, indicating a direct and efficient electrochemical oxidation process.^43^ Analysis of the normalized absorbance change at the characteristic peak positions as a function of applied bias (Figure 3c and 3f) revealed that deprotection significantly lowered the onset potential required for the electrochromic response. Additionally, the maximum polaronic absorption increased (Figure 3c), suggesting a higher density of hole carriers upon electrochemical oxidation. At higher bias, the polaronic absorption intensity decreased for PgBTTT–OH and deviations from the initial isosbestic point became apparent. This effect was more pronounced for PgBTTT–OH, for which a second isosbestic point emerged at 1044 nm. Simultaneously, the intensity of absorption at 1100 nm increased monotonically, suggesting the formation of a new polaronic species with an absorption maximum beyond the detection limit of 1100 nm. This behavior is consistent with the formation of bipolarons, which have been reported for PgBTTT.^44^ A more pronounced change in the electrochemical doping behavior was observed in the spectroelectrochemical response of P­(NDI-T2-2OTBuS). In contrast to PgBTTT-OTBS, the pristine film exhibited negligible spectral changes over the applied potential range, indicating limited ion injection under these conditions. Following chemical cleavage, the application of an external bias resulted in a decrease in the intensity of the absorption bands at 400 and 755 nm, accompanied by the emergence of a new peak at 495 nm, with corresponding isosbestic points observed at 451 and 561 nm. Analysis of the normalized absorbance change at the characteristic peak positions as a function of applied potential demonstrated a substantial increase in electrochemical doping efficiency, as evidenced by the significant reduction in the onset potential and increased spectroscopic response.

**Figure 3:**
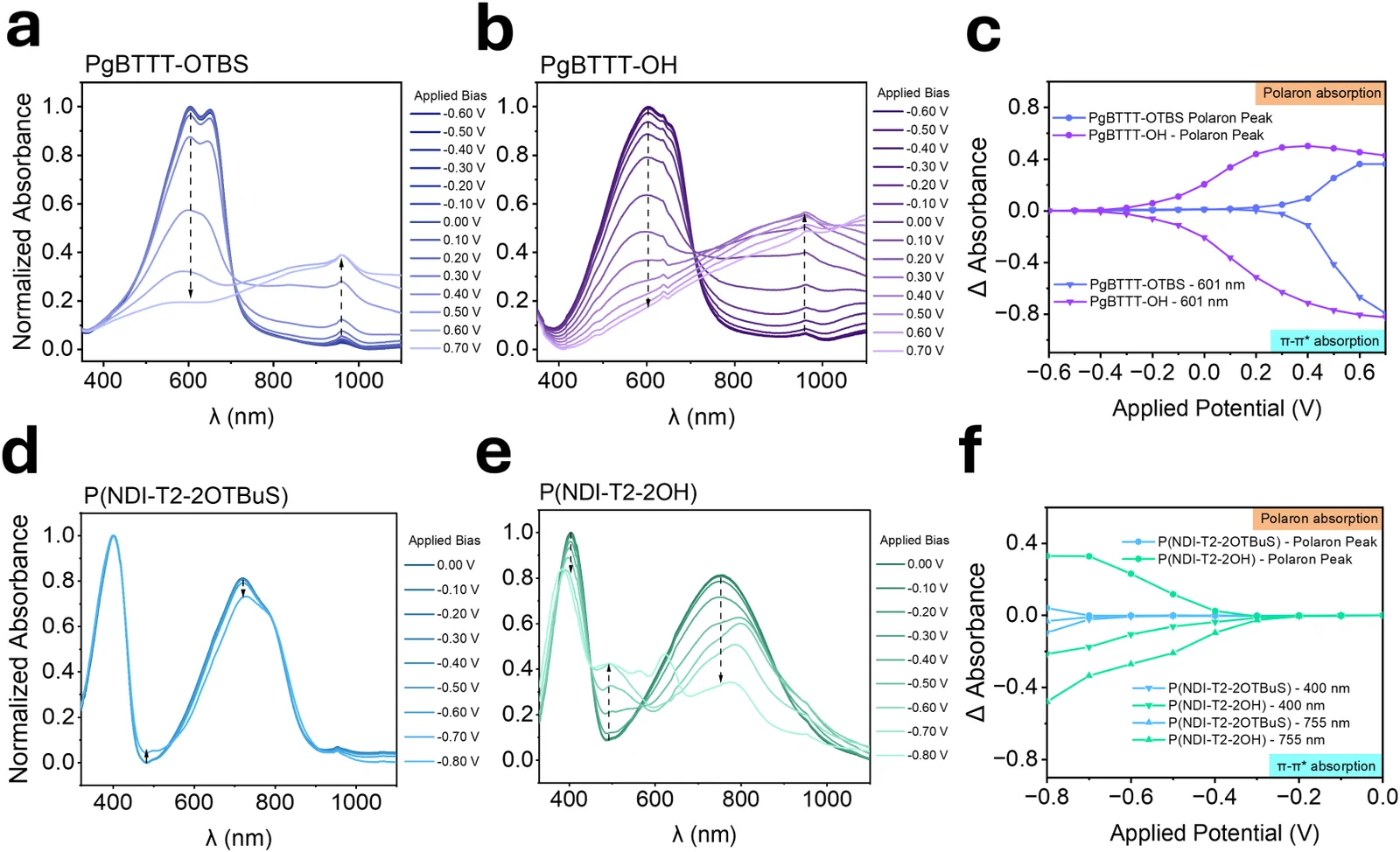
(a) and (b) present the spectroelectrochemical characterization of PgBTTT-OTBS and PgBTTT–OH. (c) shows the normalized absorbance change as a function of applied potential at the working electrode for both polymers. (d) and (e) depict the spectroelectrochemical characterization of P­(NDI-T2-2OTBuS) and P­(NDI-T2-2OH), (f) illustrates the corresponding normalized absorbance change. All measurements were conducted in 0.1 M aqueous NaCl electrolyte solution. During spectro-electrochemical measurements, the material was first subjected to a constant voltage for 10 seconds under each specific external bias before the UV–Vis spectrum was recorded, which required 2 min 30 s per measurement.

To investigate the impact of chemical cleavage on the polymer microstructure, thin films were characterized by grazing-incidence wide-angle X-ray scattering (GIWAXS). Before and after deprotection, the 2D GIWAXS patterns of PgBTTT-OTBS demonstrate a predominantly edge-on polymer orientation, characterized by three orders of out-of-plane lamellar reflection, (h00), along the q_*z*_ axis and an in-plane π–π stacking reflection, (010) along q_*xy*_ (Figure 4a). Pristine PgBTTT-OTBS exhibited lamellar and π–π stacking d-spacings of 20.3 Å and 3.6 Å, respectively. In contrast to the corresponding alkylated,^45^ alkoxylated^44^ and glycolated analogues,^41^ the lamellar reflections were predominantly out-of-plane, while the π–π stacking reflection was confined to the in-plane direction. The coexistence of a broad, weak and isotropic halo at q ∼ 1.1 Å^–1^ with these narrow, textured crystalline reflections is consistent with previous reports on silyl-functionalized conjugated polymers exhibiting edge-on orientation.^46–49^ In PgBTTT-OTBS, this halo corresponds to a d-spacing of 5.7 Å and is attributed to the local amorphous packing of the OTBS substituents. Deprotection of PgBTTT-OTBS led to several notable microstructural changes. In PgBTTT–OH, the (100) reflection shifted to a higher q value, corresponding to a reduced d-spacing of 13.2 Å, consistent with contraction of the interlamellar spacing following side-chain cleavage. This reduction in (100) d-spacing also rules out the possibility that, instead of side-chain cleavage, the difference in electrochemical behavior following chemical treatment arises from doping, because dopant intercalation had been reported to increase interlamellar distance in many conjugated polymers.^50,51^ The in-plane (010) reflection also shifted to a slightly higher q value, corresponding to a d-spacing of 3.5 Å. Together, these results indicate that deprotection alters both lamellar packing and π–π stacking, which is consistent with the enhanced electrochemical performance observed for deprotected films.

**Figure 4:**
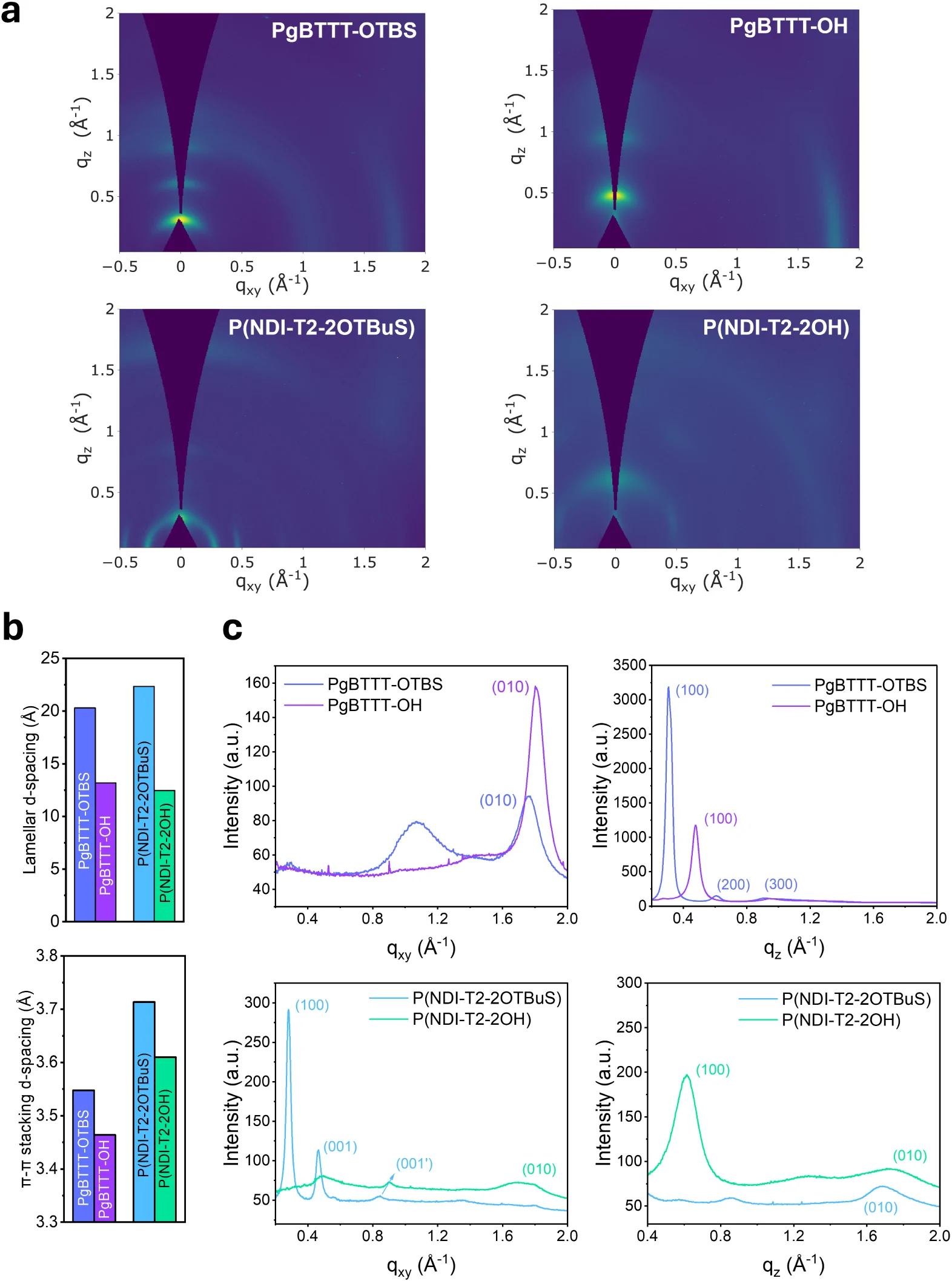
2D GIWAXS of polymer films: PgBTTT-OTBS, PgBTTT–OH, P­(NDI-T2-2OTBuS) and P­(NDI-T2-2OH). (b) Summary of lamellar d-spacing and π–π stacking d-spacing. In-plane and out-of-plane linecuts of (e) PgBTTT-OTBS and PgBTTT–OH and (f) P­(NDI-T2-2OTBuS) and P­(NDI-T2-2OH).

The structure of P­(NDI-T2-2OTBuS) exhibited predominantly face-on orientation, closely resembling that of spin-coated N2200,^13^ featuring more prominent (001)′ reflection, a characteristic feature of the Form I polymorph.^13,52^ The lamellar (100) reflection corresponds to a d-spacing of 22.1 Å in the in-plane direction, while the out-of-plane (010) π–π reflection shows a d-spacing of 3.7 Å. Deprotection induced pronounced changes in the polymer microstructure. In P­(NDI-T2-2OH), analysis of azimuthal scattering intensity (Figure S33) revealed reduced orientational anisotropy, revealing comparable intensity in the in-plane and out-of-plane directions. Relative to P­(NDI-T2-2OTBuS), this suggests a more favorable edge-on oriented polymer stacking. A contraction in lamellar d-spacing from 22.1 Å to 12.5 Å was also observed in P­(NDI-T2-2OH) following deprotection, with a slight reduction of π–π d-spacing to 3.6 Å. For both PgBTTT–OH and P­(NDI-T2-2OH), this modest contraction of the π–π stacking distance can be rationalized by the removal of steric hindrance from the tetrahedral side-chain branching of the protecting group, allowing the backbones to approach more closely. Altogether, these observations suggest that deprotection leads to closer lamellar and π–π stacking and promotes a preferential edge-on orientation of the polymer backbones within the crystalline domains, thereby improving charge-carrier transport. In the case of P­(NDI-T2-2OH), this also aligns with previous reports showing that a predominance of face-on orientation adversely affects electron mobility in N2200-based materials.^13,52^

The surface morphology of the polymer films was further investigated by atomic force microscopy (AFM). For the n-type polymer, both P­(NDI-T2-2OTBuS) and P­(NDI-T2-2OH) exhibited fibrillar surface morphologies similar to those previously reported for NDI-based polymers.^52,53^ Following deprotection, the fibrils became noticeably narrower and more densely packed, with the characteristic fibril width decreasing from approximately 20 to 10 nm (Figures S17 and S18). Fast Fourier transform (FFT) analysis of the AFM height images (Figure S20) further revealed a more pronounced preferential alignment in P­(NDI-T2-2OH), indicating increased anisotropy in the fibrillar surface morphology following side-chain cleavage. Together with the GIWAXS results, these observations suggest that deprotection promotes a more compact fibrillar morphology accompanied by substantial reorganization of the polymer microstructure.

In contrast, the surface topography of PgBTTT-OTBS and PgBTTT–OH appeared comparatively featureless in the height images. However, AFM adhesion and deformation maps revealed distinct differences in the mechanical contrast of the films. Narrow grain boundaries characterized by higher adhesion and lower deformation were clearly observed in PgBTTT–OH (Figure S16), whereas no comparable features were detected in PgBTTT-OTBS (Figure S14). These boundaries were not readily apparent in the height images but were clearly resolved in the adhesion and deformation channels owing to their distinct nanomechanical response. The emergence of these grain boundaries following deprotection is consistent with the changes in molecular packing observed by GIWAXS and suggests substantial microstructural reorganization into more distinct domains. Importantly, these AFM observations do not indicate the formation of mesopores on surface, but rather support a reorganization driven by reduced steric hindrance and enhanced side-chain polarity, which facilitates closer backbone stacking and improved ionic accessibility.

Organic electrochemical transistors (OECTs) were fabricated following previous procedures outlined in the literature.^54^ To evaluate the impact of side-chain cleavage on device performance, OECT devices fabricated with PgBTTT-OTBS and P­(NDI-T2-2OTBuS) were chemically deprotected prior to operation in 0.1 M aqueous NaCl using an Ag/AgCl side gate electrode. As illustrated in Figures 5a and 5b, both PgBTTT–OH and P­(NDI-T2-2OH) operated in accumulation mode. The corresponding transfer characteristics are presented on a linear current scale in Figure 5c and 5d. Both devices exhibited highly reversible switching with good response time (τ_on_) of 6.75 ms (Figure S30a) and 2.67 ms (Figure S30b), respectively, aligning with the performance range reported for related analogues.^55–59^ In contrast, OECTs fabricated from pristine PgBTTT-OTBS could be electrochemically switched on but exhibited significantly reduced current output. These devices also exhibited pronounced hysteresis and significantly higher threshold voltage of −0.53 V. Meanwhile, pristine P­(NDI-T2-2OTBuS) exhibited negligible current modulation or switching, even at V_GS_ of 0.8 V and therefore didn’t display transistor-like behavior under the investigated operation conditions. These observations are consistent with the corresponding cyclic voltammetry (Figure 2b) and spectroelectrochemical measurements (Figure 3), both of which highlighted a substantially higher onset potential for electrochemical doping in the protected polymers.

**Figure 5:**
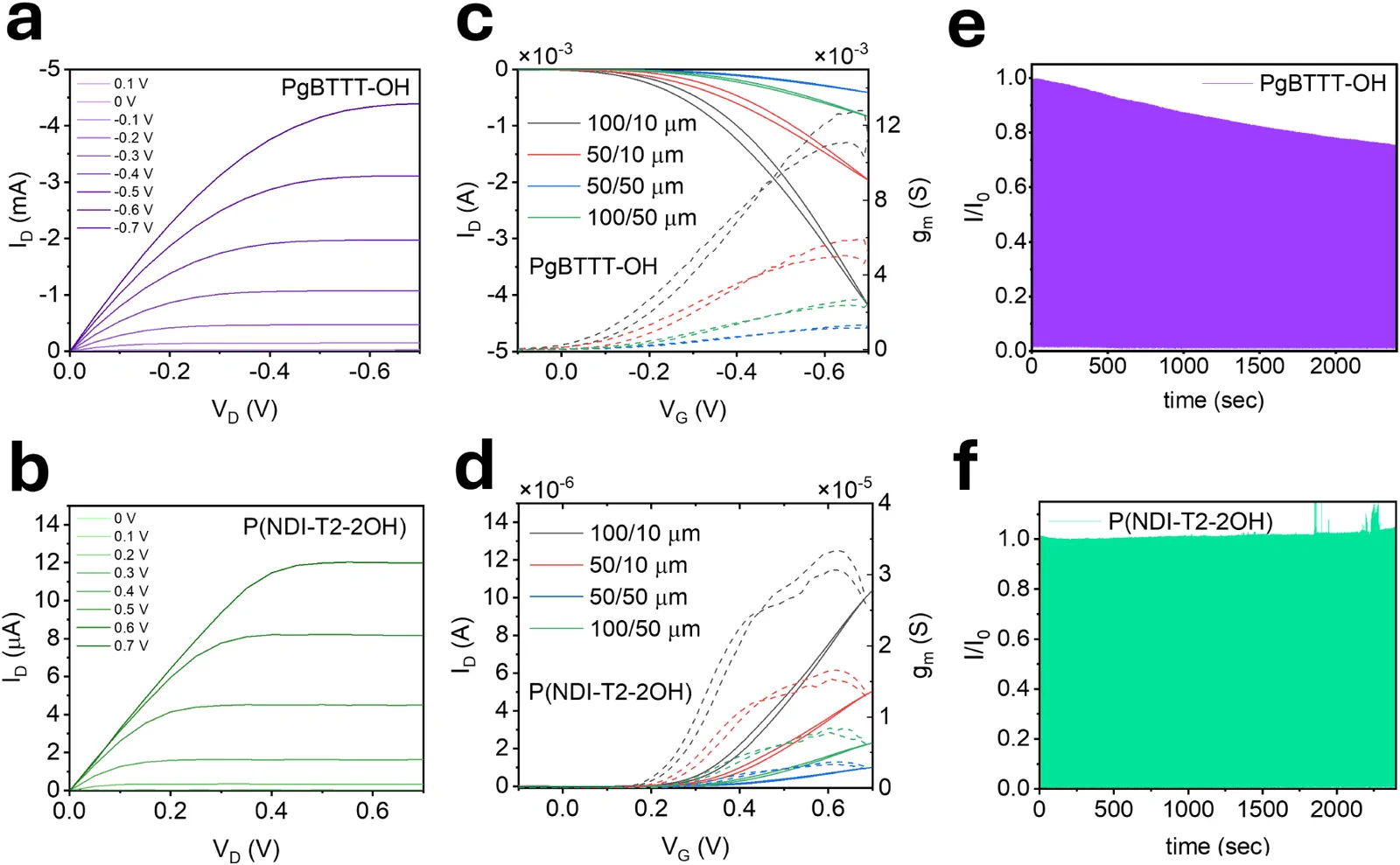
Comparison of OECT device performance between PgBTTT–OH and P­(NDI-T2-2OH): I–V output characteristics of (a) PgBTTT–OH and (b) P­(NDI-T2-2OH) OECTs with W/L=10; geometry-dependent steady-state drain current and transconductance of (c) PgBTTT–OH and (d) P­(NDI-T2-2OH) with a sweep rate of 10 mV/s; stability profile of (e) PgBTTT–OH and (f) P­(NDI-T2-2OH). For PgBTTT–OH, pulsed measurements were carried out between V_GS_ of 0 and −0.6 V, with a pulse width of 100 ms, under a V_DS_ of −0.6 V. Similarly, P­(NDI-T2-2OH) was pulsed between V_GS_ = 0 and 0.6 V with a pulse width of 100 ms, under V_DS_ = 0.6 V.

The device performance of both materials was benchmarked against their previously reported glycolated analogues, namely PgBTTT^41^ and P100.^2^ Under identical device geometry, PgBTTT–OH exhibited a thickness-normalized transconductance (g_m_*) of 640 S/cm, more than three times that of PgBTTT,^41^ as well as a remarkable μC* value of 1127 ± 25 F cm^–1^ V^–1^ s^–1,^ more than twice that reported for PgBTTT.^41^ Although higher values have been reported elsewhere,^44,56^ direct comparison is complicated by differences in device architecture and measurement conditions.^60^ PgBTTT–OH OECT also featured a low threshold voltage of −120 mV, closer to zero than that of PgBTTT, enabling low-voltage operation. A similar trend was observed for P­(NDI-T2-2OH), which exhibited a μC* of 0.67 F cm^–1^ V^–1^ s^–1^, representing an approximately 18-fold improvement over P100^13^. Both PgBTTT–OH and P­(NDI-T2-2OH) also exhibit robust device stability, maintaining 75% and nearly full retention of the initial I_DS_, respectively, even after 12000 on/off cycles (Figure 5e and 5f).

To better understand the origin of the enhanced OECT performance relative to conventional glycol polymers with identical backbones (Table 1), the product μC* was deconvoluted into its individual mobility (μ) and volumetric capacitance (C*) contributions. Volumetric capacitance was determined as a function of the redox potential using electrochemical impedance spectroscopy (EIS), as described in the SI (Figures S25–S29). Interestingly, following deprotection, PgBTTT–OH and P­(NDI-T2-2OH) demonstrated both higher μ and C* relative to their glycolated counterparts. The increase in mobility is consistent with the underlying material design strategy: shortening the side chains during the deprotection reduces the volume fraction of electrically inactive substituents while preserving the conjugated backbone responsible for charge transport. More notably, the deprotected polymers retained excellent ionic charging characteristics despite the absence of glycolated side chains. This can be rationalized by the presence of highly polar alcohol groups which preserve sufficient ion-accessible pathways, enabling effective ion ingress. Thus, conjugated polymers with a simple alcohol-functionalized side chains can achieve volumetric charging comparable to, or even exceeding, that achieved with glycolated analogues, while simultaneously supporting improved electronic transport.

## Conclusions

In this work, we have designed and synthesized silyl-protected derivatives of PgBTTT and P100 that undergo mild-acid mediated deprotection to yield alcohol-functionalized conjugated polymers. This strategy offers distinct advantages over classical glycolation, enabling solution processing of the protected materials while providing highly polar, hydrophilic side chains following post-deposition chemical treatment. As a result, PgBTTT–OH and P­(NDI-T2-2OH) exhibited enhanced electrochemical doping, improved solvent resistance, and substantially improved OECT performance relative to their protected precursors. Remarkably, both deprotected polymers outperformed their corresponding glycolated analogues, exhibiting higher charge-carrier mobility, volumetric capacitance, and consequently enhanced μC*. These findings demonstrate that simple alcohol-functionalized side chains can support efficient ionic charging while simultaneously promoting electronic transport. More broadly, this work establishes postdeposition side-chain cleavage as an effective strategy for decoupling solution processability from final device functionality and highlights alcohol-functionalized side chains as a compelling alternative to conventional glycolation for high-performance p-type and n-type organic mixed ionic-electronic conductors.

## Supplementary Material



## Notes

General methods section, synthesis of PgBTTT-OTBS and P­(NDI-T2-2OTBuS) and their monomer precursors; ^1^H and ^13^C­{^1^H} NMR, and GPC-fitted molecular weight spectra; AFM measurements and analysis; OECT devices and EIS measurements; azimuth-dependent GIWAXS analysis and summary; cyclic voltammetry; UV-Vis spectroscopy and PESA spectra, including Figures S1–S37 and Table S1–S3 (PDF)

## ○

Institut für Angewandte Physik, Technische Universität Dresden, Nöthnitzer Str. 61, 01187, Dresden, Germany

## ¶

Institute for Biomedical Innovation, Department of Engineering, University of Cambridge, 11 JJ Thomson Ave, Cambridge CB3 0FF, UK.
