# Supplementary material for: Post-Fabrication Deprotection of Semiconducting Polymers for High-Performance Organic Electrochemical Transistors
Source: J Am Chem Soc. 2026 Sep 17;148(38):41511–20. doi: 10.1021/jacs.6c12759 (PMC13632547; doi:10.1021/jacs.6c12759)
Supplement: Supplementary file 1 [file ja6c12759_si_001.pdf]

Supplementary Information for:

Post-Fabrication Deprotection of Semiconducting Polymers for High-Performance Organic Electrochemical Transistors

Junfu Tian[1], Andreas W. Erhardt[1], Silan Zhang[3,4,8], Mrinalni Lyer[5], Weidong Li[6], Xian'e Li[1,6,7], Elena Chulanova[1], Benjamin Willner[1], Yuyun Yao[1], Weimin Zhang[2], Adam Marks[5], Rachael Found[1], Jin Yang[1], Mats Fahlman[6], George G. Malliaras[3,8], Iain McCulloch[1, 2*]

[1] Chemistry Research Laboratory, University of Oxford, 12 Mansfield Road, Oxford, OX1 3TA, UK.

[2] Andlinger Center for Energy and the Environment and Department of Electrical and Computer Engineering, Princeton University, Princeton, NJ, 08544, USA.

[3] Electrical Engineering Division, Department of Engineering, University of Cambridge, Electrical Engineering Building, 9 JJ Thomson Avenue, Cambridge CB3 0FA, UK.

[4] Cavendish Laboratory, University of Cambridge, JJ Thomson Avenue, Cambridge, CB3 0US, UK.

[5] Department of Materials Science and Engineering, Stanford University, Stanford, California 94305, USA

[6] Laboratory of Organic Electronics, Department of Science and Technology (ITN), Linköping University, Norrköping SE-60174, Sweden.

[7] Advanced Light Source, Lawrence Berkeley National Laboratory, Berkeley, California 94720, USA

[8] Institute for Biomedical Innovation, Department of Engineering, University of Cambridge, 11 JJ Thomson Ave, Cambridge CB3 0FF, UK

Contents

Section 1 Experimental section 4

1.1 Materials 4

1.2 Instruments for general characterizations of chemical products 4

1.3 Procedure for film deposition and deprotection 5

1.4 Atomic Force Microscopy (AFM) 5

1.5 Cyclic Voltammetry (CV) 5

1.6 Ultraviolet-Visible (UV-Vis) Spectroscopy 5

1.7 Spectro-electrochemistry (SEC) 6

1.8 Photoemission Spectroscopy in Air (PESA) 6

1.9 Grazing Incidence Wide-Angle X-Ray Scattering (GIWAXS) 6

1.10 X-Ray Photoelectron Spectroscopy (XPS) 7

Section 2 Synthetic Procedures 8

2.1 Preparation of monomer intermediates and polymer for PgBTTT-OTBS 8

Preparation of 4-(thiophen-3-yloxy)butan-1-ol (Compound 1) 9

Preparation of Compound 2 11

Preparation of Compound 3 15

Preparation of PgBTTT-OTBS 18

2.2 Preparation of monomer intermediates and polymer for P(NDI-T2-2OTBuS) 21

Preparation of 2-((tributylsilyl)oxy)ethan-1-amine 22

Preparation of Compound 4 23

Preparation of P(NDI-T2-2OTBuS) 24

Section 3 Optoelectronic properties of the conjugated polymers 27

3.1 Photoemission Spectroscopy in Air (PESA) 28

3.2 UV-Vis Spectroscopy 29

3.3 Scan-rate dependent cyclic voltammetry of PgBTTT-OTBS/PgBTTT-OH 30

Section 4 Atomic Force Microscopy 31

Section 5 Organic Electrochemical Transistor Characterization 36

5.1 Experimental Section 36

5.2 Transfer Curve of PgBTTT-OTBS 37

5.3 Figure-of-merit Evaluation 38

5.4 Electrochemical Impedance Spectroscopy 41

5.5 Transient response measurement 45

5.6 Stability measurement 45

Section 6 Grazing Incidence Wide Angle X-ray Scattering (GIWAXS) 47

6.1 Summary table of GIWAXS scattering peaks 47

6.2 Azimuth dependence of scattering profile 48

Section 7 X-ray Photoelectron Spectroscopy (XPS) 49

Section 8 Comparison of reported materials figure of merits with state-of-the-art OMIEC materials. 52

Section 9 References 53

Section 1 Experimental section

1.1 Materials

Reagents and chemicals were purchased from commercial suppliers, including Sigma-Aldrich, FluoroChem, Alfa Aesar, Acros Organics, Manchester Organics, Insight Biotechnology Ltd, Ambeed Inc and were used as supplied unless stated otherwise. Anhydrous chlorobenzene and tetrahydrofuran were purchased from Sigma Aldrich while all remaining dry solvents were collected from an MBraun SPS800 solvent drying system. Prior to use, all glassware was oven dried at 120°C and then cooled to room temperature under reduced pressure. Thin layer chromatography (TLC) was performed on Merck aluminium silica gel 60 F254 precoated plates. Plates were visualized under an ultraviolet lamp (λ_{max} = 254nm or 365nm) and/or by staining with iodine suspended on silica, potassium permanganate solution or phosphomolybdic acid. Column chromatography was carried out on Merck Geduran Silicagel 60 (230 – 400 mesh).

1.2 Instruments for general characterizations of chemical products

^{1}H and ^{13}C nuclear magnetic resonance (*NMR*) spectra were taken on a Bruker AVIII HD 400 or AVIII HD 500 spectrometer. Chemical shifts are quoted in parts per millions (ppm) with multiplicities of the spectra recorded as the following: s, singlet; d, doublet; t, triplet; q, quartet; p, quintet; m, multiplet; br, broad; dd, doublet of doublets et cetera. Coupling constants (J) are given in Hertz (Hz) and rounded to the nearest 0.1 Hz. High resolution ESI mass spectra were recorded on a Bio-Accord LC-MS system, m/z values are reported in Daltons (Da) and high-resolution values are calculated to four decimal places from the molecular formula, with all found values in a tolerance of 5 ppm. Analytical GPC was taken on either (chloroform, via RI-detector and UV-detector with polystyrene standard) was performed on either Agilent 1200

Series GPC-SEC system using HPLC chloroform eluent at 40 °C or Agilent 1260 Infinity II using HPLC chloroform eluent at 80 °C.

1.3 Procedure for film deposition and deprotection

Polymer solution of PgBTTT-OTBS was prepared by dissolving in hot chlorobenzene (110 °C) at 5 mg mL^{-1} overnight. A 5 mg mL^{-1} solution of P(NDI-T2-2OTBuS) in chloroform was prepared similarly at room temperature. Samples on glass and ITO slides, as well as OECT devices of PgBTTT-OTBS and P(NDI-T2-2OTBuS) were prepared by spin-coating the solution at 1000 rpm and 2000 rpm for 1 minute, respectively. The polymer films were deprotected by immersing the sample in an ethanoic solution of HCl (0.57 M) at room temperature for 1 hour, which was gently rinsed with ethanol to remove residual acid.

1.4 Atomic Force Microscopy (AFM)

AFM was measured using Bruker Dimension Icon Atomic Force Microscope using PeakForce QNM mode with ScanAsyst-Air probe in an argon-filled glovebox. Both polymer samples were spin-coated from polymer solution prepared following Section 1.3 onto silicon substrates, while P(NDI-T2-2OTBuS) was spin-coated from a 5 mg mL^{-1} solution in chloroform

1.5 Cyclic Voltammetry (CV)

A three-electrode configuration was employed, comprising a polymer-coated ITO substrate as the working electrode, a platinum mesh wire as the counter electrode, and a BASi Ag/AgCl (3 M NaCl) reference electrode (MF-2052). Measurements in 0.1 M aqueous NaCl supporting electrolyte were conducted at a scan rate of 50 $mV \cdot s^{-1}$. For P(NDI-T2-2OTBuS) and P(NDI-T2-2OH), the electrolyte solution was degassed by N_2 bubbling for 30 minutes prior to measurement.

1.6 Ultraviolet-Visible (UV-Vis) Spectroscopy

Ultraviolet-visible (UV-Vis) absorption spectra were collected under ambient air and temperature conditions using a UV-1800 Shimadzu spectrophotometer with UV-Probe 2.42 software. Thin-film UV-Vis absorption spectra were measured from polymer-coated glass substrates over a wavelength range of 350–1100 nm. The optical band gap was evaluated based on the onset wavelength of absorption.

1.7 Spectro-electrochemistry (SEC)

Spectro-electrochemistry was conducted in a "Cappuccino PEC Cell" placed in a UV-1800 Shimadzu spectrophotometer. A three-electrode configuration, identical to that used in cyclic voltammetry, was connected in the cell. During each measurement, a uniform, continuous potential was applied using an AutoLab PGSTAT101 for 10 seconds to the polymer-coated ITO working electrode to allow equilibration before the optical absorption was recorded.

1.8 Photoemission Spectroscopy in Air (PESA)

PESA spectra were recorded using a KP Technology APS02 system over wavelengths of ca. 180 – 280 nm on a polymer-coated glass slide. All photoemission measurements were performed under an applied bias of -70 V. The ionization potential was evaluated from the onset energy level with respect to the cube root of the photoemission intensity.

1.9 Grazing Incidence Wide-Angle X-Ray Scattering (GIWAXS)

GIWAXS measurements were conducted at Stanford Synchrotron Radiation Lightsource, SLAC National Accelerator Laboratory, Beamline 11–3. An incident X-ray energy of 12.7 keV was used. The incidence angle α of the X-ray beam was 0.12°. This angle is below the critical angle of the silicon substrate, such that complete external reflection occurs from the silicon, but the angle is below that of the polymer film, such that the beam probes the entire thickness of the polymer film. Scattering intensity was detected with a Rayonix MX225 CCD 2D area detector. The sample-to-detector distance was calibrated with a LaB6 polycrystalline standard.

All data is reported in terms of the scattering vector, $q = (4\pi/\lambda)\sin(\theta)$, where θ is the angle of scattering. GIWAXS data were processed using a custom Python script, based on the open-source pyFAI and pygix modules. Prior to any analysis of the GIWAXS data, a polarization correction was applied during the data conversion to account for the predominantly horizontal polarization of the synchrotron X-ray beam. The out-of-plane (Qz) and in-plane (Qxy) linecuts were taken at azimuthal angle ranges off χ = 5 to 20° and 60° to 87° with respect to the Qz direction, respectively. Scattering peaks were fitted to Lorentzian and skewed Voigt models using a custom Python script. Azimuthal intensity profiles of the (100) peaks include a quantitative correction involving a factor of $|\sin(\chi)|$.

1.10 X-Ray Photoelectron Spectroscopy (XPS)

XPS measurements were performed using a Scienta-200 hemispherical analyzer with a monochromatized Al Kα source (photon energy Eph = 1486.6 eV). Spectra were calibrated by referencing the binding energy scale to the Fermi level and the Au $4f_{7/2}$ peak position of an Ar^+ ion sputter-cleaned gold foil. The analysis was conducted under ultra-high vacuum conditions, with a base pressure below 1×10^{-9} mbar.

Section 2 Synthetic Procedures

2.1 Preparation of monomer intermediates and polymer for PgBTTT-OTBS

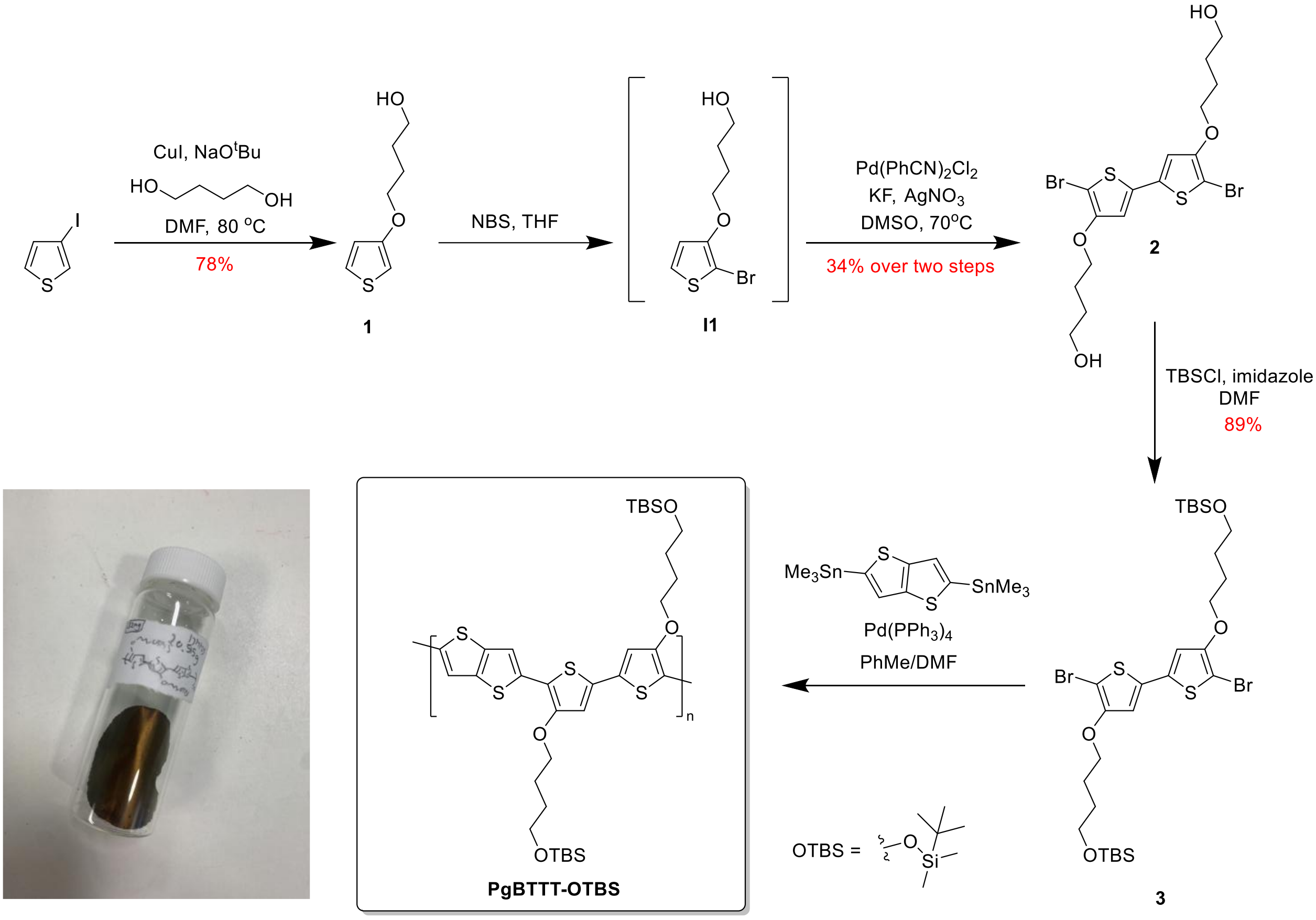

Figure S1 Overall synthetic scheme of PgBTTT-OTBS

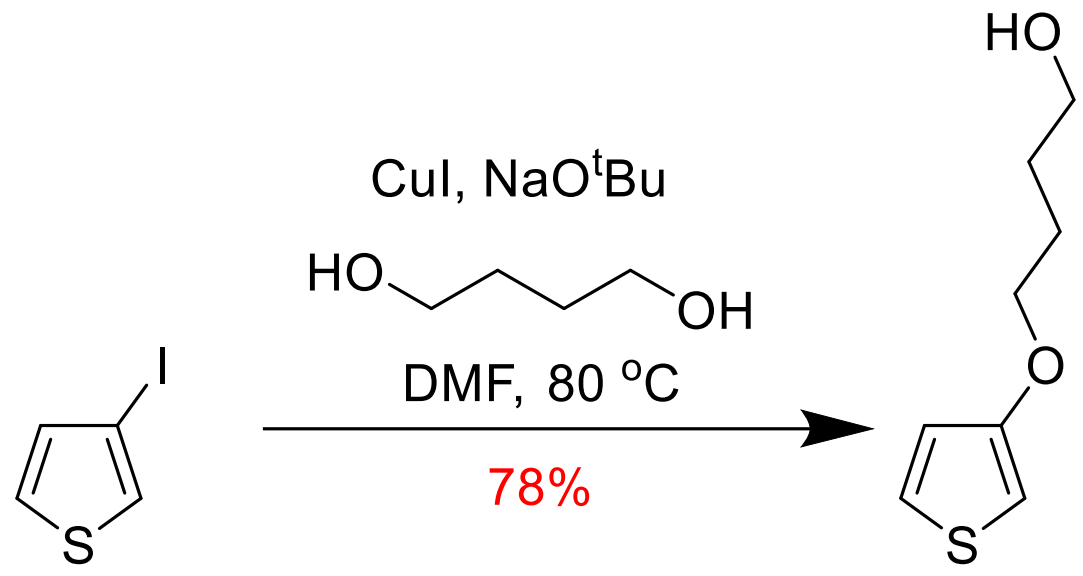

Preparation of 4-(thiophen-3-yloxy)butan-1-ol (Compound 1)

Preparation of 4-(thiophen-3-yloxy)butan-1-ol was reported previously. (https://pubs.rsc.org/en/content/articlelanding/2015/ra/c5ra12529d). Under N_2, 3-iodothiophene (3.15 g, 15 mmol), 1,4-butanediol (4.06 g, 45 mmol), cuprous iodide (256 mg, 1.5 mmol) and sodium tert-butoxide (4.32 g, 45 mmol) were dissolved in anhydrous DMF (30 mL) and heated at 80 °C for 24 hours. Then, ethyl acetate (280 mL) and water (200 mL) were added. The aqueous phase was re-extracted with ethyl acetate (200 mL x 2), which was combined with the organic phase, concentrated to a volume of 300 mL under reduced pressure and washed with brine (300 mL). Then organic layer was then dried over anhydrous sodium sulphate and concentrated under reduced pressure. The product was further purified by column chromatography (hexane/ethyl acetate 1/1) to yield 4-(thiophen-3-yloxy)butan-1-ol as a pale yellow liquid (2.01 g, 78%). ^{1}H NMR (500 MHz, CDCl3) δ 7.16 (dd, J = 5.3, 3.1 Hz, 1H), 6.74 (dd, J = 5.2, 1.6 Hz, 1H), 6.23 (dd, J = 3.2, 1.5 Hz, 1H), 3.97 (t, J = 6.2 Hz, 2H), 3.68 (t, J = 6.4 Hz, 2H), 2.42 (s, 1H), 1.85 (dq, J = 8.3, 6.4 Hz, 2H), 1.72 (dq, J = 9.5, 6.4 Hz, 2H). ^{13}C NMR (126 MHz, CDCl3) δ 157.76, 124.70, 119.46, 97.32, 70.00, 62.37, 29.38, 25.79.

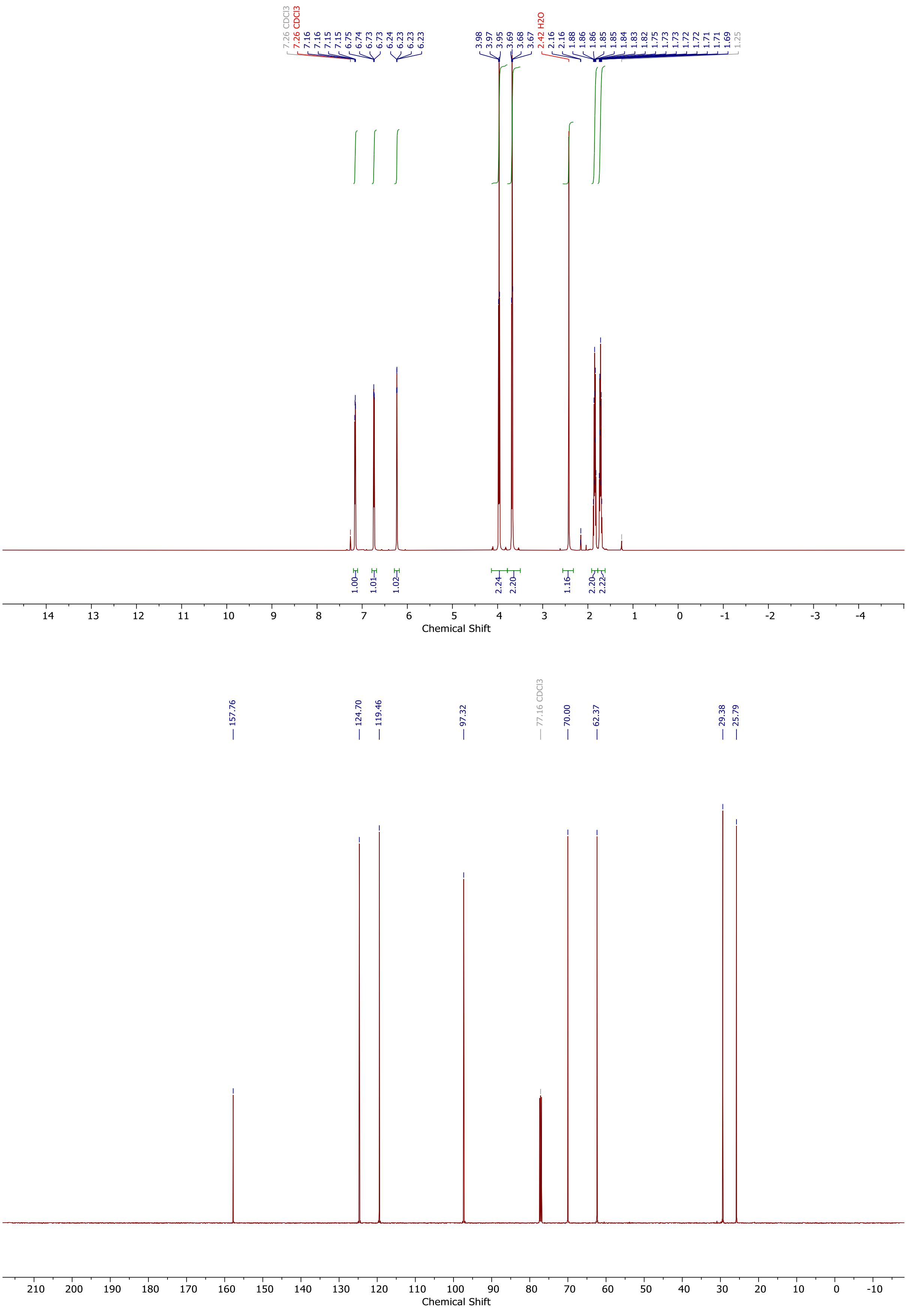

Figure S2 ^{1}H and ^{13}C NMR of 4-(thiophen-3-yloxy)butan-1-ol

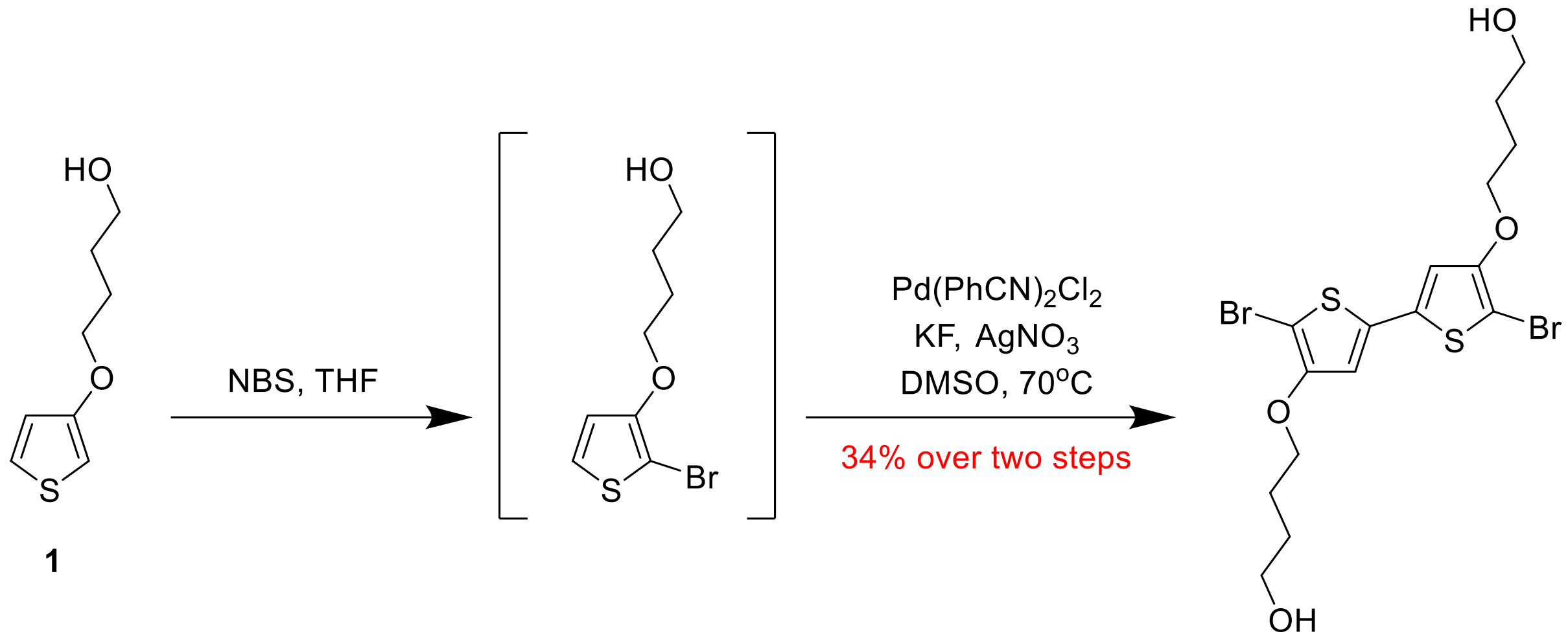

2

Preparation of Compound 2

Under N_2, 4-(thiophen-3-yloxy)butan-1-ol (2.00 g, 11.7 mmol) was dissolved in anhydrous THF (23 mL) and cooled to 0 °C. Then, N-Bromosuccinimide (2.08 g, 11.7 mmol) was added in three portions and stirred at room temperature for 2 hours. Upon completion, the reaction mixture was quenched with water (100 mL) and then extracted thrice with ethyl acetate (100 mL x 3). The organic phase was washed with brine (200 mL) and then dried over anhydrous sodium sulphate before being concentrated under reduced pressure. The crude product was further purified through column chromatography (Hexane/ethyl acetate 1/1). The intermediate **must not be immediately dried.** Anhydrous DMSO (20 mL) was added before the volatile solvent removed under reduced pressure. Then, the reaction mixture was de-gassed with N_2 bubbling for 1 hour. Silver nitrate (3.97 g, 23.4 mmol, 2 eq), anhydrous potassium fluoride (1.36 g, 23.4 mmol, 2 eq) and bis(benzonitrile)palladium dichloride ($PdCl_2(PhCN)_2$) (134 mg, 0.351 mmol, 0.03 eq) was added and the reaction mixture was warmed to 70 °C for 3 hours. Upon completion, the reaction mixture was quenched with water (80 mL). The aqueous layer was extracted with ethyl acetate (100 mL x 3), which was similarly dried following brine wash (200 mL), anhydrous sodium sulphate and column chromatography (ethyl acetate -> ethyl acetate/methanol 99/1) to yield Compound 2 as a yellow solid (995 mg, 34%). ^{1}H NMR (500 MHz, CD_3CN) δ 7.01 (s, 2H), 4.11 (t, J = 6.5 Hz, 4H), 3.55 (td, J = 6.4, 5.3 Hz, 4H), 2.60 (t, J

= 5.3 Hz, 2H), 1.77 (tt, J = 8.5, 6.3 Hz, 4H), 1.68 – 1.56 (m, 4H). ^{13}C NMR (126 MHz, CD_3CN) δ 155.83, 135.74, 115.17, 90.56, 73.05, 62.15, 29.79, 26.75. HRMS (ESI): calcd for $C_{16}H_{20}Br_2O_4S_2$, 497.9164; found 497.9183 (M^+, 3.8 ppm).

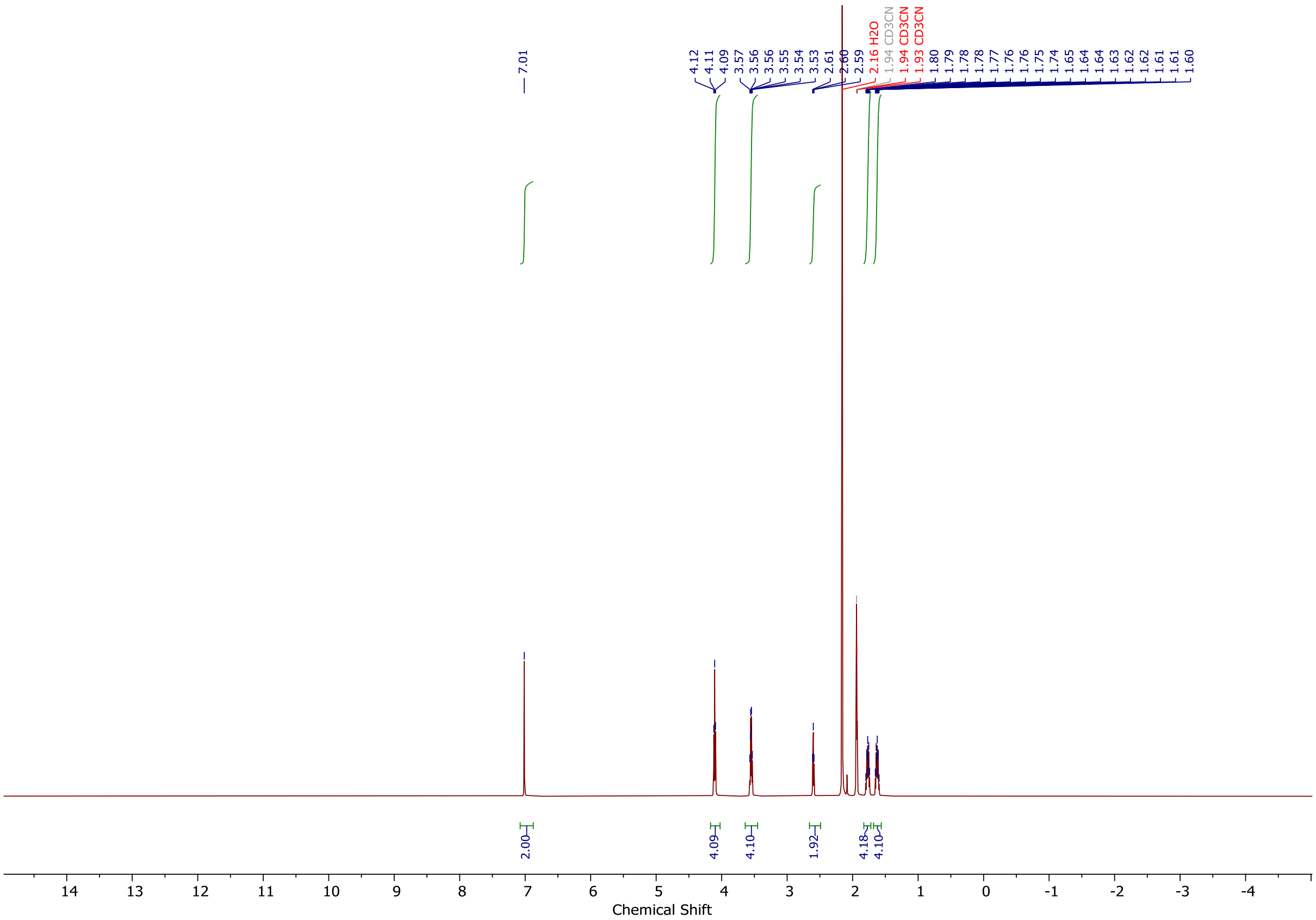

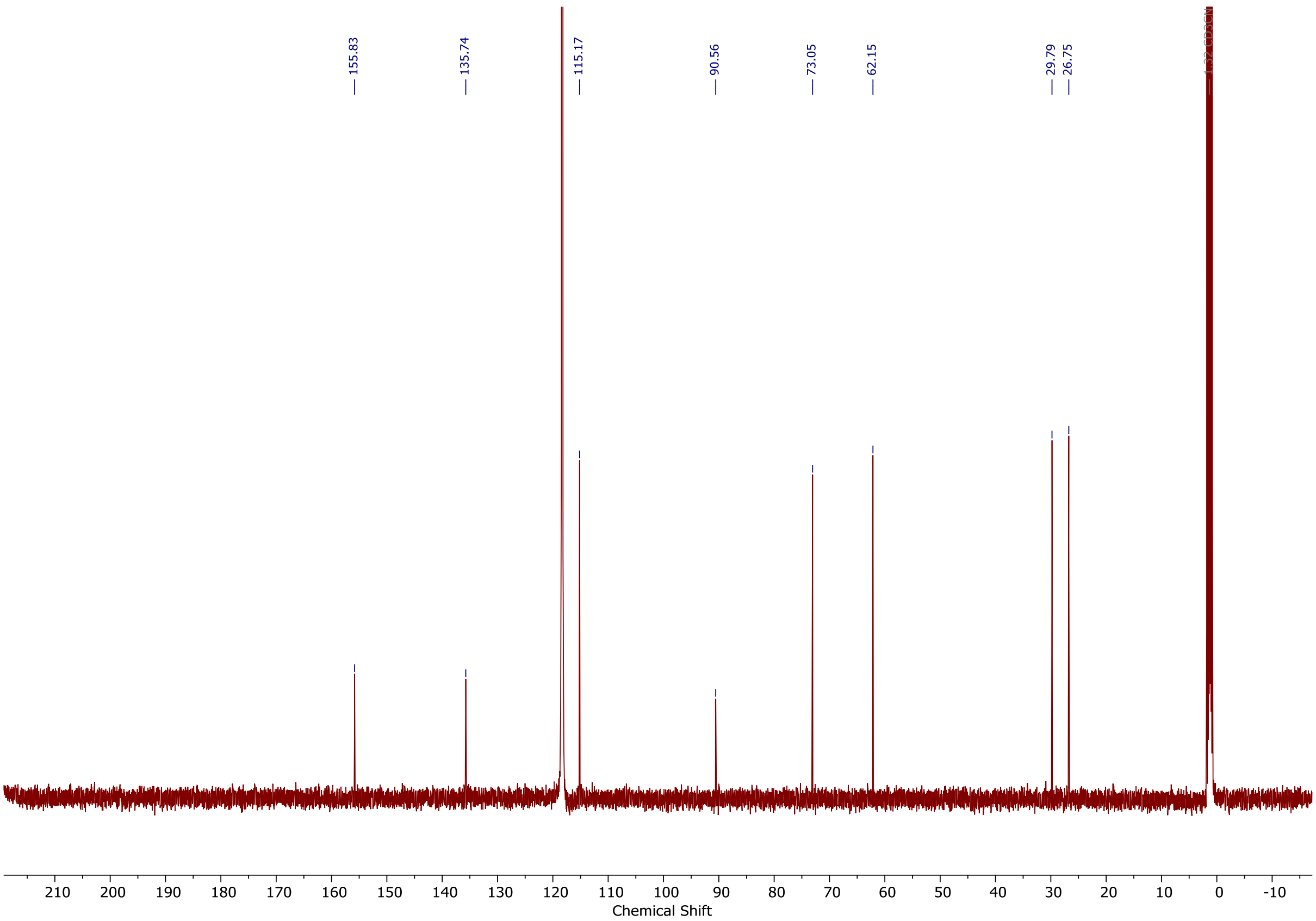

Figure S3 ^{1}H and ^{13}C NMR of Compound 2

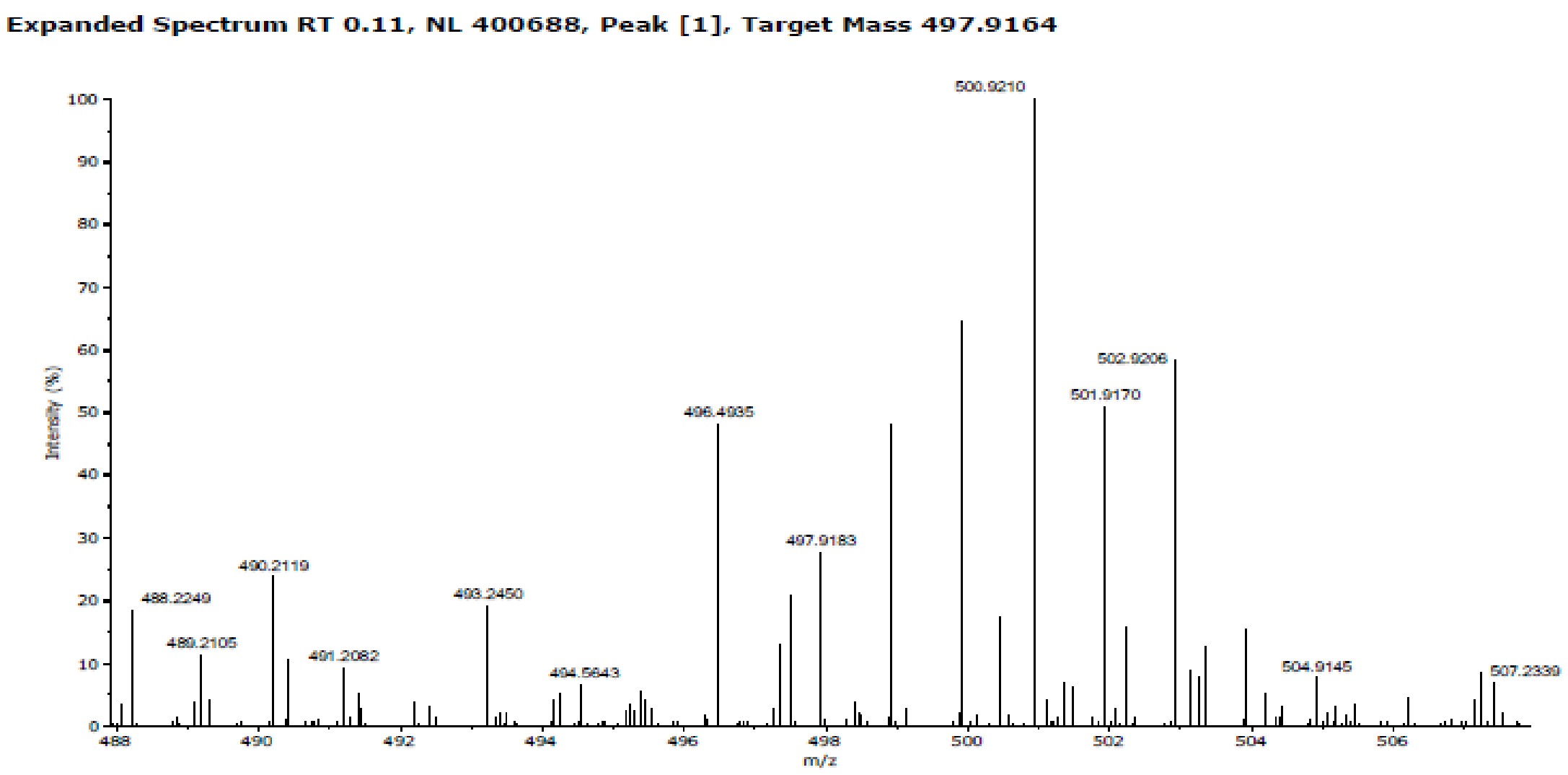

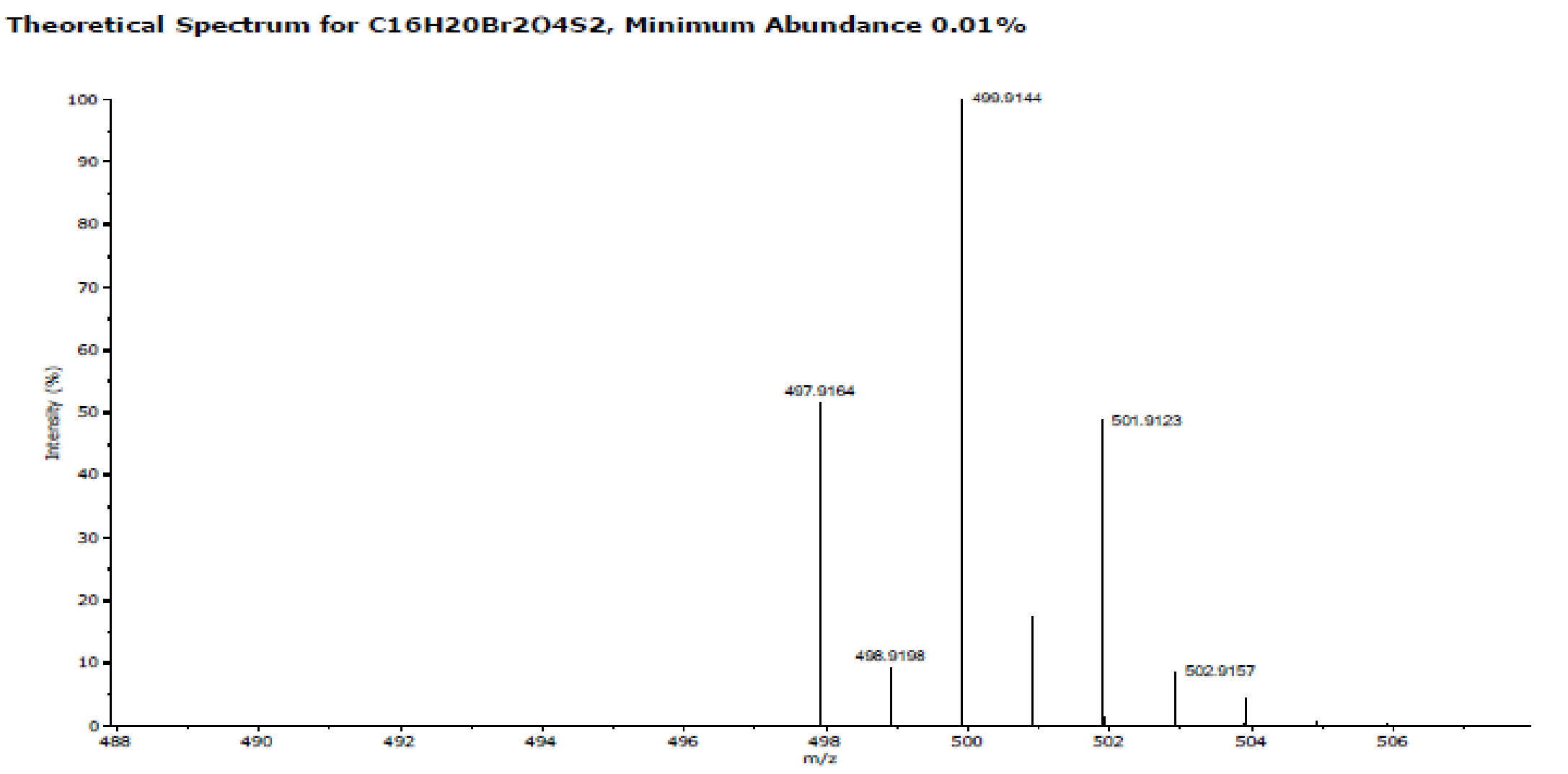

Measured Mass	Calculated Mass	Error (mDa)	Error (ppm)	Formula $[M]^+$	Response
497.9183	497.9164	1.87	3.76	$C_{16}H_{20}Br_2O_4S_2$	30108

Figure S4 HRMS of Compound 2

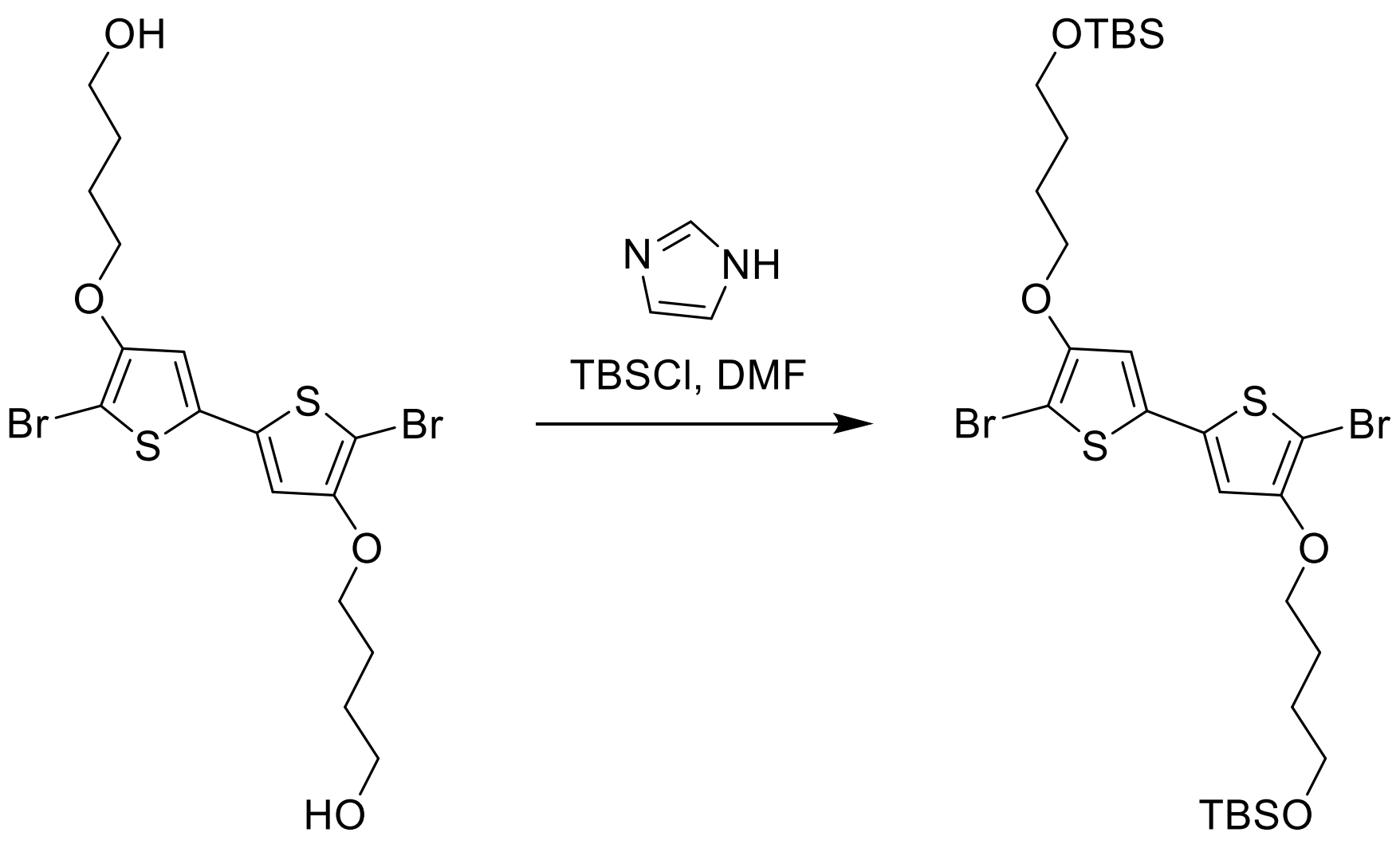

2 **3**

Preparation of Compound 3

Compound 2 (300 mg, 0.6 mmol) and tert-butyldimethylsilyl chloride (217 mg, 2.4 eq) was added to anhydrous DMF and protected from light. Then, imidazole (102 mg, 2.5 eq) was added and the reaction mixture was stirred at room temperature overnight. The reaction mixture was diluted with water (100 mL) and extracted with dichloromethane (75 mL x 3), which is then washed with water (200 mL x 4) and further purified by column chromatography (hexane -> hexane/ethyl acetate 25/1) to yield Compound 3 as yellow powder. (390 mg, 89%) ^{1}H NMR (500 MHz, CD_2Cl_2) δ 6.80 (s, 2H), 4.08 (t, J = 6.5 Hz, 4H), 3.68 (t, J = 6.2 Hz, 4H), 1.82 (dq, J = 8.8, 6.6 Hz, 4H), 1.68 (dt, J = 9.3, 6.4 Hz, 4H), 0.89 (s, 18H), 0.06 (s, 12H). ^{13}C NMR (126 MHz, CD_2Cl_2) δ 155.08, 135.30, 113.99, 90.94, 72.72, 63.09, 29.54, 26.57, 26.10, 18.58, -5.21. HRMS (ESI): calcd for $C_{28}H_{48}Br_2O_4S_2Si_2Na$, 749.0792; found 749.0772 (M+Na^+, -2.6 ppm).

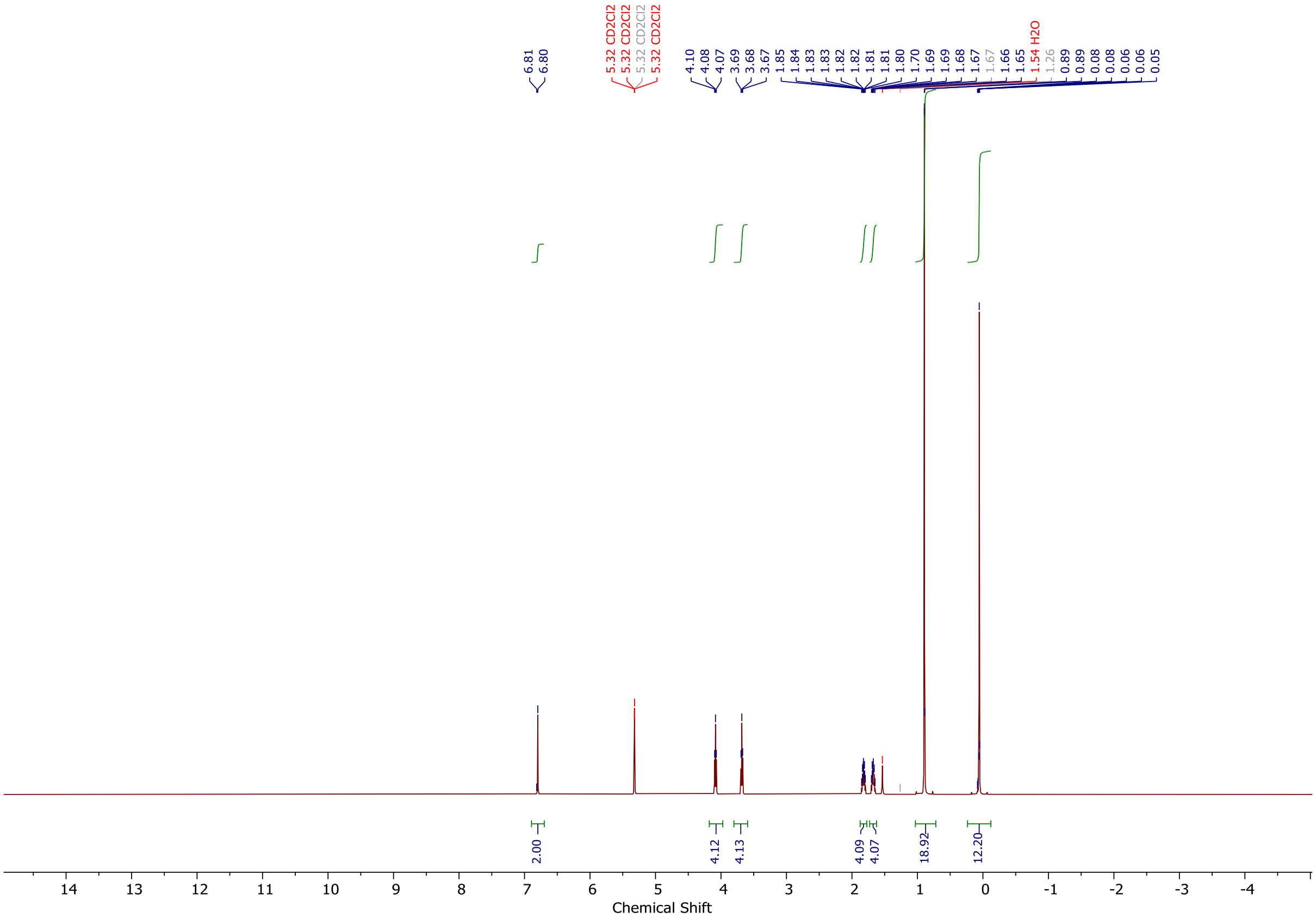

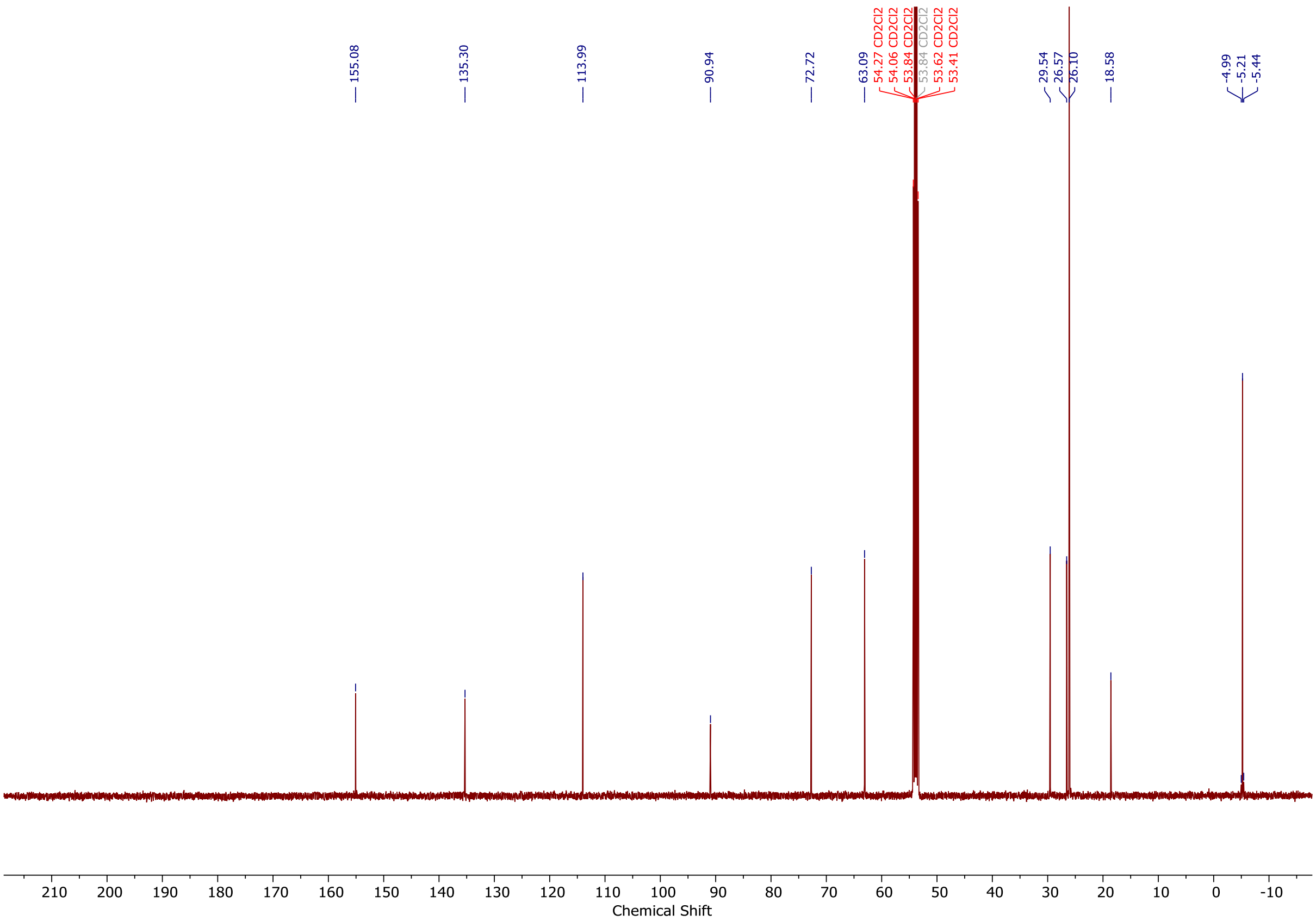

Figure S5 ^{1}H and ^{13}C NMR of Compound 3

Expanded Spectrum RT 0.09, NL 783423, Peak [1], Target Mass 749.0792

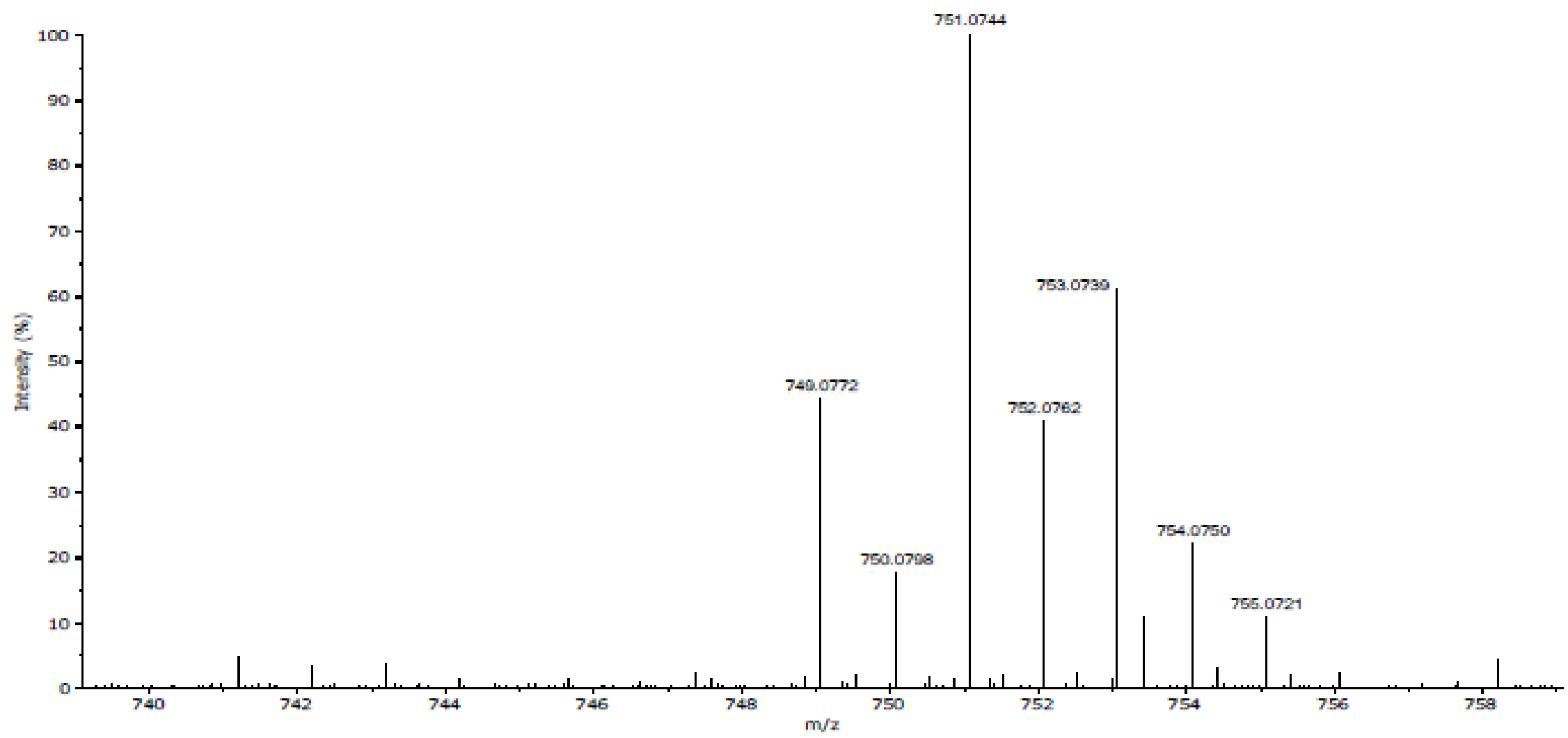

Theoretical Spectrum for C28H48Br2O4S2Si2Na, Minimum Abundance 0.01%

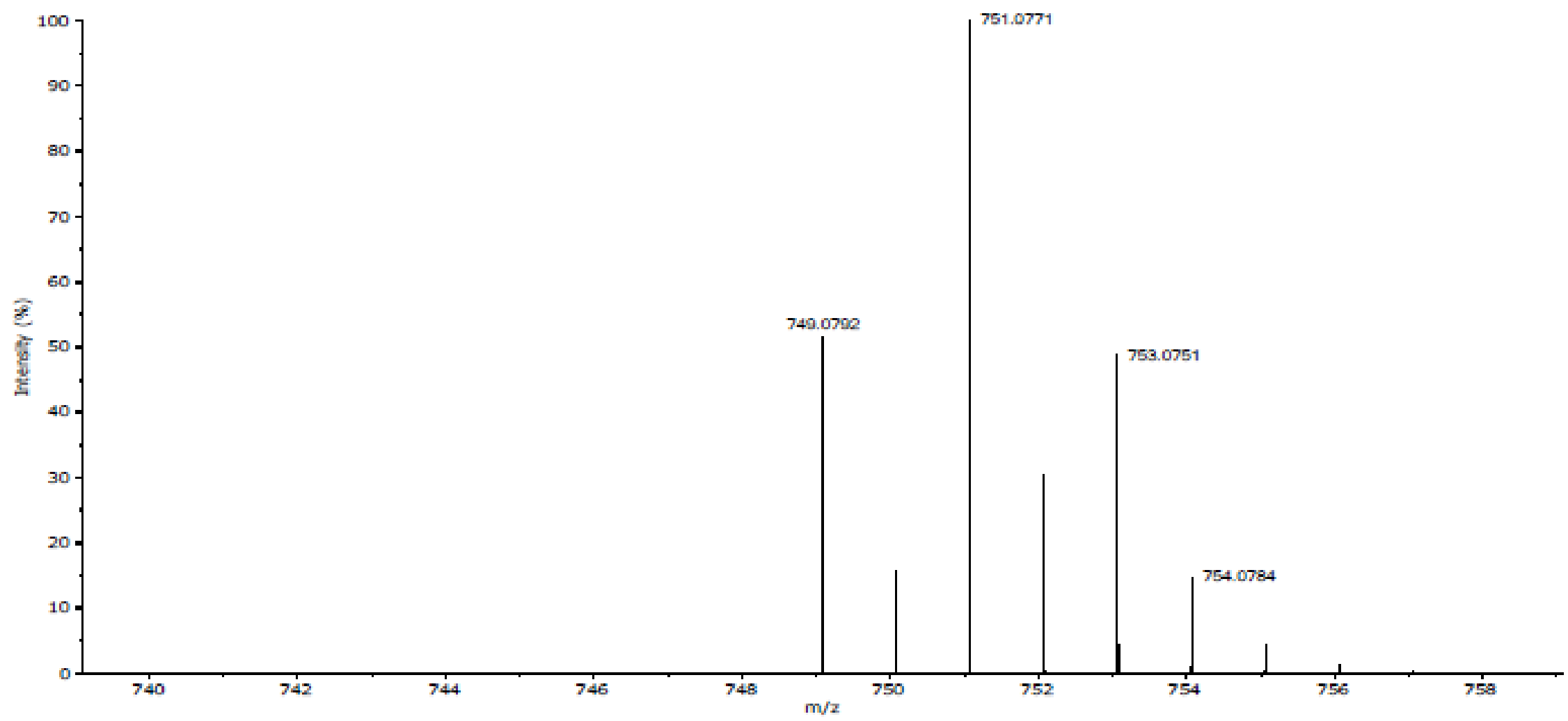

Measured Mass	Calculated Mass	Error (mDa)	Error (ppm)	Formula $[M+Na]^+$	Response
749.0772	749.0792	-1.95	-2.61	$C_{28}H_{48}Br_2O_4S_2Si_2Na$	37964

Figure S6 HRMS of Compound 3

Preparation of PgBTTT-OTBS

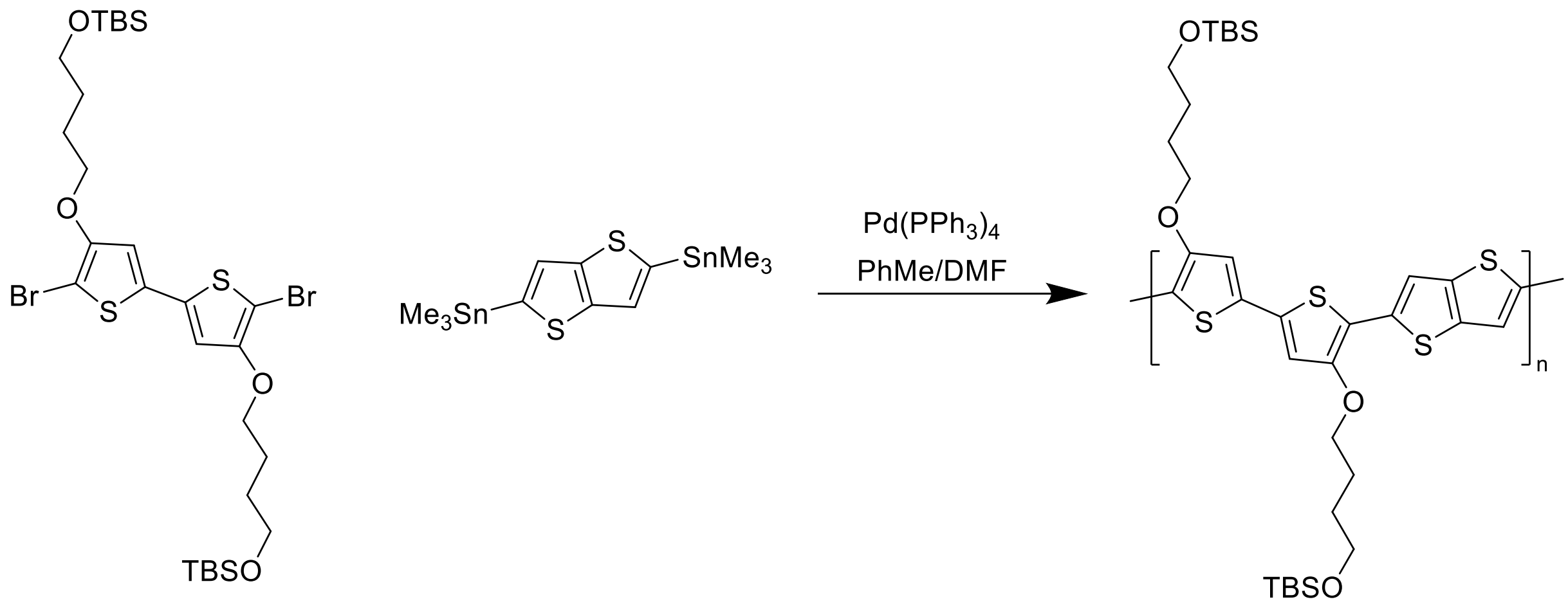

3 PgBTTT-OTBS

Under N_2, Compound 3 (145.6 mg, 0.2 mmol), 2,5-bis(trimethylstannyl)-thieno[3,2-b]thiophene (93.1 mg, 0.2 mmol) and $Pd(PPh_3)_4$ (5.78 mg, 0.005 mmol, 0.025 eq) was dissolved in a mixture of anhydrous toluene (2.4 mL) and DMF (1.6 mL). The reaction mixture was heated to 110 ºC for 24 hours, before being cooled to room temperature and precipitated in methanol. The crude polymer solid was further purified by Soxhlet extraction (methanol -> acetone -> hexane -> chloroform), concentrated and re-precipitated in methanol to yield a dark blue solid (103 mg, 70%). ^{1}H NMR (400 MHz, TCE-d_2) δ 6.41 (m, 2H), 3.29 (m, 8H), 1.80 – 0.78 (m, 8H), 0.28 (s, 18H), -0.56 (s, 12H). GPC (PhCl): M_n = 3.9 kDa, M_w = 7.3 kDa

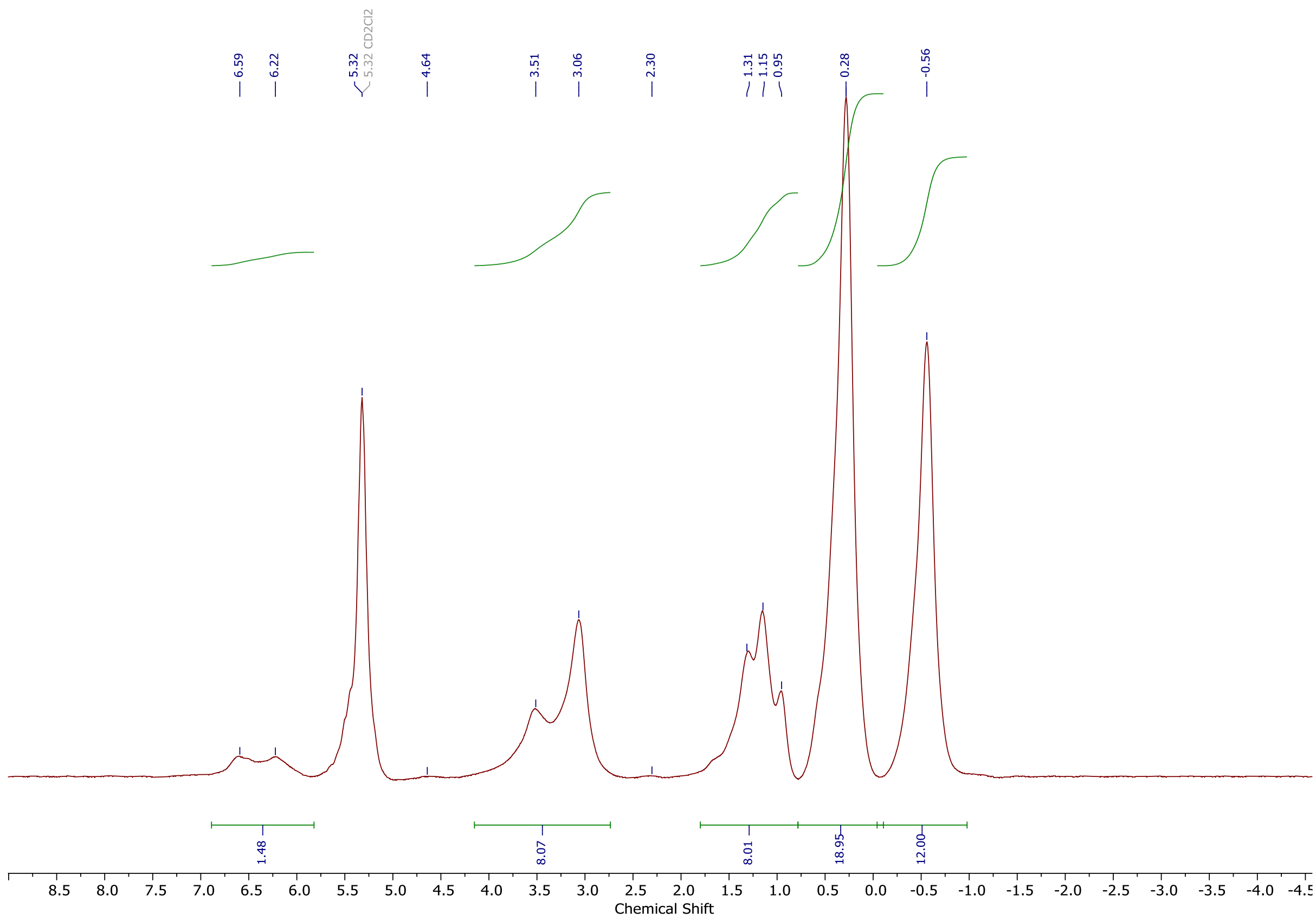

Figure S7 ^{1}H NMR of PgBTTT-OTBS

Molecular Weight Averages

Peak	Mp (g/mol)	Mn (g/mol)	Mw (g/mol)	Mz (g/mol)	Mz+1 (g/mol)	Mv (g/mol)
Peak 1	8264	3889	7347	11608	16798	6807

PD
1.889

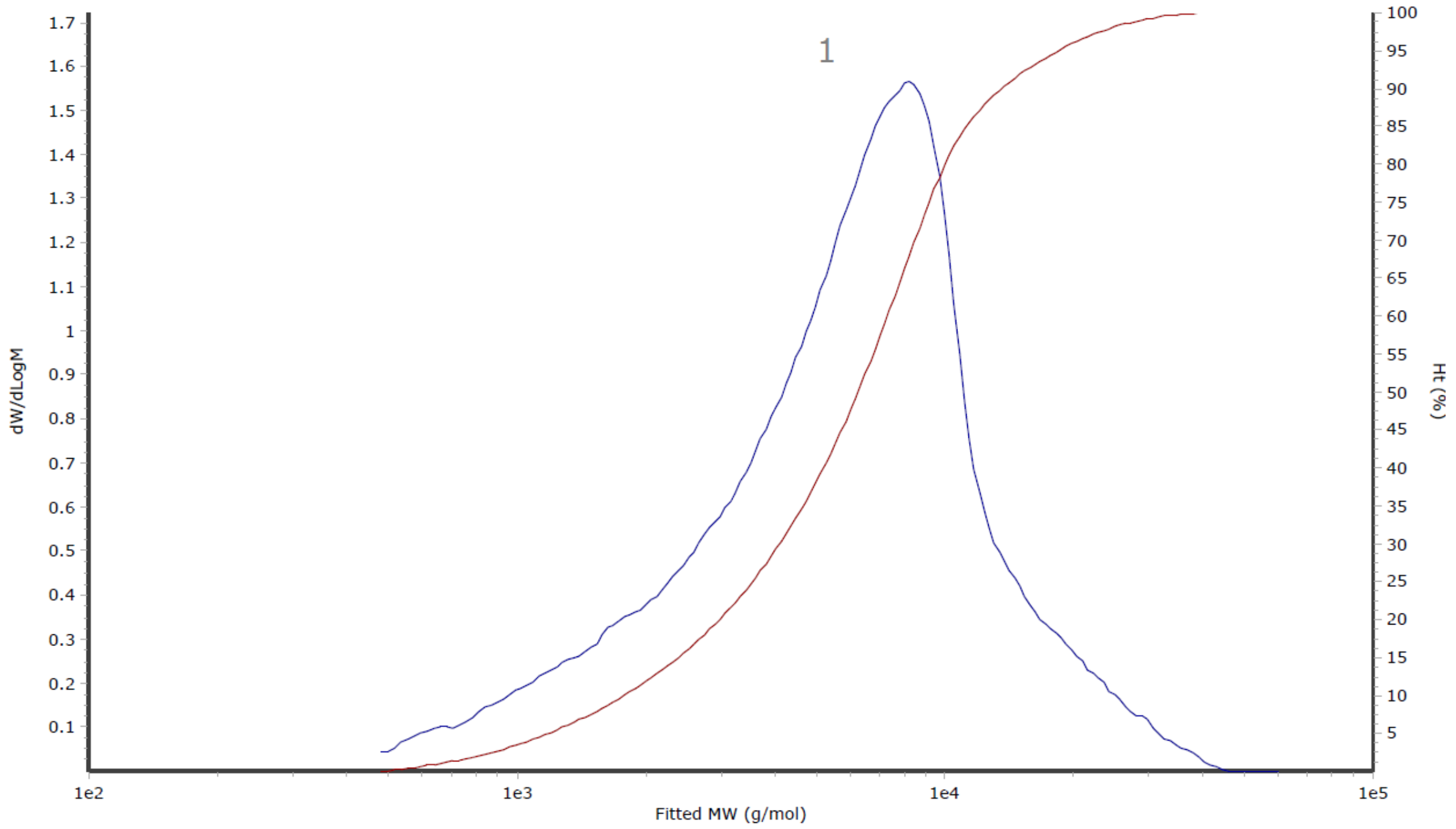

Figure S8 GPC plot of PgBTTT-OTBS

2.2 Preparation of monomer intermediates and polymer for P(NDI-T2-2OTBuS)

Monomer synthesis was conducted as previously reported. [1]

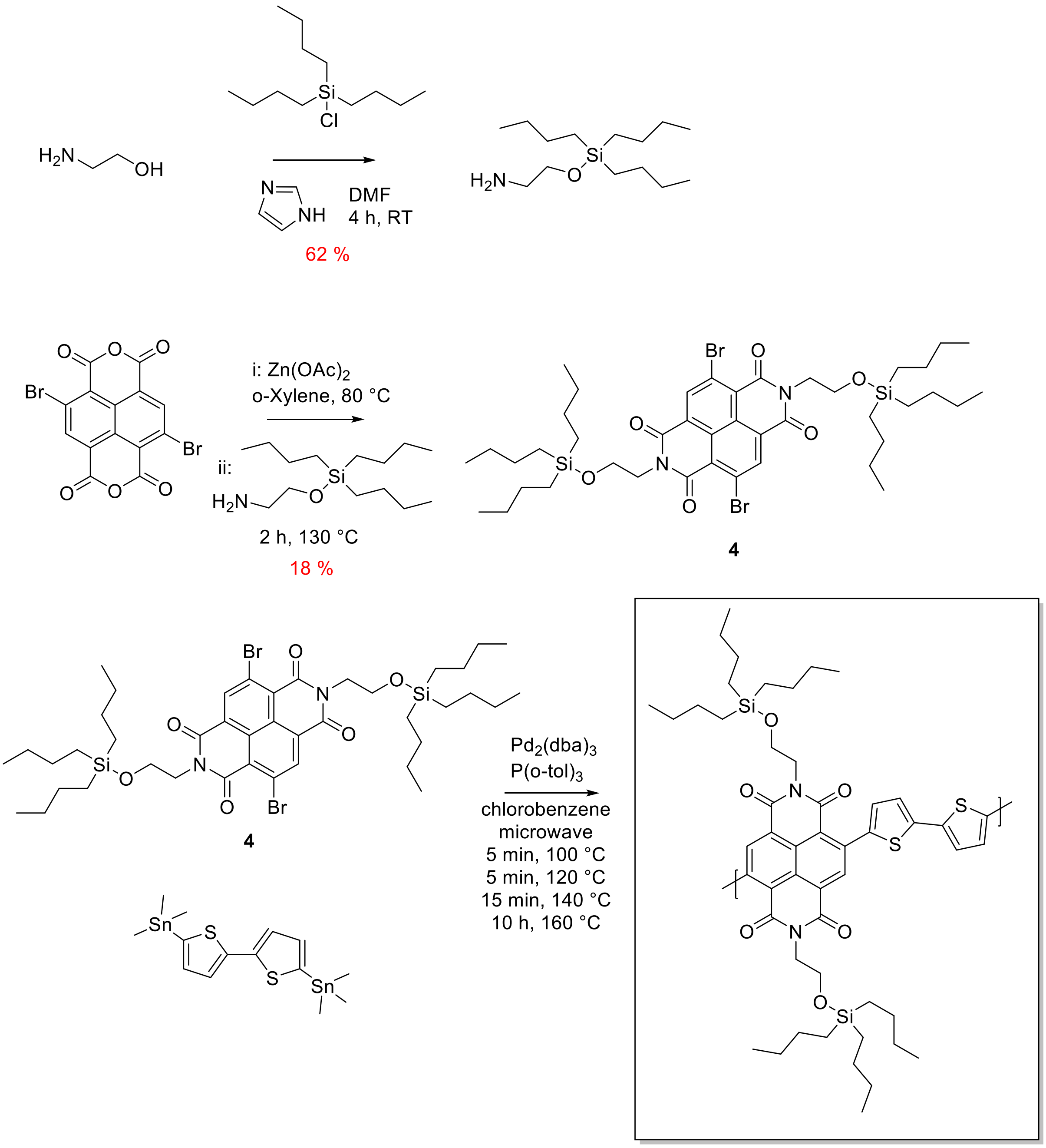

Figure S9. Overall synthetic scheme of P(NDI-T2-2OTBuS)

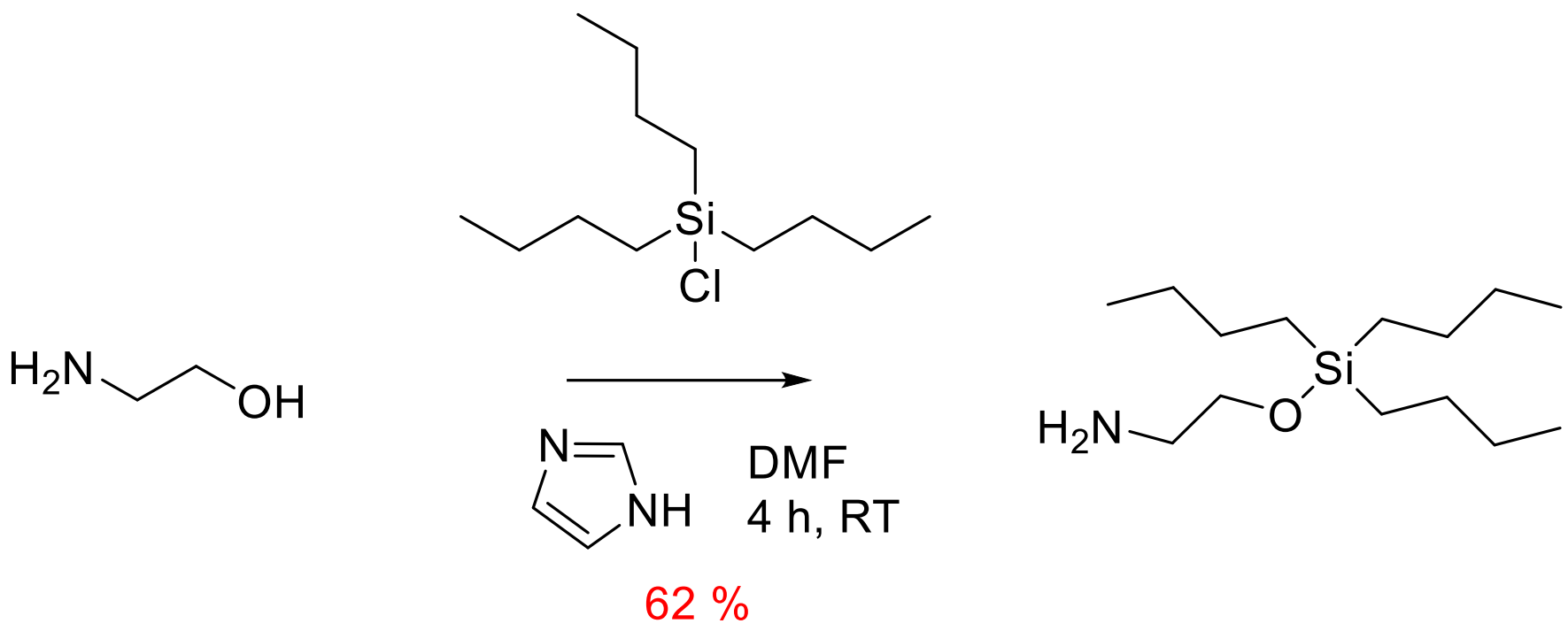

Preparation of 2-((tributylsilyl)oxy)ethan-1-amine

Ethanolamine (0.89 g, 14.5 mmol, 1.1 eq), tributylsilyl chloride (3.10 g, 13.2 mmol, 1eq) and imidazole (1.17g, 17.2 mmol, 1.3 eq) dissolved in anhydrous dimethylformamide (30 mL) under nitrogen. The solution was stirred for 4 h then poured into a separating funnel. The product was extracted with dichloromethane and washed twice with water, once with brine and sodium sulfate was added. This underwent gravity filtration, and the solvent was removed under reduced pressure. The resultant oil was purified on a silica column, eluting with a graduated eluent, from petroleum ether: ethyl acetate (50:50) to (80:20). Removal of solvent from the product fractions gave 2-((tributylsilyl)oxy)ethan-1-amine as a colourless oil (2.13 g, 62%). ^{1}H NMR (400 MHz, $CDCl_3$) δ 3.87 (t, J = 7.0, 2H), 3.39 (t, J = 7.0 Hz, 2H), 1.36-1.29(m, 12H), 0.89 (t, 7.0 Hz, 9H), 0.63-0.59 (m, 6H) ^{13}C NMR (400 MHz, $CDCl_3$) δ 63.37, 33.18, 26.71, 25.45, 13.90, 13.41.

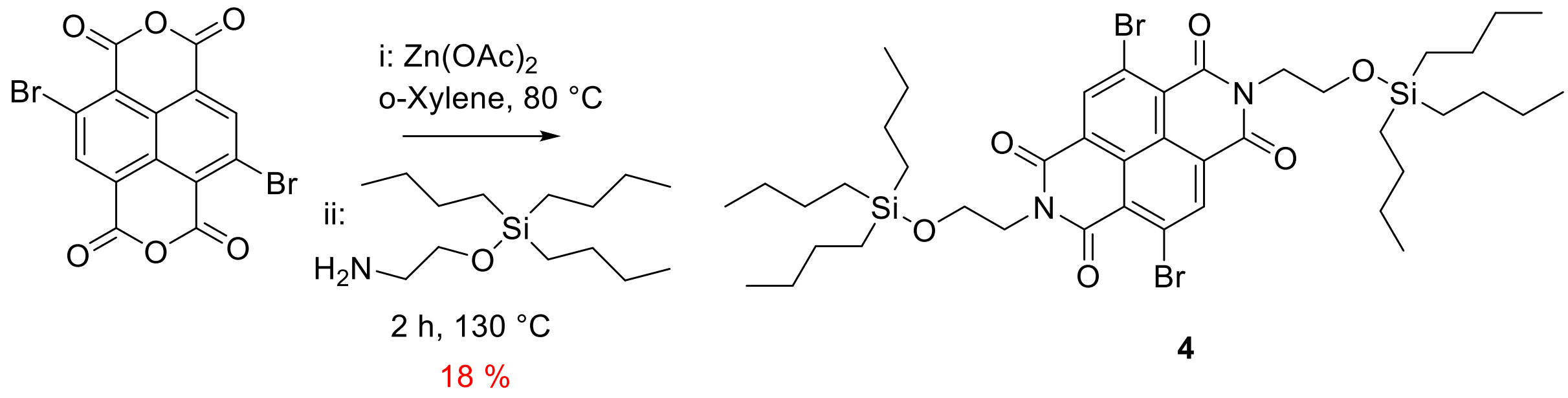

4

Preparation of Compound 4

2,6-Dibromonaphthalene-1,4,5,8-tetracarboxylic dianhydride (0.50g, 1.2 mmol, 1 eq) and anhydrous zinc acetate (0.43g, 2.35 mmol, 2 eq) were added to a 2-neck round bottom flask and put under high vacuum attached to a Schlenk line for 30 minutes, then purged with nitrogen. Anhydrous o-xylene was added and the pale-yellow suspension was stirred at 80 °C under nitrogen. 2-((tributylsilyl)oxy)ethan-1-amine (0.67 g, 2.6 mmol, 2.2 eq) was added dropwise and the temperature was increase to 130 °C. This was allowed to react for 2h, after which time the suspension had become a clear solution and had begun to turn slightly red. It was cooled and poured into a separating funnel with water and extracted with dichloromethane, washing with water, brine, and sodium sulfate was added. This underwent gravity filtration, and the solvent was removed under reduced pressure to give a slightly red oil. This was purified on a silica column, eluting with a graduated eluent, from pure petroleum ether to petroleum ether: ethyl acetate (90:10). The fractions with product were collected and the solvent was removed under reduced pressure. The residue was recrystallised in dichloromethane: methanol overnight and underwent vacuum filtration to recover Compound 4 as fluffy pale-yellow crystals (0.19 g, 18%). 1H NMR (400 MHz, CDCl3) δ 9.00 (s, 2H), 4.41 (t, J = 6.0 Hz, 4H), 3.93 (t, J = 6.0 Hz, 4H), 1.25-1.18 (m, 24 H), 0.79 (t, J = 6.8 Hz, 18H), 0.54-0.50 (m, 12H) ^{13}C NMR (400 MHz, CDCl3) δ 161.00, 160.87, 139.22, 128.52, 127.86, 125.49, 124.23, 59.40, 42.99, 26.67, 25.40, 13.87, 13.34. MS (MALDI-TOF): calcd for $C_{42}H_{64}Br_2N_2O_6Si_2$, 908.27; found 907.90

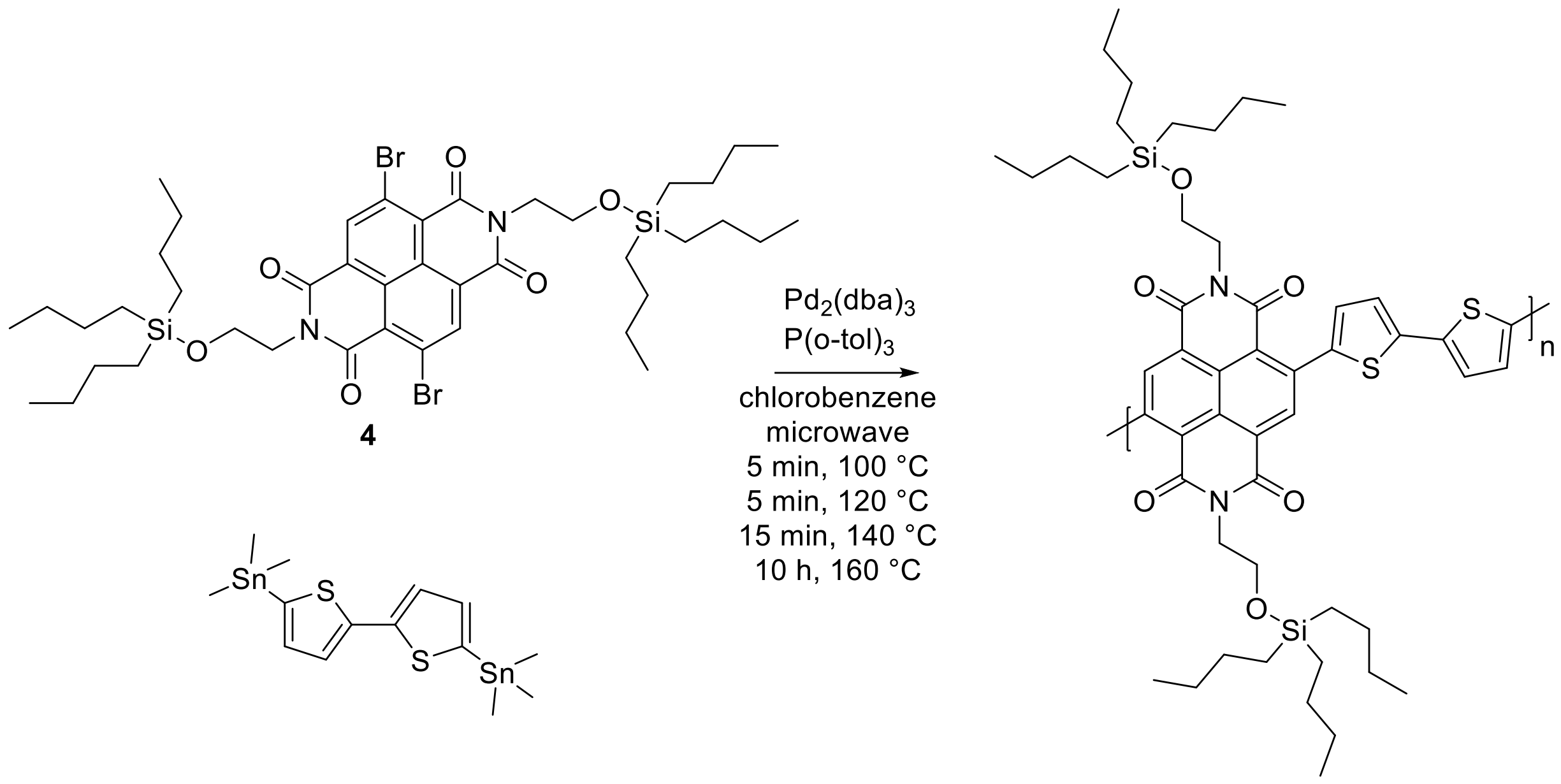

Preparation of P(NDI-T2-2OTBuS)

Compound 4 (100 mg, 0.11 mmol, 1 eq) and 5,5'-bis(trimethylstannyl)-2,2'-bithiophene (54.1 mg, 0.11 mmol, 1 eq) and tri(o-tolyl)phosphine (2.7 mg, 8.8×10^{-3} mmol, 0.08 eq) were weighed directly into a microwave vial, which was then capped with a septum and filled with a nitrogen atmosphere. Chlorobenzene (anhydrous 4.3 mL) was added and the solution was bubbled with dried nitrogen for 20 minutes. The septum was then removed, $Pd_2(dba)_3$ (2.0 mg, 2.2×10^{-3} mmol, 0.02 eq) quickly added and the microwave vial was sealed. After two more minutes of nitrogen bubbling to replace the headspace with nitrogen atmosphere, the solution was heated stepwise in a microwave reactor to 100 °C for 5 min, 120 °C for 5 min, 140 °C for 15 min and finally 160 °C for 10 h. After cooling to room temperature, the reaction solution was diluted with chloroform, precipitated in methanol and filtered through a cellulose Soxhlet thimble. After extraction of oligomers and impurities with methanol, acetone and hexane, the polymer was collected with chloroform, concentrated in vacuo and precipitated in methanol to obtain the polymer as a dark blue solid (86 mg, 83%).

GPC (PhCl): M_n = 10.4 mg mol^{-1}, M_w = 17.9 kg mol^{-1}, Đ = 1.72

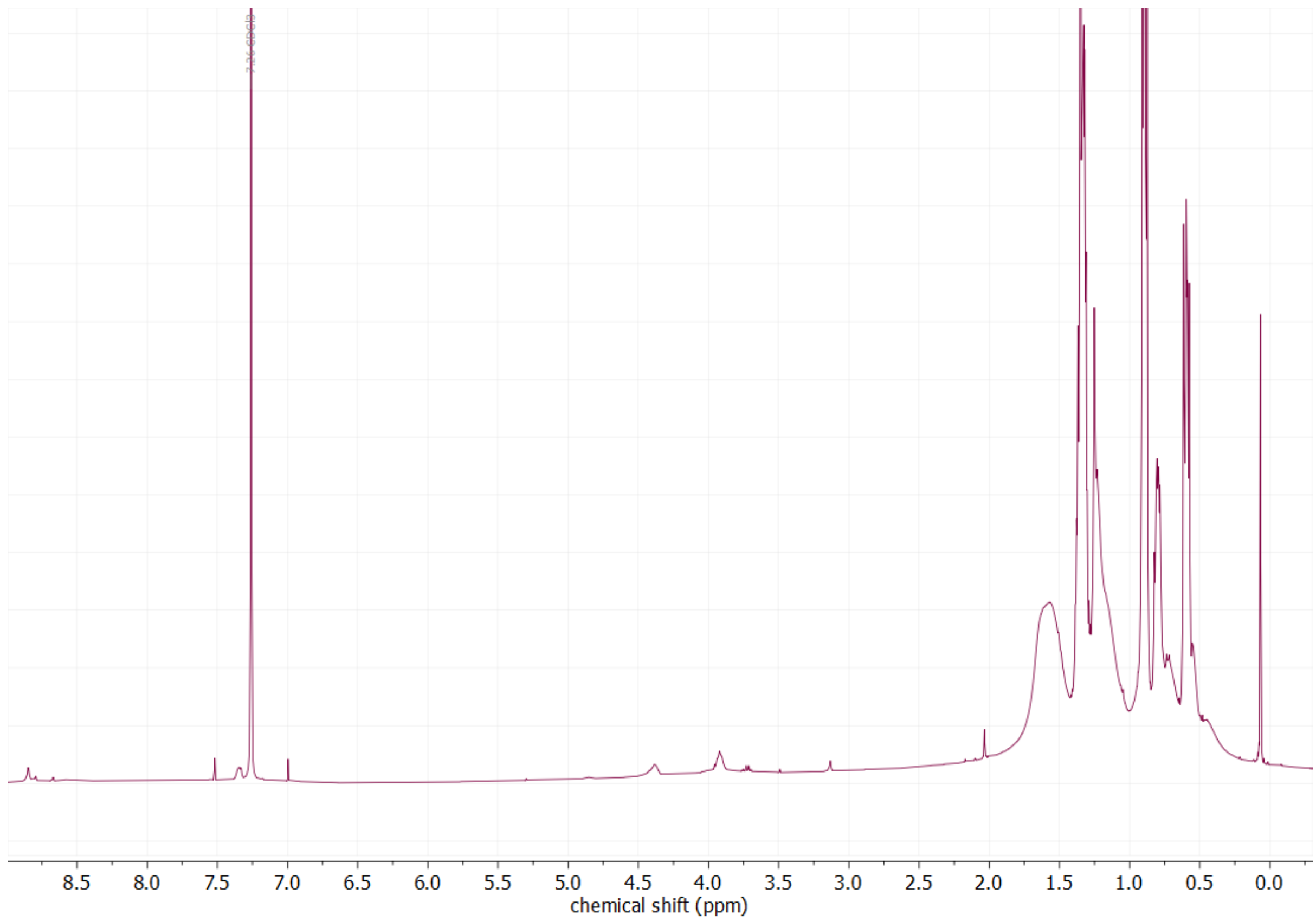

Figure S10. ^{1}H NMR of P(NDI-T2-2OTBuS)

Molecular Weight Averages

Peak	Mp (g/mol)	Mn (g/mol)	Mw (g/mol)	Mz (g/mol)	Mz+1 (g/mol)	Mv (g/mol)
Peak 1	17906	10422	17923	26604	35659	16751

PD
1.72

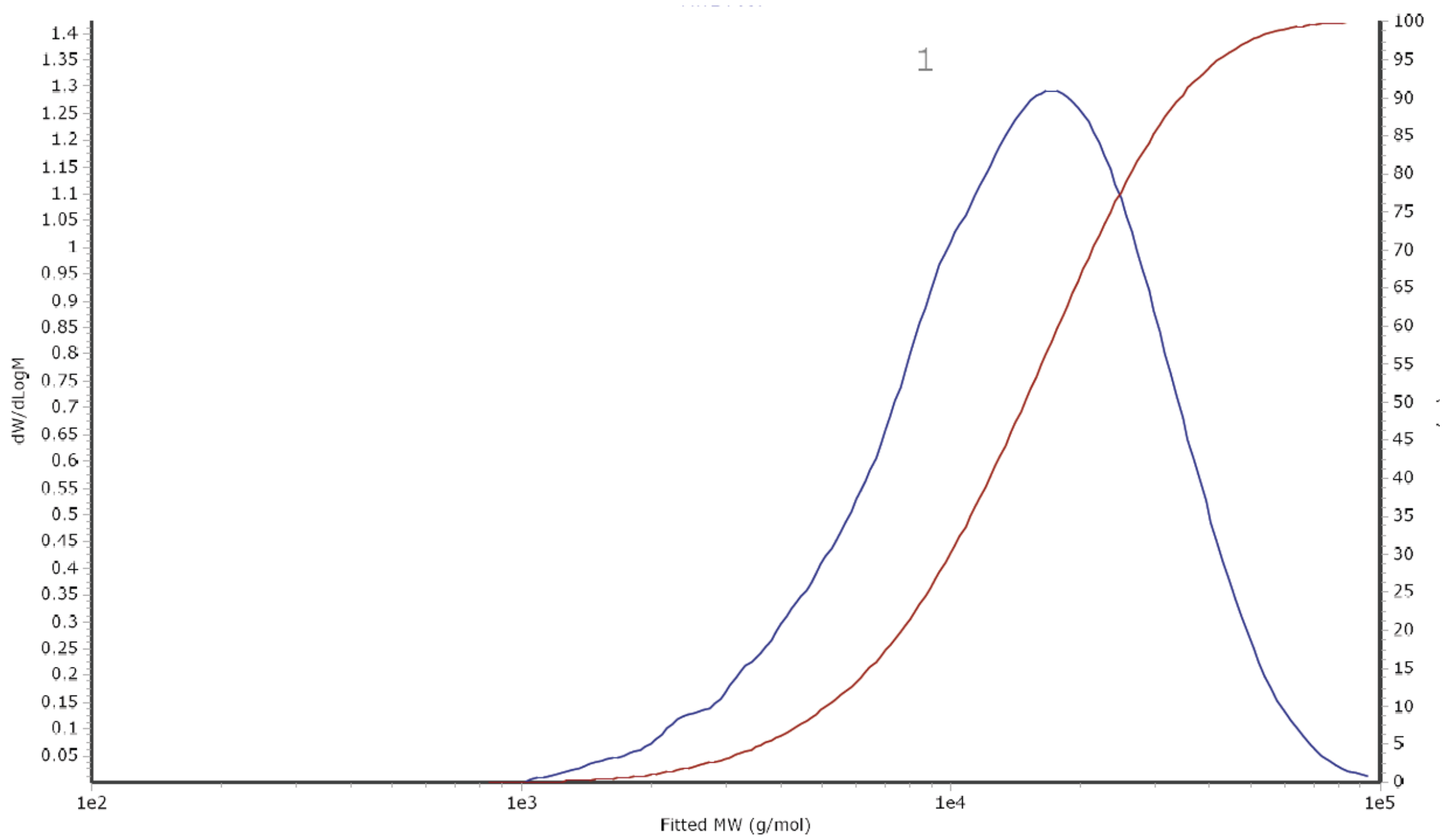

Figure S11. GPC plot of P(NDI-T2-2OTBuS)

Section 3 Optoelectronic properties of the conjugated polymers

This section presents the frontier energy levels of the conjugated copolymers, before and after deprotection.. The HOMO was determined by photoemission spectroscopy in air (PESA, Figure S12) and the optical band gap of each sample was extracted from the onset energy in UV–Vis spectroscopy (Figure S13). The LUMO levels were subsequently derived from the PESA-measured HOMO values combined with the optical band gap.

3.1 Photoemission Spectroscopy in Air (PESA)

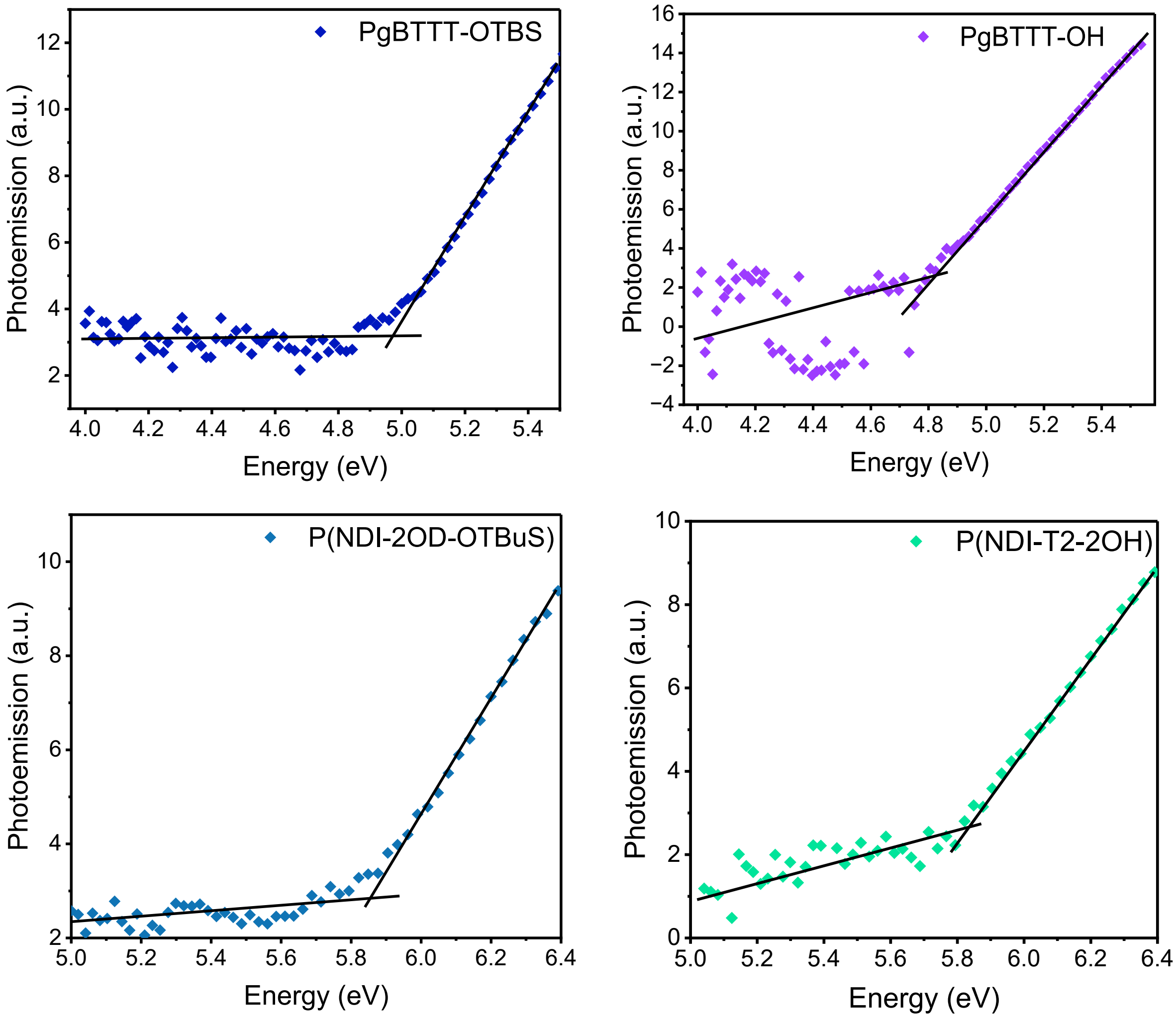

Figure S12 PESA diagram of (a) PgBTTT-OTBS, (b) PgBTTT-OH, (c) P(NDI-T2-2OTBuS), (d) P(NDI-T2-2OH). The Cube root of photoemission quantum yield was plotted against photon energy and fitted linearly, and the HOMO level was determined from the intersection of the fit

3.2 UV-Vis Spectroscopy

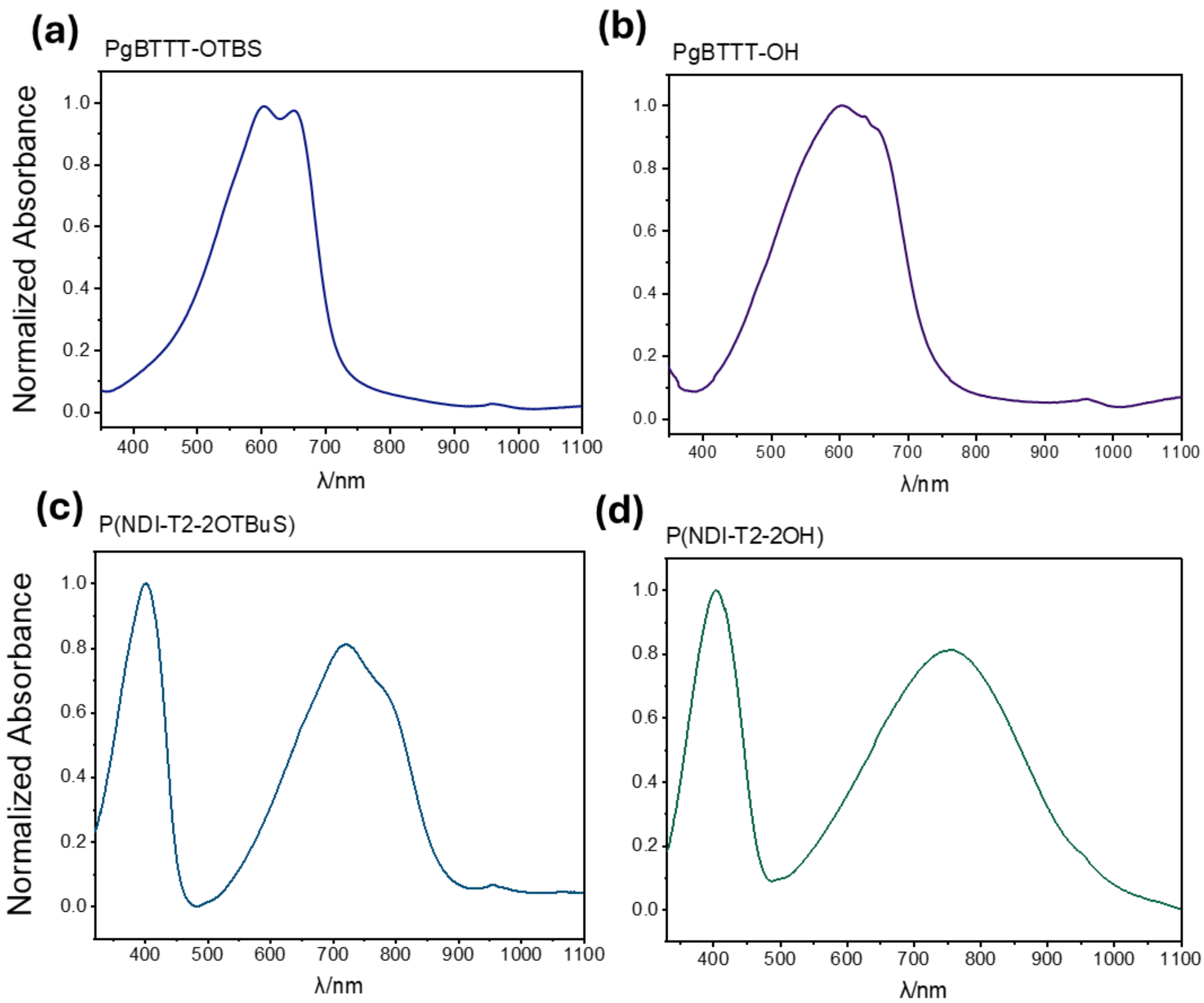

Figure S13 UV-Vis Spectroscopy plots of (a) PgBTTT-OTBS, (b) PgBTTT-OH, (c) P(NDI-T2-2OTBuS) and (d) P(NDI-T2-2OH). The polymer films are deposited on cut glass substrates.

3.3 Scan-rate dependent cyclic voltammetry of PgBTTT-OTBS/PgBTTT-OH

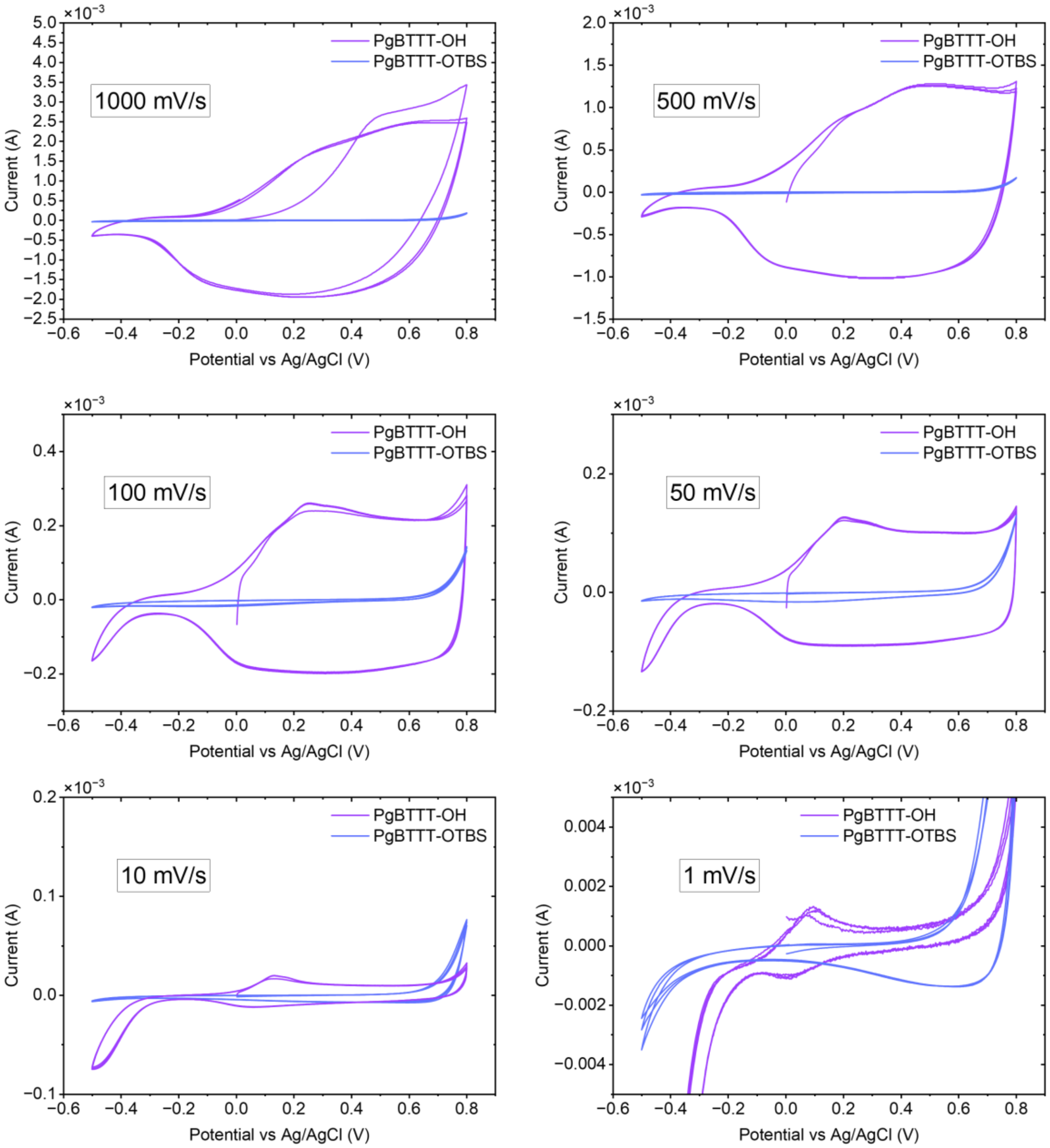

Figure S14 Cyclic voltammograms of P(gBTTT-OTBS) recorded in 0.1 M aqueous NaCl across scan rates ranging from 1 to 1000 mV/s.

Section 4 Atomic Force Microscopy

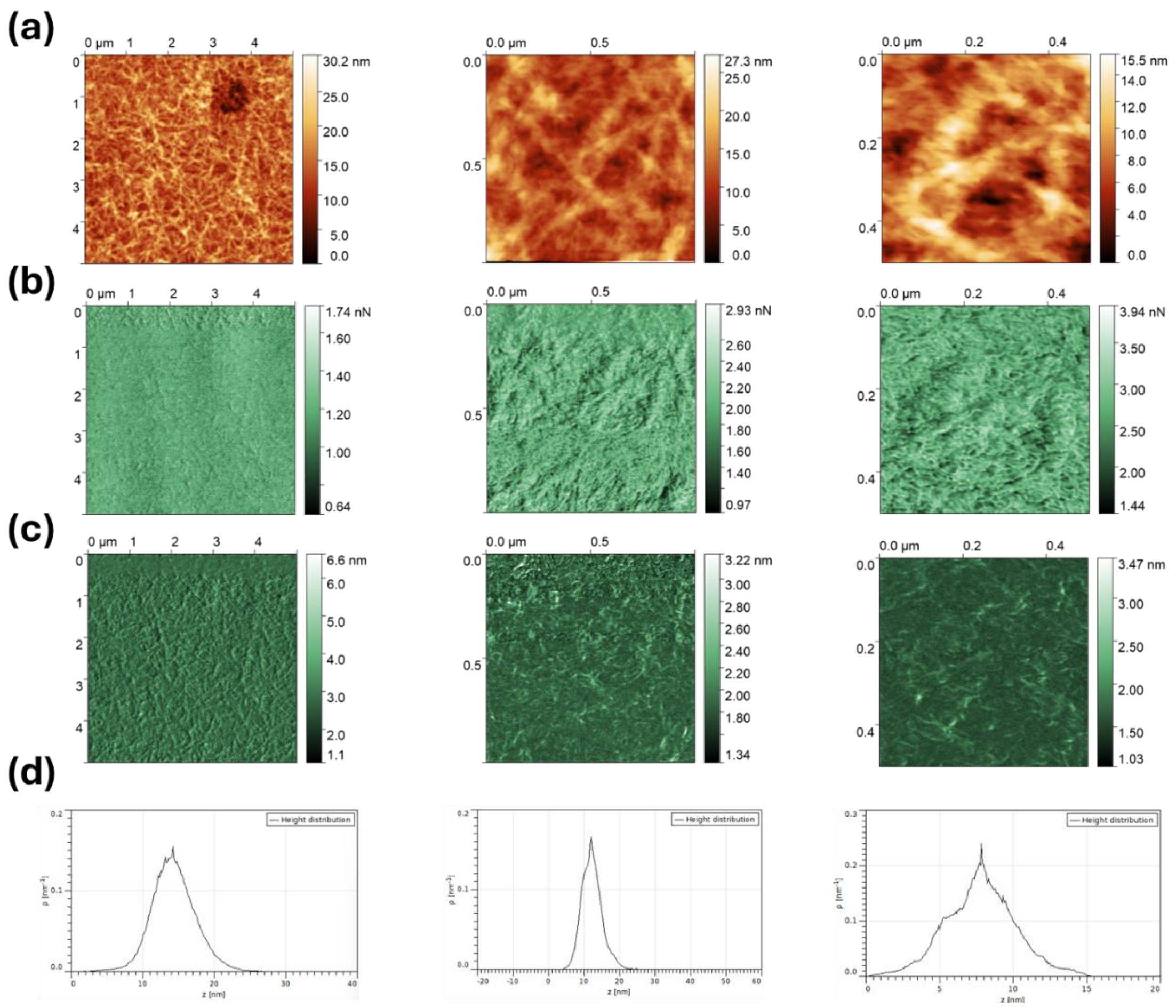

Figure S15 From left to right, the image was zoomed in to a 5μm x 5μm, 1μm x 1 μm and 500 nm x 500 nm square on the polymer film of PgBTTT-OTBS, with RMS roughness of 3.06, 3.17 and 2.38 nm respectively. Subfigure (a), (b) and (c) shows height, adhesion and deformation profile of imaged by Atomic Force Microscopy, respectively. Subfigure (d) shows the height distribution of the polymer film.

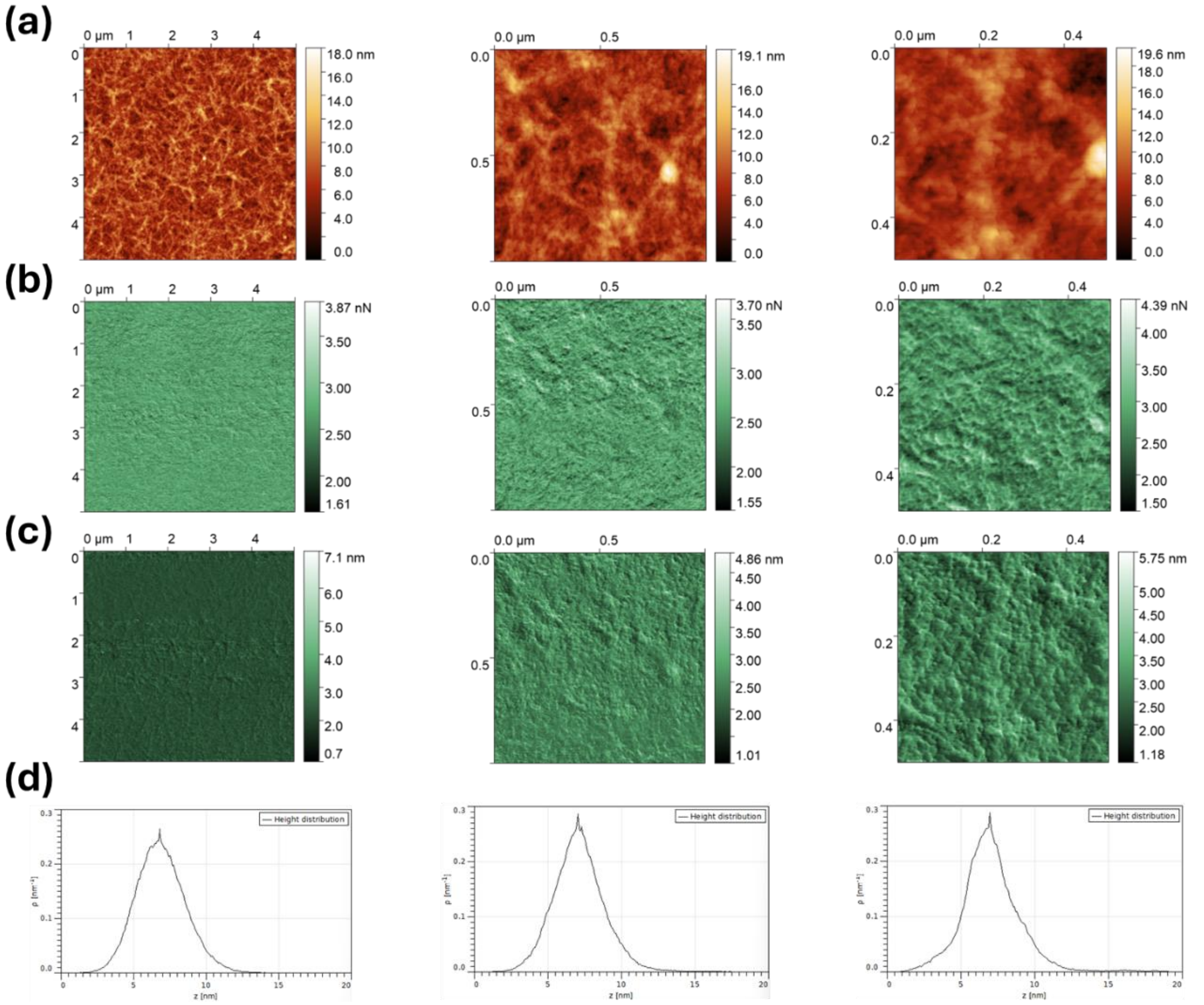

Figure S16 From left to right, the image was zoomed in to a 5µm x 5µm, 1µm x 1 µm and 500 nm x 500 nm square on the polymer film of PgBTTT-OH, with RMS roughness of 1.70, 1.80 and 1.70 nm respectively. Subfigure (a), (b) and (c) shows height, adhesion and deformation profile imaged by Atomic Force Microscopy, respectively. Subfigure (d) shows the height distribution of the polymer film.

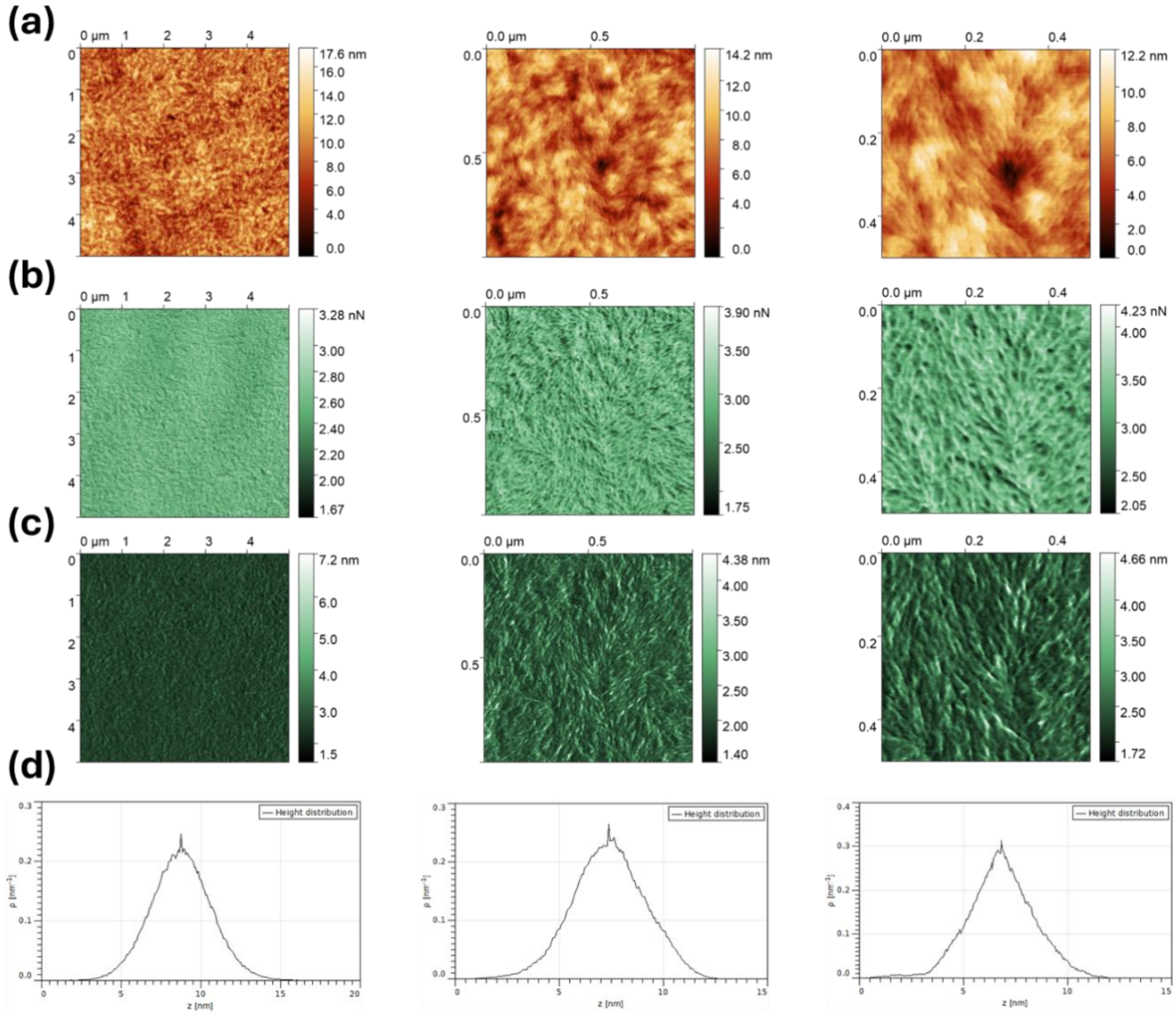

Figure S17 From left to right, the image was zoomed in to a 5μm x 5μm, 1μm x 1 μm and 500 nm x 500 nm square on the polymer film of P(NDI-T2-2OTBuS), with RMS roughness of 1.89, 1.75 and 1.60 nm respectively. Subfigure (a), (b) and (c) shows height, adhesion and deformation profile imaged by Atomic Force Microscopy, respectively. Subfigure (d) shows the height distribution of the polymer film.

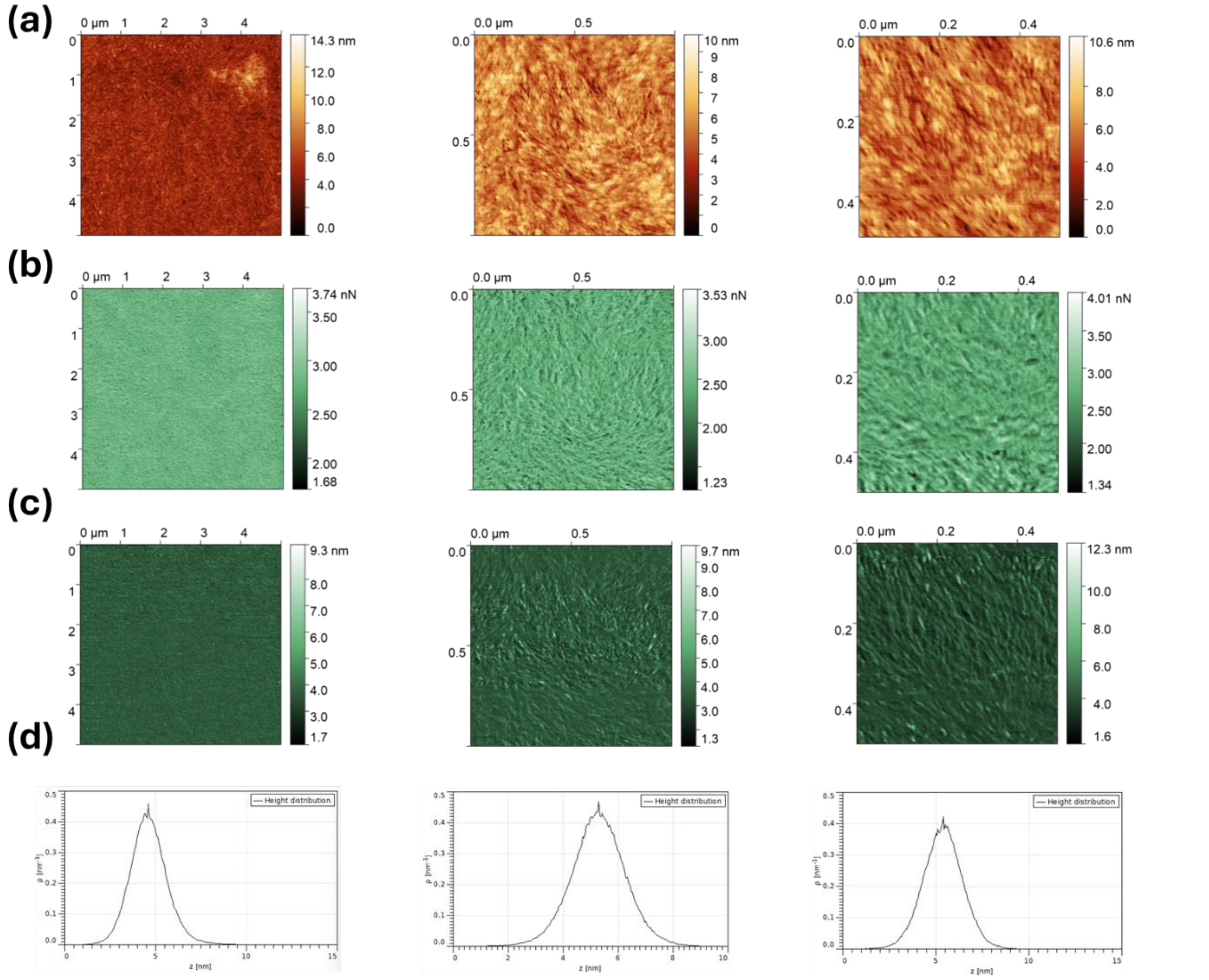

Figure S18 From left to right, the image was zoomed in to a 5μm x 5μm, 1μm x 1 μm and 500 nm x 500 nm square on the polymer film of P(NDI-T2-2OH), with RMS roughness of 1.03, 0.99 and 1.06 nm respectively. Subfigure (a), (b) and (c) shows height, adhesion and deformation profile imaged by Atomic Force Microscopy, respectively. Subfigure (d) shows the height distribution of the polymer film.

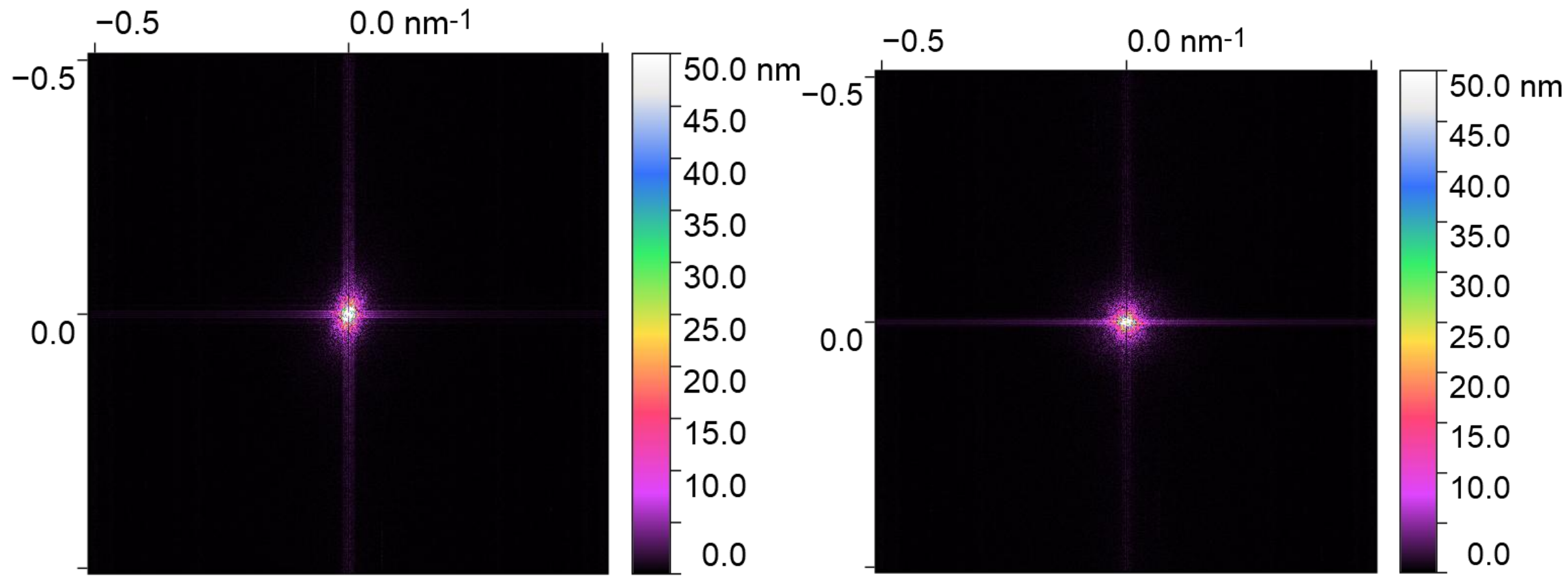

Figure S19 Fast fourier transform analysis of the AFM height image (500 nm x 500 nm) of PgBTTT-OTBS and PgBTTT-OH).

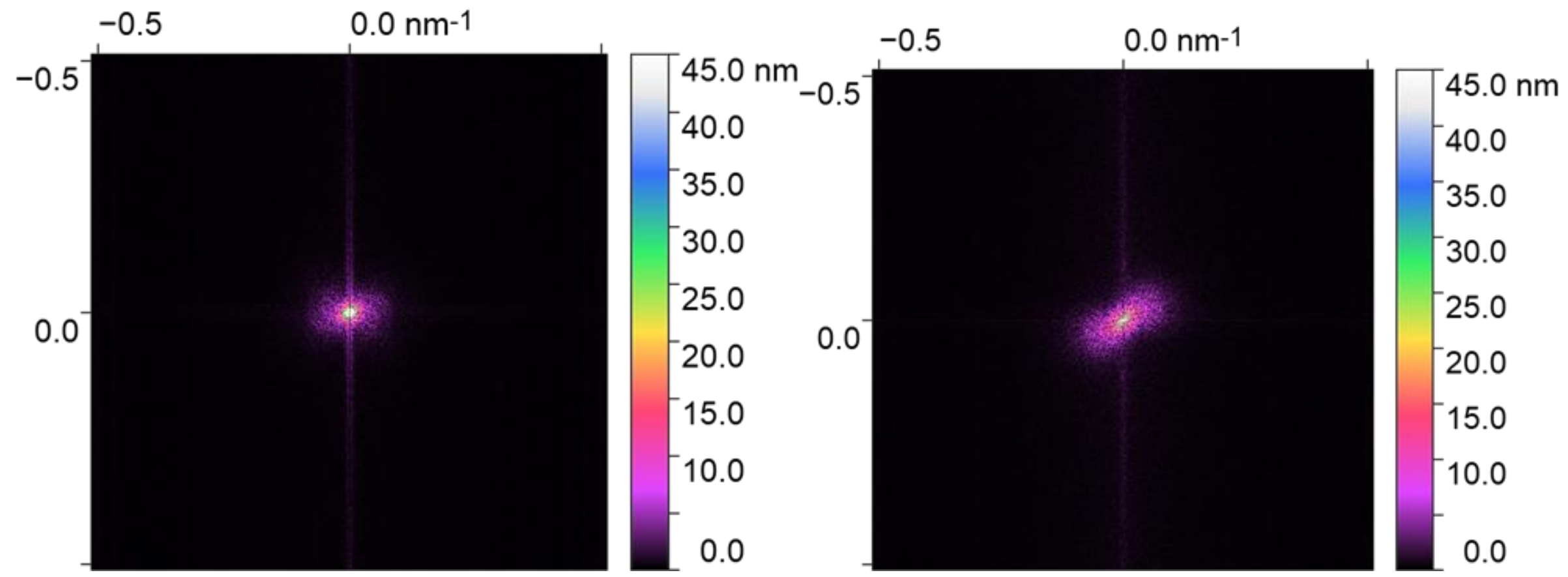

Figure S20 Fast fourier transform analysis of the AFM height image (500 nm x 500 nm) of P(NDI-T2-2OTBuS) and P(NDI-T2-2OH).

Section 5 Organic Electrochemical Transistor Characterization

5.1 Experimental Section

All devices were fabricated on 4-inch borosilicate glass wafers, 700 µm-thick and double-side polished (Microchemicals), which were sequentially cleaned by sonication in 2% Decon 90, deionized water, acetone and isopropanol for 10 min each. The wafers were dehydrated at 150 ºC for 5 min and then cooled to room temperature for 10 min. AZ nLOF 2035 photoresist was spin-coated at 400 rpm for 5 s with an acceleration of 1000 rpm/s, followed by 3000 rpm for 45 s with an acceleration 8000 rpms/s. The resist was soft-baked at 110 ºC for 60 s, exposed to UV light with a dose of 90 mJ/cm^2 in vacuum contact mode using a MA/BA6 mask aligner, (Karl Suss), post-exposure baked at 110 ºC for 60 s, and developed in AZ 726 MIF (Microchemicals) for 20 s.

The patterned wafer was coated with 10 nm titanium and 100 nm gold by electron-beam evaporator (LEV Kurt J Lesker), followed by lift-off in acetone overnight and rinsing with isopropanol. The metal-patterned wafers were then coated with a bilayer parylene-C stack. Prior to parylene deposition, the wafers were treated with O_2 plasma for 60 s and immersed for 45 s in a silane solution containing 3 vol% A174 silane (Sigma-Aldrich) in 1 vol% acetic acid in 99.8% ethanol and thermally treated at 70 ºC for 1 h. A 1µm parylene-C layer was deposited using a parylene coater (PDS 2010 LABCOATER, Specialty Coating Systems). Subsequently, two layers of soap solution, 2 vol% Micro 90 in DI water, were spin-coated at 1000 rpm for 2 min with an acceleration of 100 rpm/s. The wafers were dried at ambient environment for 2 min, followed by deposition of a 2-µm parylene-C layer.

The bilayer parylene-C stack was patterned by photolithography using AZ 10XT (520 cP, Microchemicals) photoresist. The photoresist was spin coated at 3000 rpm for 45s with an acceleration of 8000 rpm/s and soft-baked at 110 ºC for 2 min. The resist was exposed with a

dose of 540 mJ/cm^2 and developed in AZ 400K diluted 1:3 v/v in DI water for 5 min. The parylene-C was then etched by reactive ion etching. The remaining photoresist was removed with dichloromethane for 1 min. Finally, the conducting polymer was spin-coated onto the bilayer parylene-C devices and patterned by peeling off the top parylene-C using kapton tape.

OECTs were characterized using a Keysight B2902A Source-Measure Unit controlled custom Python code.

5.2 Transfer Curve of PgBTTT-OTBS

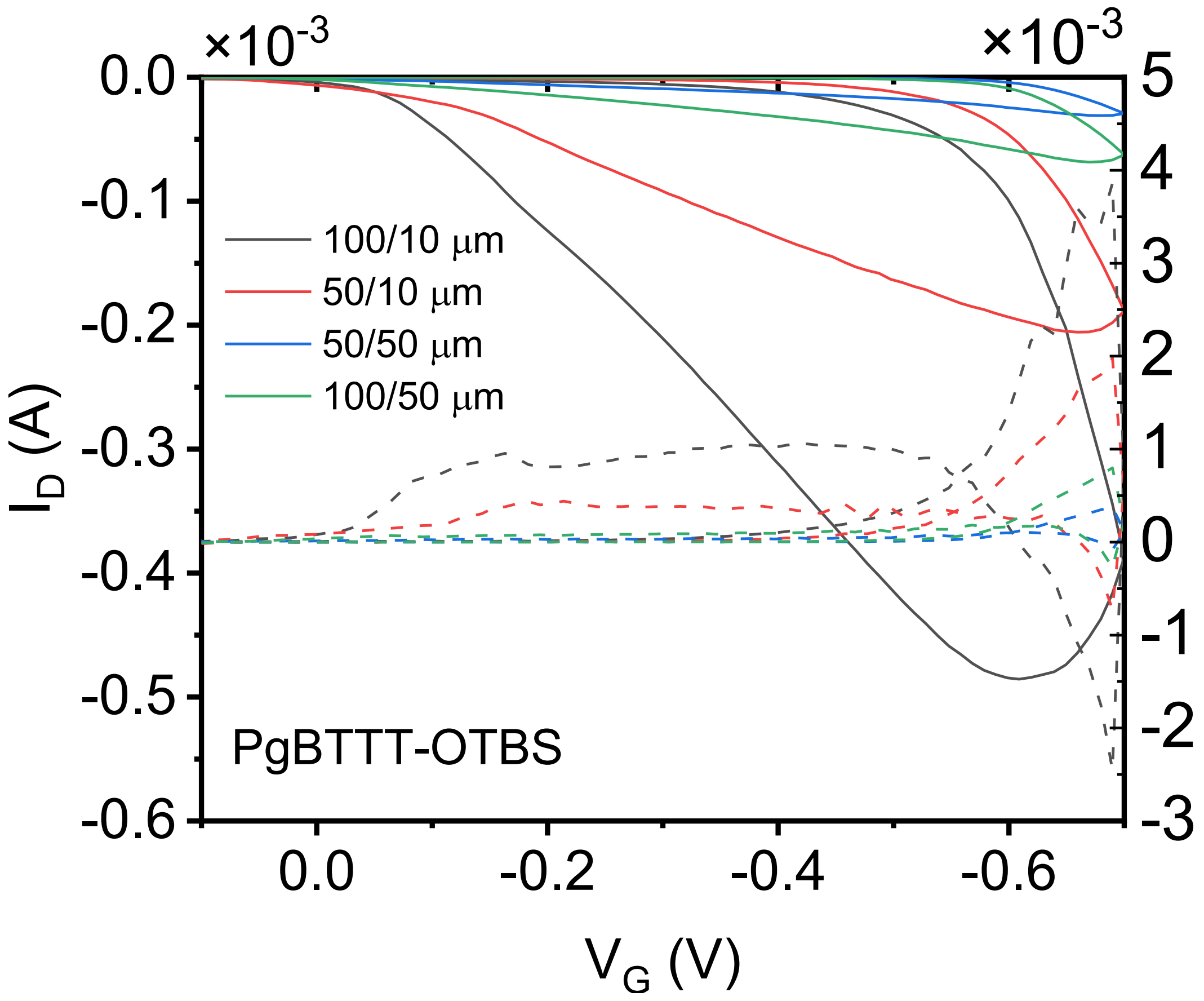

Figure S21 geometry-dependent I_{DS} and transconductance of PgBTTT-OTBS under a sweep rate of 10 mV/s

5.3 Figure-of-merit Evaluation

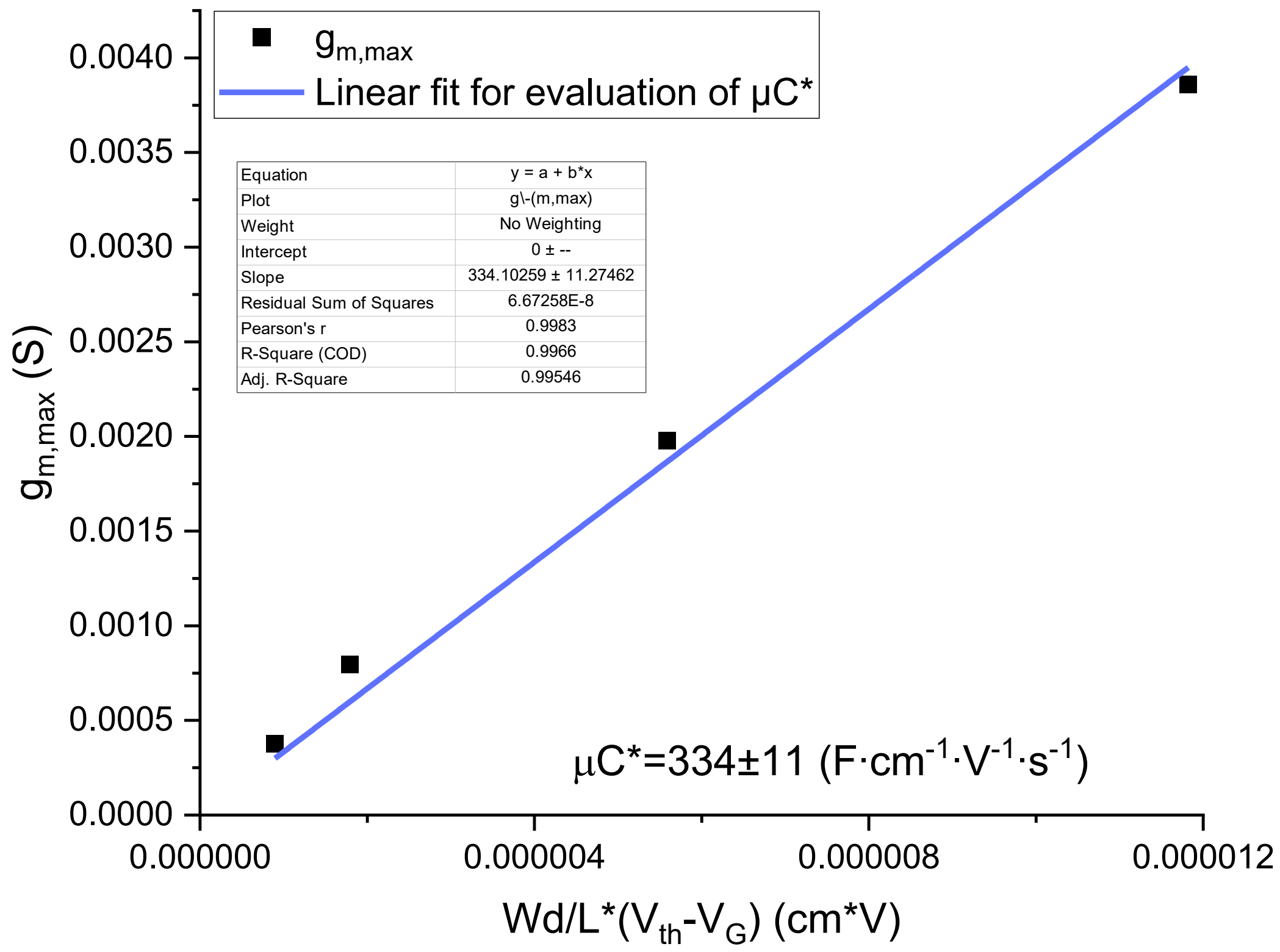

Figure S22 Transconductance of PgBTTT-OTBS (gm) as a function of geometry of the channels. The points represent measurements from device with channel dimensions (W/L) of 100µm/10µm, 50µm/10µm, 100µm/50µm and 50µm/50µm. In accordance with the Bernard-Malliaras model, the data were fitted without a constant term in the y-intercept.

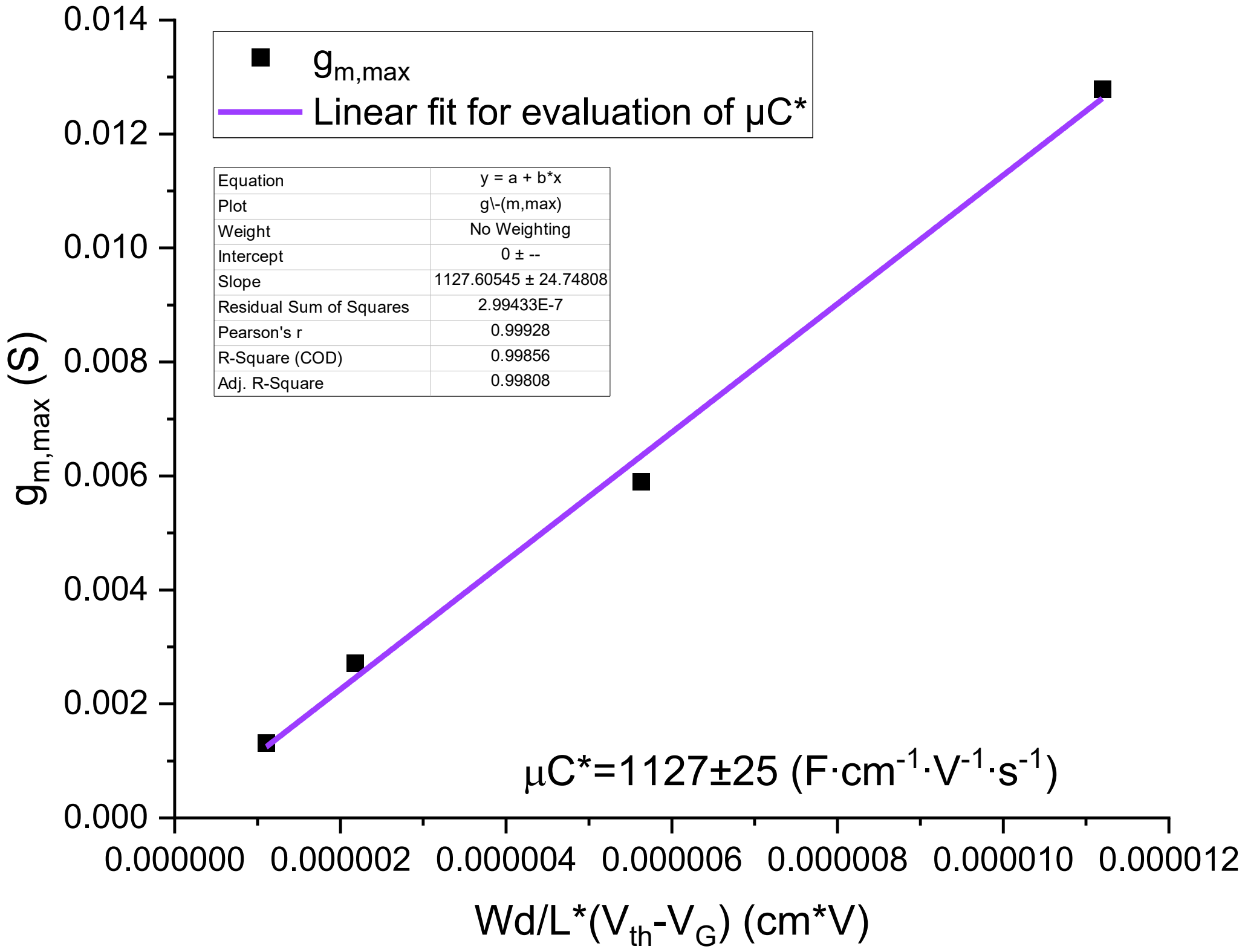

Figure S23 Transconductance of PgBTTT-OH (gm) as a function of geometry of the channels. The points represent measurements from device with channel dimensions (W/L) of 100µm/10µm, 50µm/10µm, 100µm/50µm and 50µm/50µm. In accordance with the Bernard-Malliaras model, the data were fitted without a constant term in the y-intercept.

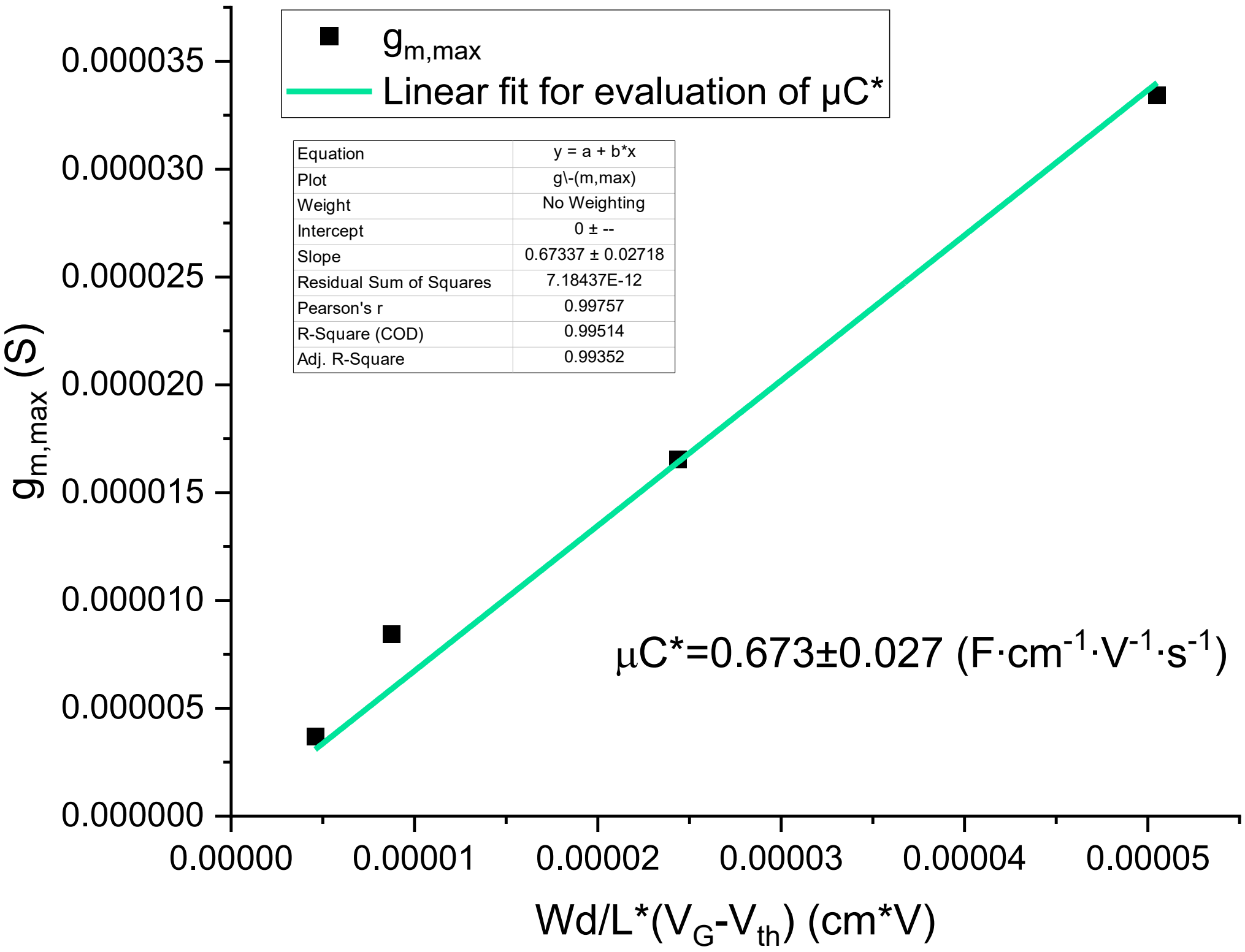

Figure S24 Transconductance of P(NDI-T2-2OH) (gm) as a function of geometry of the channels. The points represent measurements from device with channel dimensions (W/L) of 100µm/10µm, 50µm/10µm, 100µm/50µm and 50µm/50µm. In accordance with the Bernard-Malliaras model, the data were fitted without a constant term in the y-intercept.

5.4 Electrochemical Impedance Spectroscopy

EIS measurements were carried out using a Metrohm Germany PGSTAT302N analyser module and a NOVA 2.1 software package for analysis. For the measurement, a rubber gasket with a circular aperture of 1 cm diameter was placed on top of the OECT substrates. The cavity was filled with 1.5 mL of previously degassed aqueous 0.1 M NaCl solution and allowed to settle for 5 min. The polymer was structured via a sacrificial Parylene C layer on an underlying planar square electrode of the identical area (0.5 × 0.5 mm), which was used as the working electrode and the sense line of the potentiostat. An aqueous Ag/AgCl reference electrode and platinum wire counter electrode were dipped into the electrolyte solution. The impedance was recorded as a function of redox potential up to ± 0.8 V versus Ag/AgCl (aq) in a frequency range of 1 to 100 kHz in discrete potential steps with an equilibration time of 5 s between each step. The system was excited with 10 mV RMS sinusoidal perturbation and measured with a resolution of 10 frequency steps per decade.

The capacitance as a function of frequency f was determined from the measured complex impedance Z^* data using the following expression:

$$C(f) = \frac{1}{j\,2\,\pi f\,Z^*(f)}$$

where j is the imaginary unit. This allowed for the extraction of the real and imaginary capacitance components across the scanned frequency range. The capacitance at a frequency of 1 Hz was normalized by the film volume (0.0025 cm^2 × d) to obtain the volumetric capacitance at the respective redox potential.

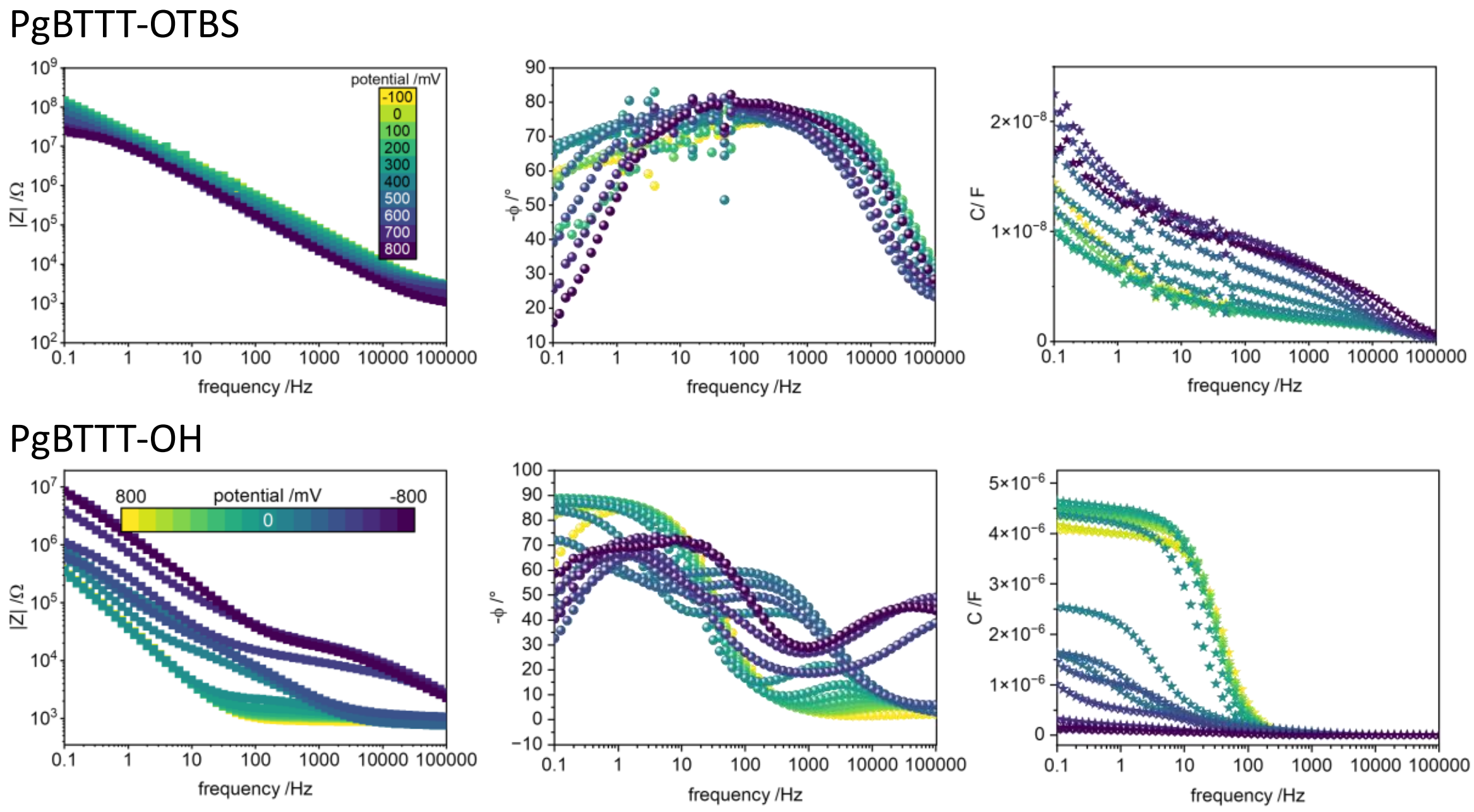

Figure S25 the magnitude and phase of the impedance of (top) PgBTTT-OTBS and (bottom) PgBTTT-OH, at various applied V_G, alongside the equivalent circuit fit (Randle circuit).

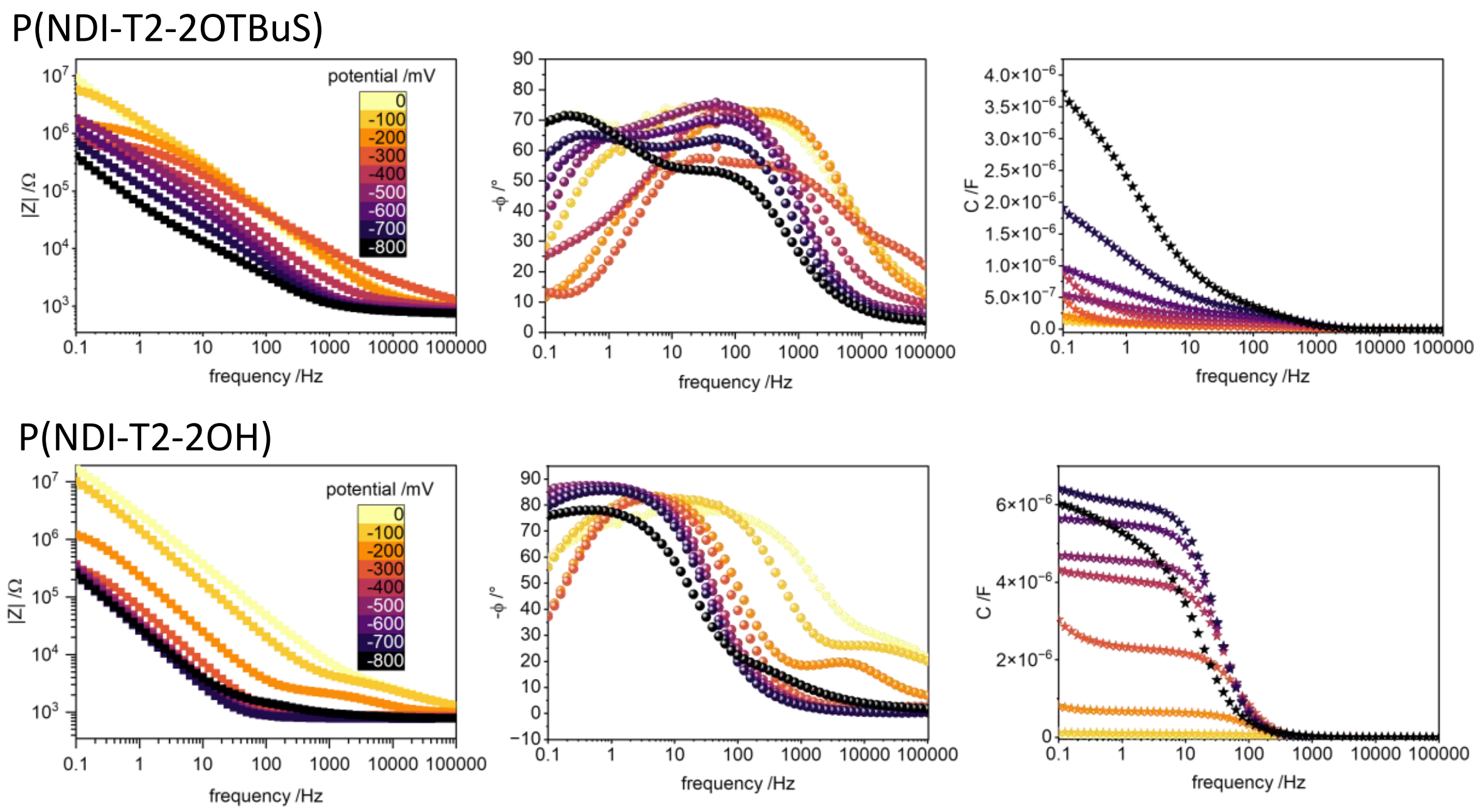

Figure S26 the magnitude and phase of the impedance of (top) P(NDI-T2-2OTBuS) and (bottom) P(NDI-T2-2OH), various applied V_G, alongside the equivalent circuit fit (Randle circuit).

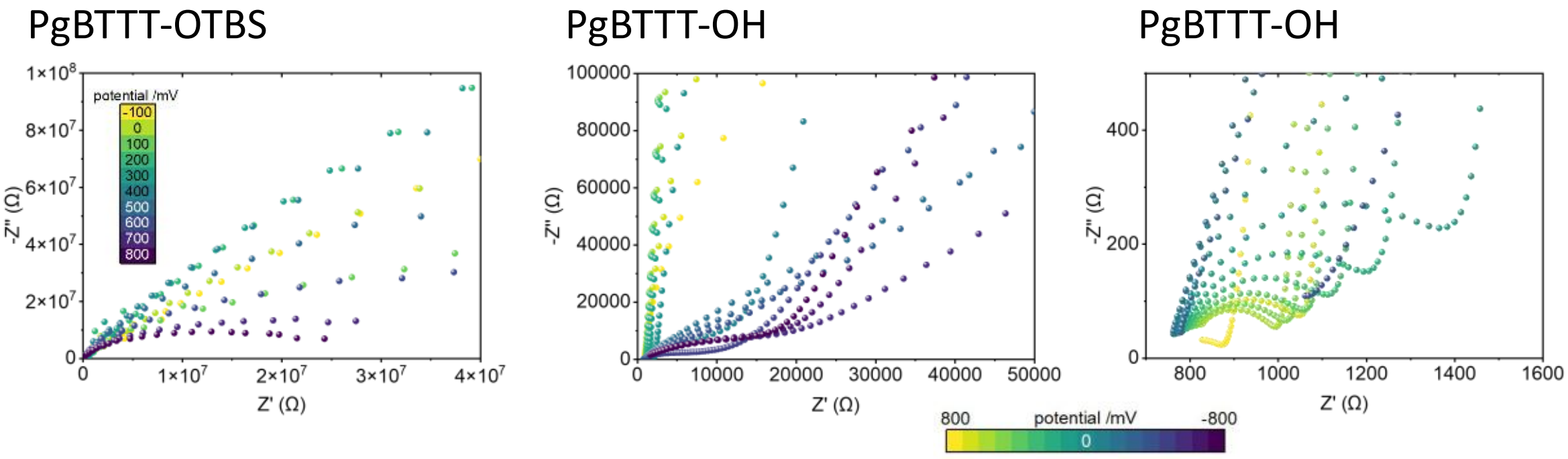

Figure S27 Nyquist plot of PgBTTT-OTBS and PgBTTT-OH. The PgBTTT-OH plot is magnified to highlight features in the low Z' and -Z'' range.

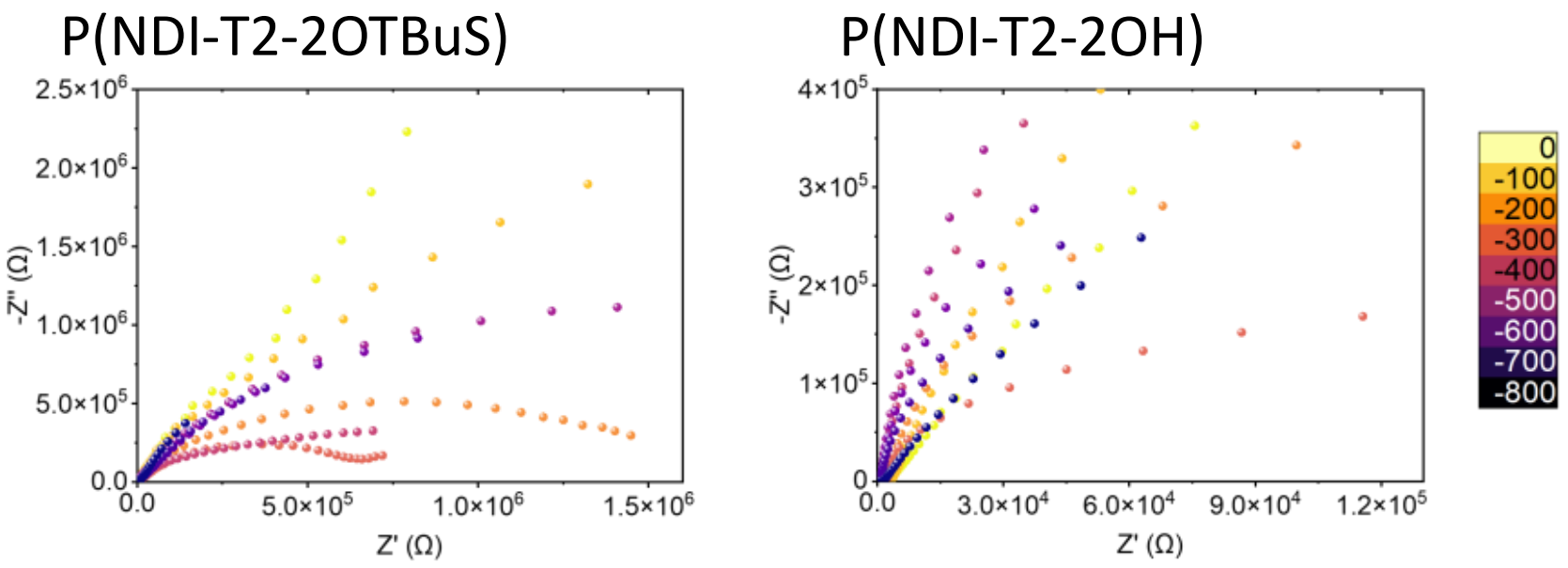

Figure S28 Nyquist plot of P(NDI-T2-2OTBuS) and P(NDI-T2-2OH)

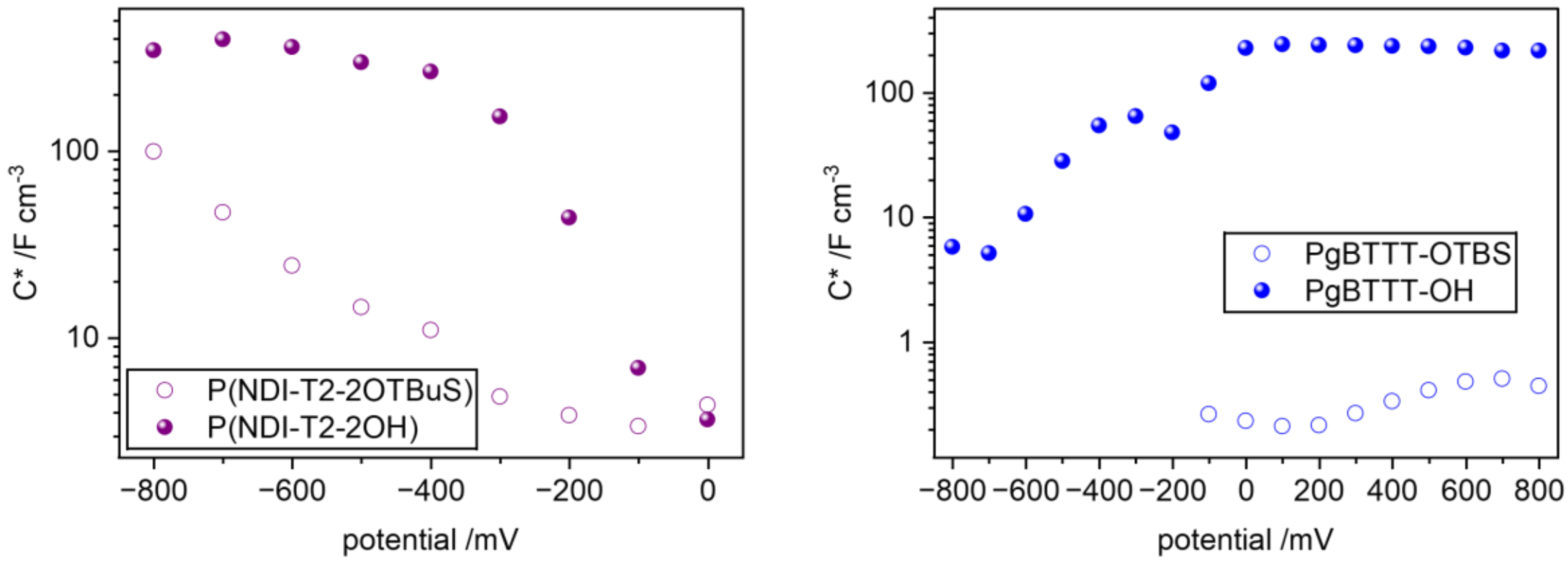

Figure S29 Plot of C as a function of applied VG. All values were determined at 1 Hz. The capacitance of PgBTTT-OTBS and P(NDI-T2-2OTBuS) did not reach saturation within the accessible frequency window and are included here for comparison with PgBTTT-OH and P(NDI-T2-2OH), respectively.*

Table S1. Maximum volumetric capacitance and the respective redox potential of the silyl-ether polymers before and after cleavage, as well as the volumetric capacitance at the potential of maximum transconductance and the respective voltage.

	**P(NDI-T2-2OH)**	**PgBTTT-OH**
**C* (max) /F cm^{-3}**	396.69	245.40
**voltage /mV**	-700	100
**C* @ $g_{m, max}$ /F cm^{-3}**	360.99	218.53
**voltage /mV**	-600	-700

5.5 Transient response measurement

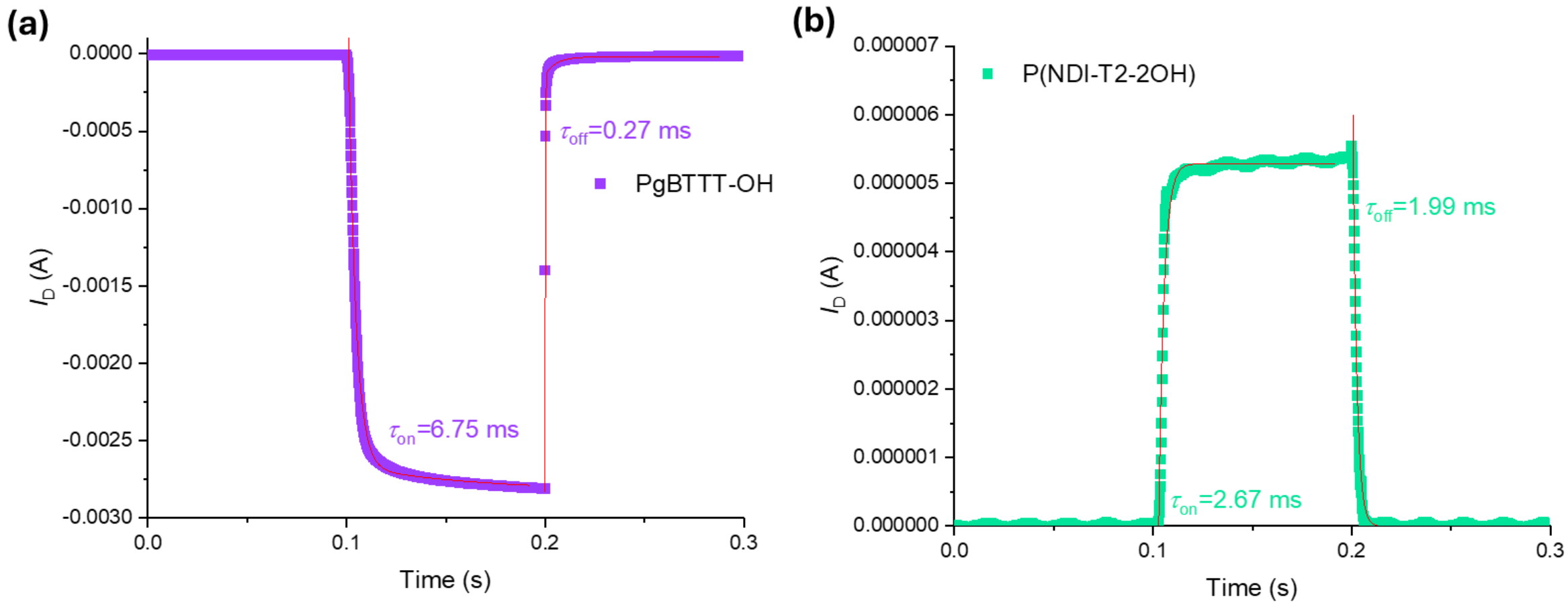

Figure S30 Transient response of (a) PgBTTT-OH under a gate voltage pulse of -0.6 V for 0.1 s and (b) P(NDI-T2-2OH) under a gate voltage pulse of 0.6 V for 0.1 s

5.6 Stability measurement

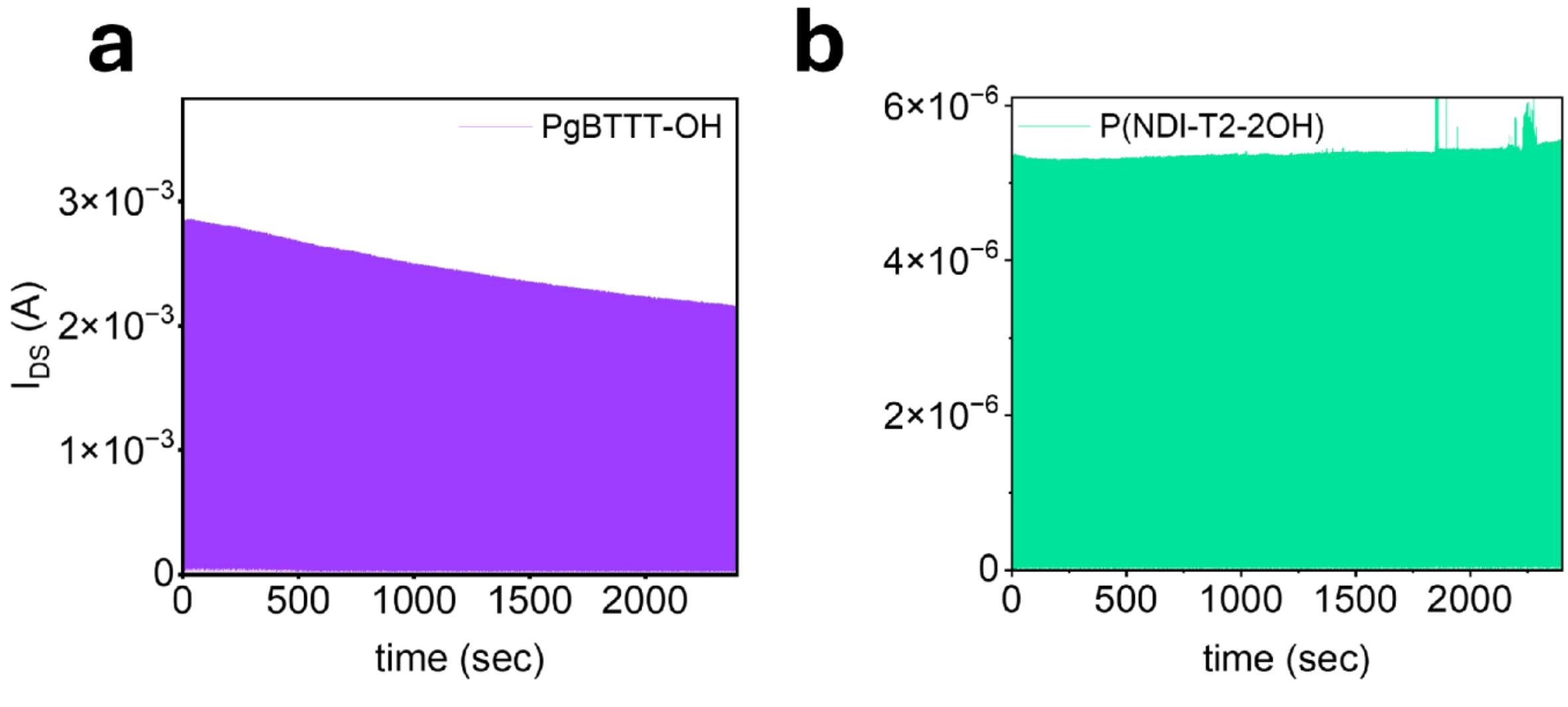

Figure S31 Raw stability profile of (a) PgBTTT-OH and (b) P(NDI-T2-2OH)

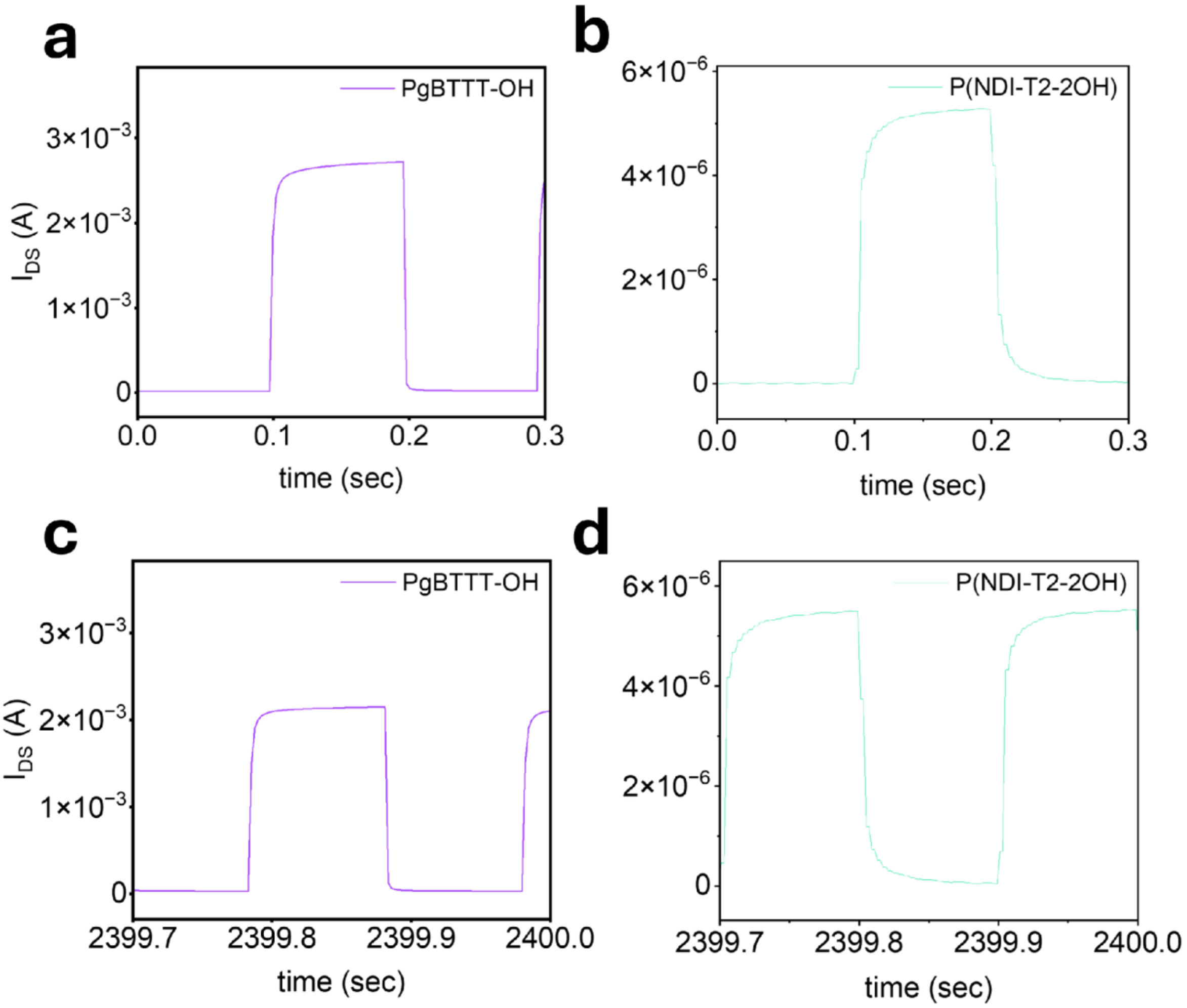

Figure S32 Magnified stability profiles of (a) PgBTTT-OH and (b) P(NDI-T2-2OH) from 0 to 0.3 s, and (c) PgBTTT-OH and (d) P(NDI-T2-2OH) from 2399.7 to 2400.0 s.

Section 6 Grazing Incidence Wide Angle X-ray Scattering (GIWAXS)

6.1 Summary table of GIWAXS scattering peaks

Table S2 Summary of fitted lamellar and π-π stacking q-distance, d-spacing and coherent length of all the conjugated polymers

	Lamellar peak (100)				π-π stacking			
Sample	q_{100} [A^{-1}]	$FWHM_{100}$ [A^{-1}]	d_{100} [A]	Lc_{100} [A]	q_{π} [A^{-1}]	$FWHM_{\pi}$ [A^{-1}]	d_{π} [A]	Lc_{π} [A]
PgBTTT-OTBS	0.31	0.0500	20.3	113	1.77	0.145	3.55	39.0
PgBTTT-OH	0.48	0.0578	13.2	97.8	1.81	0.120	3.47	47.1
P(NDI-T2-2OTBuS)	0.28	0.0822	22.1	68.8	1.69	0.208	3.72	27.3
P(NDI-T2-2OH)	0.51	0.162	12.5	45.3	1.74	0.698	3.59	8.1

6.2 Azimuth dependence of scattering profile

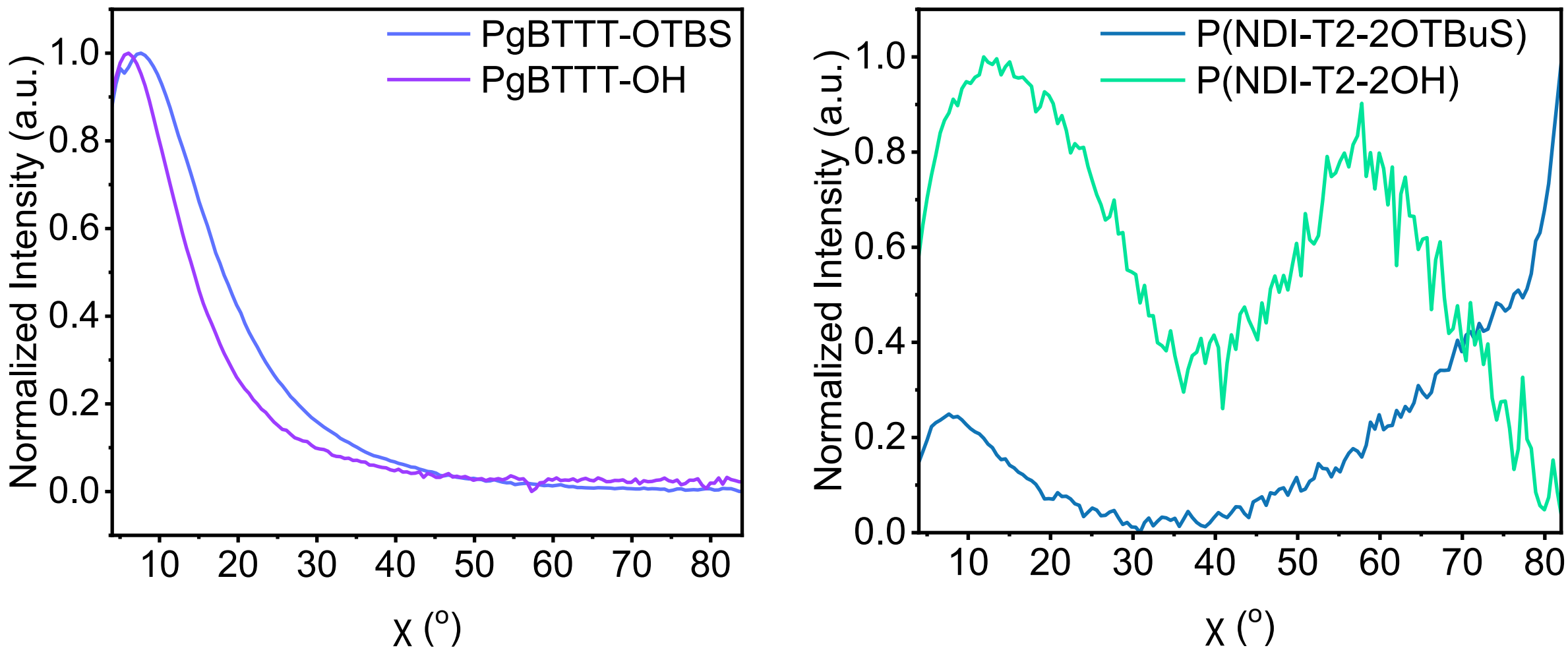

Figure S33 Plot of normalized total intensity of scattering in 2D GIWAXS the azimuthal angle χ, which is defined with respect to the q_z axis. χ = 0° is the out-of-plane (q_z) axis, while χ = 90° is the in-plane (q_{xy}) axis.

Section 7 X-ray Photoelectron Spectroscopy (XPS)

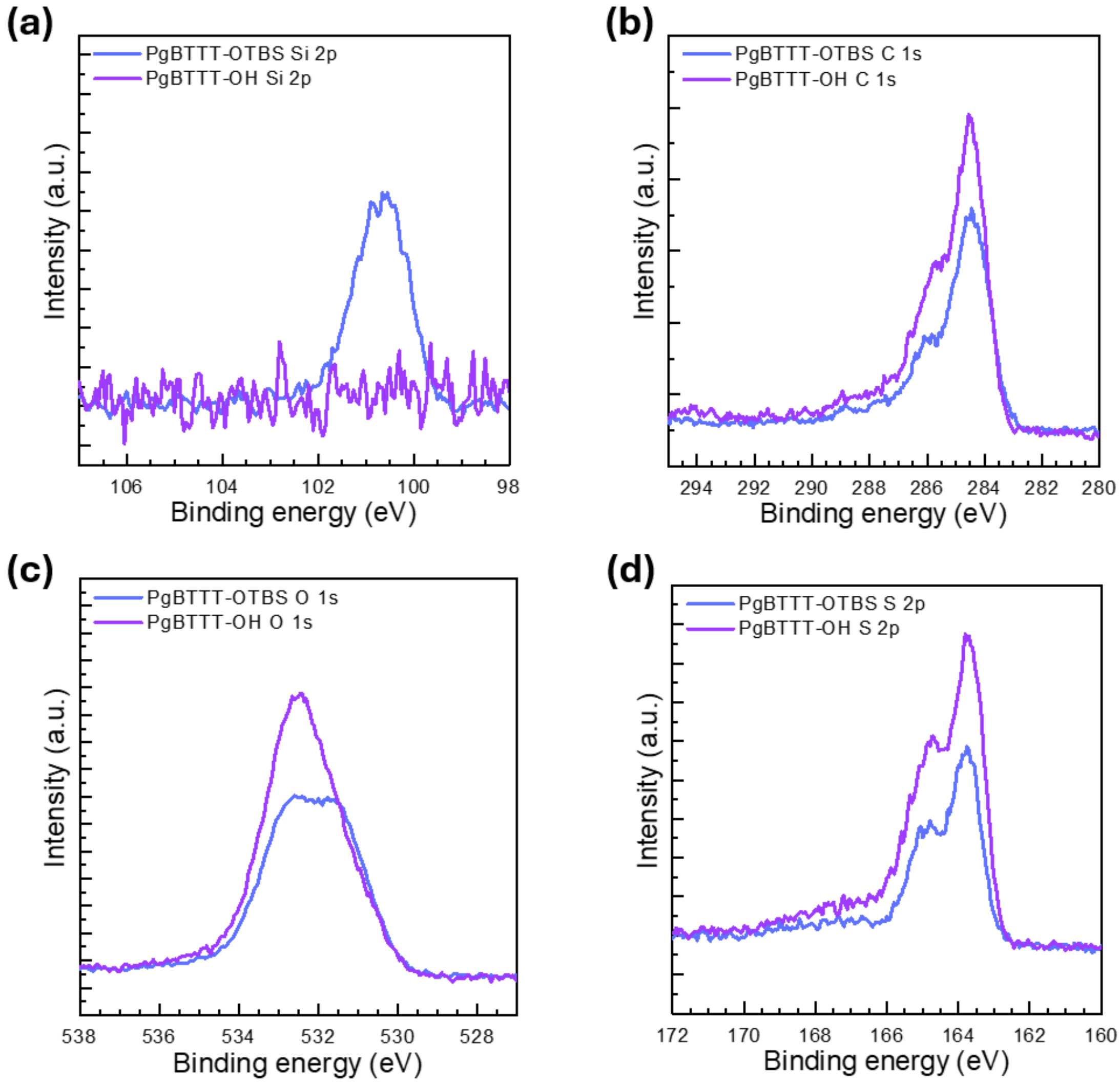

Figure S34 XPS spectra of (a) Si 2p (b) C 1s (c) O 1s and (d) S 2p of PgBTTT-OTBS and PgBTTT-OH

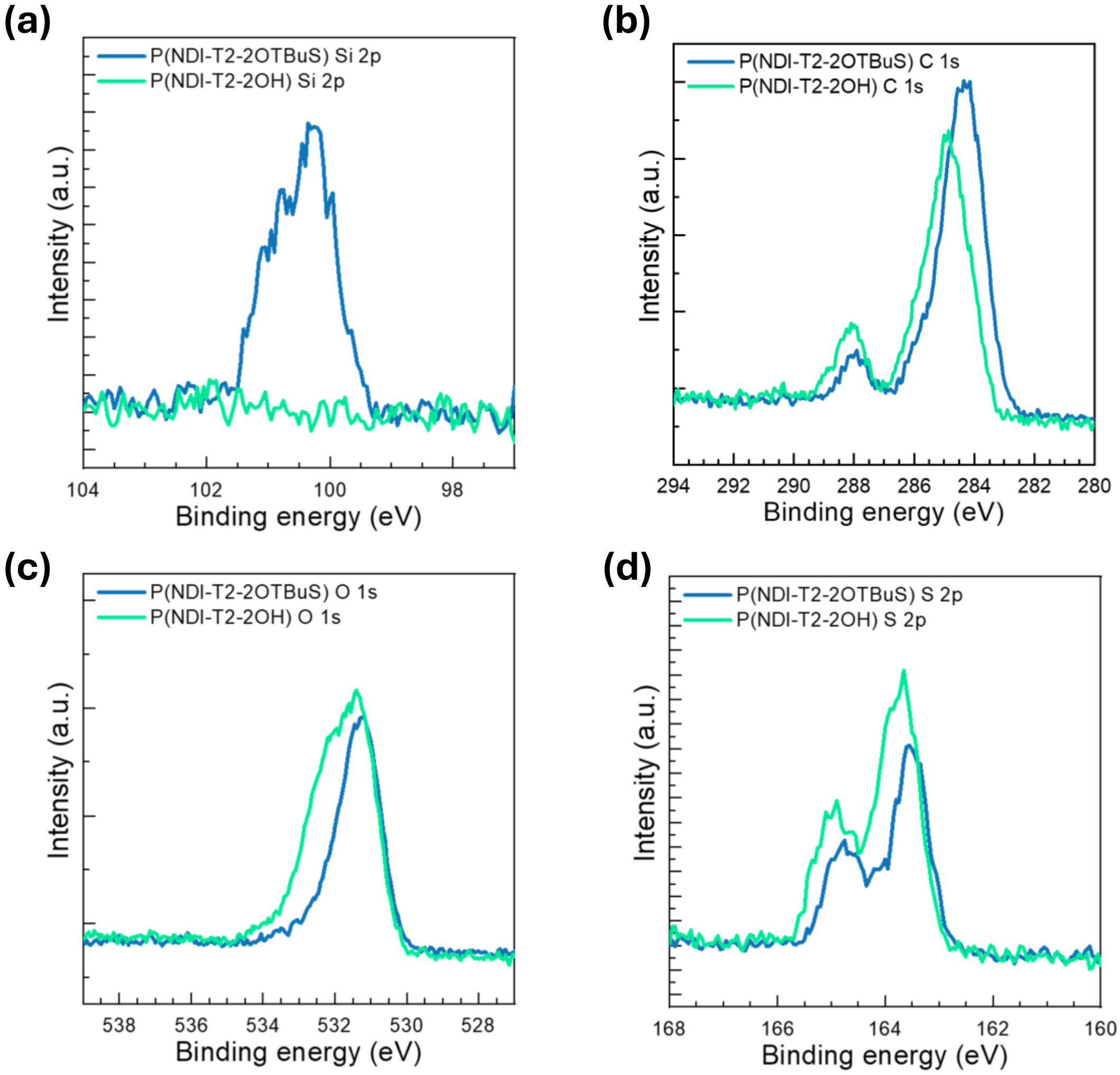

Figure S35 XPS spectra of (a) Si 2p (b) C 1s (c) O 1s and (d) S 2p of P(NDI-T2-2OTBuS) and P(NDI-T2-2OH)

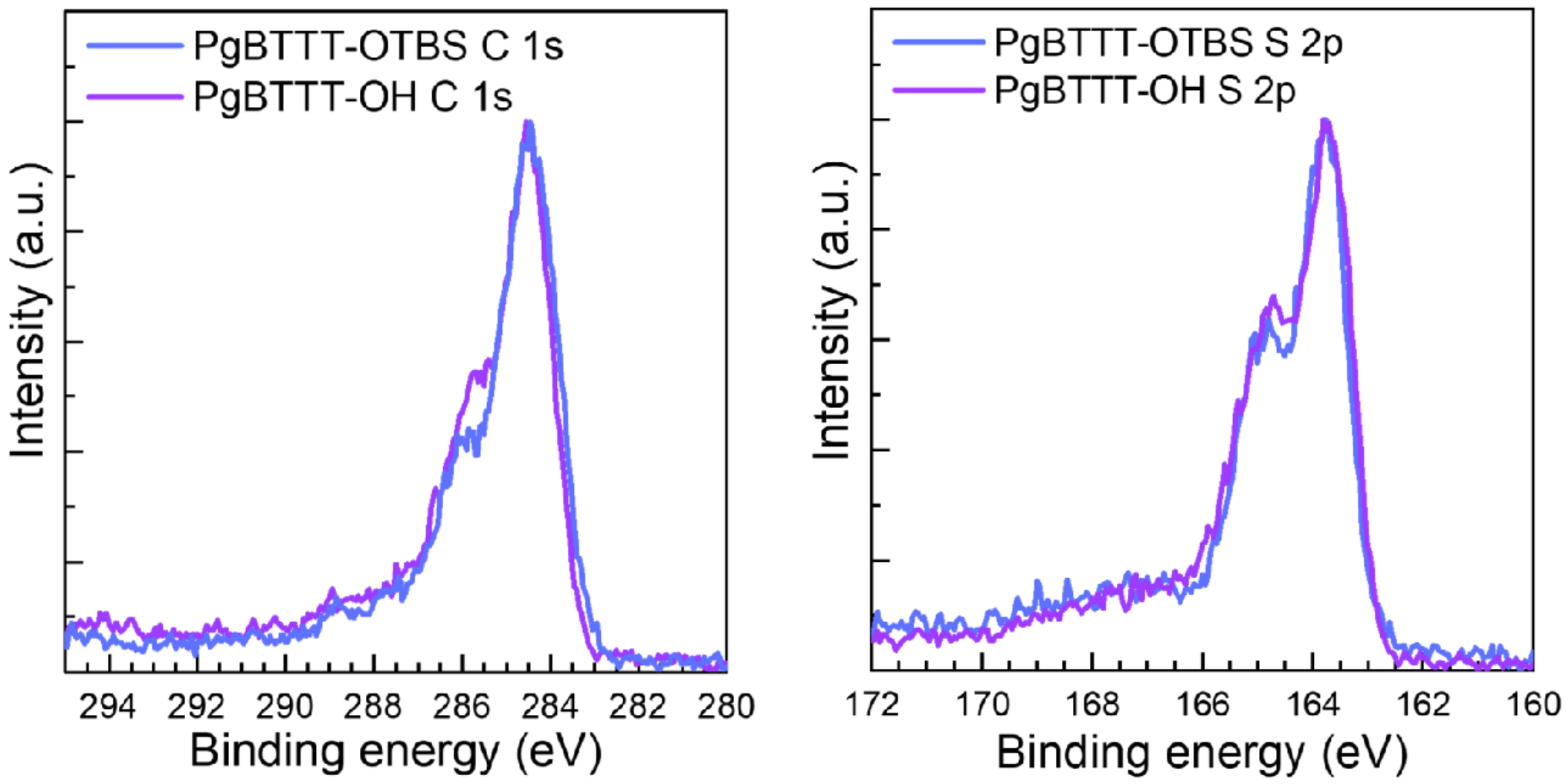

Figure S36 Normalized C 1s and S 2p XPS Spectra of PgBTTT-OTBS and PgBTTT-OH

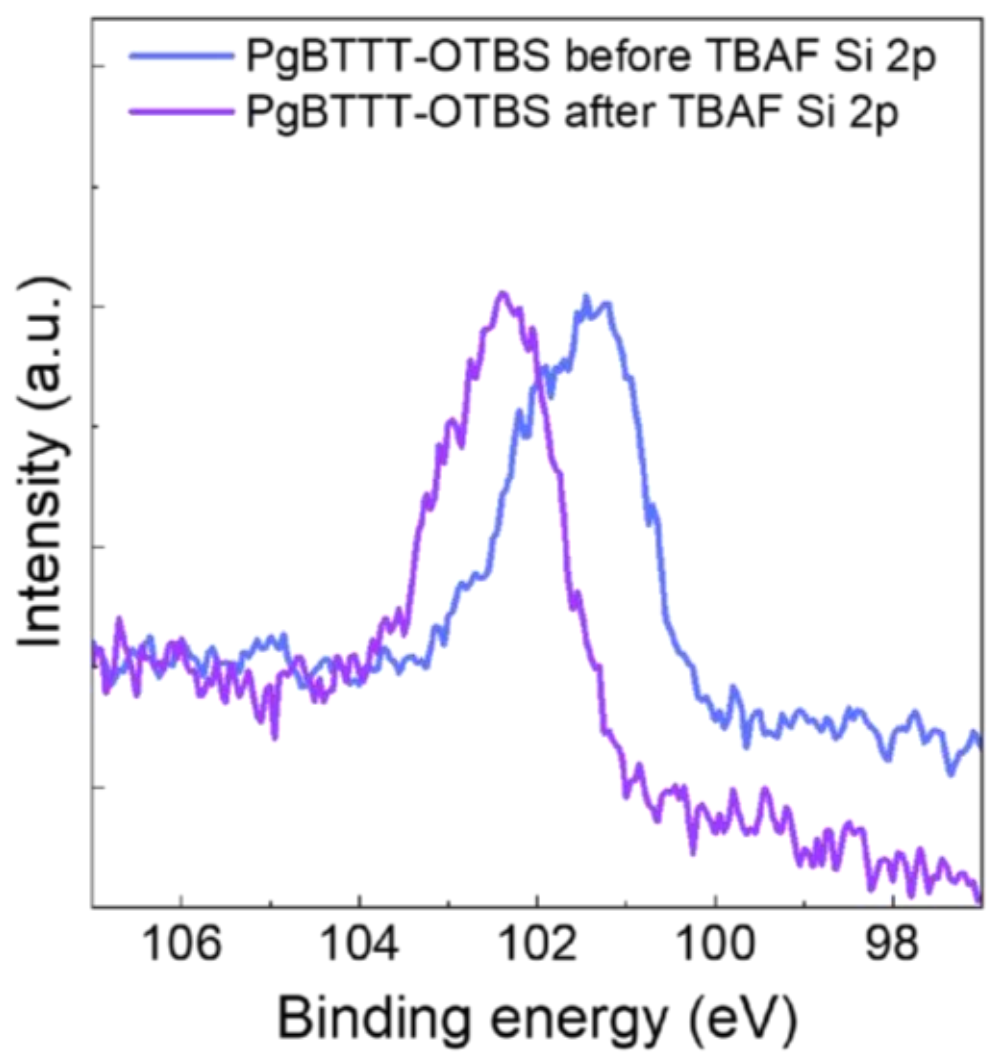

Figure S37 Si 2p XPS Spectra of PgBTTT-OTBS before and after TBAF treatment

Section 8 Comparison of reported materials figure of merits with state-of-the-art OMIEC materials

Table S3 Comparison of PgBTTT-OH and P(NDI-T2-2OH) against the state-of-the-art materials

OMIEC	type	$\frac{g_m}{Wd}/L$ $[Scm^{-1}]$	μC* [$Fcm^{-1}V^{-1}s^{-1}$]	C* [Fcm^{-3}]	M [$cm^2V^{-1}s^{-1}$]	V_T [V]	Ref.
PgBTTT	p	194	563 ± 33	164 ± 7	3.44 ± 0.13	−0.24	2
gFBT-g2T	p	298 ± 10.4	826 ± 28.8	147 ± 10	5.76	-0.33	3
p(C2g3T2-TT)	p	Not reported	1099 ± 121.0	134.2	6.60 ± 1.74	-0.09	4
p(g2T-TT)	p	56	93 ± 13	244 ± 51	0.41 ± 0.14	0.01	2,5
p(g2T2-g4T2)	p	144	522	187 ± 8	1.72 ± 0.31	0.02	6
p(g2T-T)	p	77	62 ± 24	220 ± 30	0.28 ± 0.1	-0.36	7,8
p(g3T2Te)	p	214 ± 159	483 ± 266	134±15	3.60 ± 1.92	-0.04	9
opg2T-Se	p	415 ± 22	1121 ± 51	423.3	2.99 ± 0.41	-0.02	10
**PgBTTT-OTH**	**p**	**640**	**1127**	**219**	**5.15**	**-0.12**	**This work**
**P(NDI-T2-OH)**	**n**	**0.27**	**0.67**	**361**	**1.86 × 10^{-3}**	**0.22**	

Section 9 References

(1) Willner, B. Organic Semiconductor Design, Synthesis and Testing for Solar Energy Applications.

(2) Hallani, R. K.; Paulsen, B. D.; Petty, A. J.; Sheelamanthula, R.; Moser, M.; Thorley, K. J.; Sohn, W.; Rashid, R. B.; Savva, A.; Moro, S.; Parker, J. P.; Drury, O.; Alsufyani, M.; Neophytou, M.; Kosco, J.; Inal, S.; Costantini, G.; Rivnay, J.; McCulloch, I. Regiochemistry-Driven Organic Electrochemical Transistor Performance Enhancement in Ethylene Glycol-Functionalized Polythiophenes. *J. Am. Chem. Soc.* **2021**, *143* (29), 11007–11018. https://doi.org/10.1021/jacs.1c03516.

(3) Liao, H.; Savva, A.; Marsh, A. V.; Yang, Y.; Faber, H.; Rimmele, M.; Sanviti, M.; Zhou, R.; Emwas, A.; Martín, J.; Anthopoulos, T. D.; Heeney, M. High Performance Organic Mixed Ionic-Electronic Polymeric Conductor with Stability to Autoclave Sterilization. *Angew. Chem.* **2025**, *137* (4), e202416288. https://doi.org/10.1002/ange.202416288.

(4) Yu, H.; Marks, A.; Tuladhar, S. M.; Siemons, N.; Anderson, I.; Bidinger, S.; Keene, S. T.; Quill, T. J.; Wu, R.; Gough, O.; Wu, G.; Eisner, F.; Salleo, A.; Rivnay, J.; Malliaras, G. G.; Barnes, P. R. F.; McCulloch, I.; Nelson, J. The Influence of Alkyl Spacers and Molecular Weight on the Charge Transport and Storage Properties of Oxy-Bithiophene-Based Conjugated Polymers. *Angew. Chem. Int. Ed.* **2025**, *64* (6), e202417897. https://doi.org/10.1002/anie.202417897.

(5) Giovannitti, A.; Sbircea, D.-T.; Inal, S.; Nielsen, C. B.; Bandiello, E.; Hanifi, D. A.; Sessolo, M.; Malliaras, G. G.; McCulloch, I.; Rivnay, J. Controlling the Mode of Operation of Organic Transistors through Side-Chain Engineering. *Proc. Natl. Acad. Sci.* **2016**, *113* (43), 12017–12022. https://doi.org/10.1073/pnas.1608780113.

(6) Moser, M.; Hidalgo, T. C.; Surgailis, J.; Gladisch, J.; Ghosh, S.; Sheelamanthula, R.; Thiburce, Q.; Giovannitti, A.; Salleo, A.; Gasparini, N.; Wadsworth, A.; Zozoulenko, I.; Berggren, M.; Stavrinidou, E.; Inal, S.; McCulloch, I. Side Chain Redistribution as a Strategy to Boost Organic Electrochemical Transistor Performance and Stability. *Adv. Mater.* **2020**, *32* (37), 2002748. https://doi.org/10.1002/adma.202002748.

(7) Inal, S.; Malliaras, G. G.; Rivnay, J. Benchmarking Organic Mixed Conductors for Transistors. *Nat. Commun.* **2017**, *8* (1), 1767. https://doi.org/10.1038/s41467-017-01812-w.

(8) Nielsen, C. B.; Giovannitti, A.; Sbircea, D.-T.; Bandiello, E.; Niazi, M. R.; Hanifi, D. A.; Sessolo, M.; Amassian, A.; Malliaras, G. G.; Rivnay, J.; McCulloch, I. Molecular Design of Semiconducting Polymers for High-Performance Organic Electrochemical Transistors. *J. Am. Chem. Soc.* **2016**, *138* (32), 10252–10259. https://doi.org/10.1021/jacs.6b05280.

(9) Paulsen, B. D.; Meli, D.; Moser, M.; Marks, A.; Ponder, J. F.; Wu, R.; Schafer, E. A.; Strzalka, J.; Zhang, Q.; McCulloch, I.; Rivnay, J. Enhancement of Conjugated Polymer Microstructure and Mixed-Conducting Properties via Chalcogenophene Heteroatom Substitution. *Chem. Mater.* **2024**, *36* (4), 1818–1830. https://doi.org/10.1021/acs.chemmater.3c02309.

(10) Liu, G.; Zhang, M.; Lv, J.; Wang, H.; Ma, B.; Gu, X.; Guo, Y.; Liu, Y.; Huang, H. High Iontronic Performance in Organic Electrochemical Transistors Enabled by Intramolecular Noncovalent Interactions. *Adv. Mater.* **2026**, *38* (3), e08541. https://doi.org/10.1002/adma.202508541.
